# Structural evolution of iron oxides melts at Earth’s outer-core pressures

**DOI:** 10.1038/s41467-026-75204-4

**Published:** 2026-07-10

**Authors:** Céline Crépisson, Mila Fitzgerald, Domenic Peake, Patrick G. Heighway, Thomas Stevens, Adrien Descamps, David McGonegle, Alexis Amouretti, Karim K. Alaa El-Din, Michal Andrzejewski, Sam Azadi, Erik Brambrink, Carolina Camarda, David A. Chin, Samuele Di Dio Cafiso, Ana Coutinho Dutra, Hauke Höppner, Kohdai Yamamoto, Phani S. Karamched, Zuzana Konôpková, Motoaki Nakatsutsumi, Norimasa Ozaki, Danae N. Polsin, Jan-Patrick Schwinkendorf, Georgiy Shoulga, Cornelius Strohm, Minxue Tang, Harry Taylor, Monika Toncian, Yizhen Wang, Jin Yao, Gianluca Gregori, Justin S. Wark, Karen Appel, Marion Harmand, Sam M. Vinko

**Affiliations:** 1 Department of Physics, Clarendon Laboratory, University of Oxford, Oxford, UK; 2 School of Mathematics and Physics, Queen’s University Belfast, Belfast, UK; 3 AWE, Reading, UK; 4 Graduate School of Engineering, University of Osaka, Suita, Japan; 5 European XFEL, Schenefeld, Germany; 6 Department of Physics and Astronomy, University of Manchester, Manchester, UK; 7 University of Rochester Laboratory for Laser Energetics, Rochester, NY USA; 8 Helmholtz-Zentrum Dresden-Rossendorf (HZDR), Dresden, Germany; 9 Department of Materials, University of Oxford, Oxford, UK; 10 Institute of Laser Engineering, University of Osaka, Suita, Japan; 11 National Thin Film Cluster Facility for Advanced Functional Materials, Department of Physics, University of Oxford, Oxford, UK; 12 PIMM, Arts et Metiers Institute of Technology, CNRS, Cnam, HESAM University, Paris, France

**Keywords:** Geochemistry, Core processes

## Abstract

Oxygen and other light elements comprise up to 5 wt% of the Earth’s outer-core, and may significantly influence its physical properties and the operation of the geodynamo. Here we report in situ X-ray diffraction measurements of Fe, Fe + 4.5 FeO (atomic proportion), and Fe_2_O_3_ melts at 177-440 GPa, achieved using laser-driven shock compression at an x-ray free-electron laser. The melts exhibit Fe-O coordination numbers between 4.0(0.4) and 4.5(0.4), indicating predominantly four-fold coordination environments. These coordination states are significantly smaller than those of Fe-bearing lower-mantle phases such as bridgmanite and ferropericlase. Shorter Fe-Fe interatomic distances in compressed iron oxide melts drive the denser packing relative to ambient melts, while the structural differences between Fe + 4.5 FeO and Fe_2_O_3_ melts under shock indicate that the oxidation state modulates oxygen solubility in liquid Fe. At 177 GPa ( ~ 380 km below the core-mantle boundary) and 3800 K, Fe_2_O_3_ melts exhibit higher Fe-O coordination, suggesting that local variations in oxygen content could contribute to the stratification in the uppermost outer-core inferred from seismological and geomagnetic observations.

## Abstract (web summary)

Liquid diffraction under shock compression reveals structures of Fe, Fe+4.5 FeO, and Fe_2_O_3_ melts at Earth’s outer-core pressures. The results retrieve low Fe-O coordination numbers of 4.0–4.5 along with variations in function of pressure.

## Introduction

The Earth’s iron-rich outer core contains up to  ~ 5% oxygen, together with other light elements, such as S, Si, C, and H, incorporated into liquid Fe during core formation through the segregation of metal and silicates from a primordial magma ocean^1,2^. Similar enrichment has been inferred for Mars, where seismological observations suggest that both the liquid and solid cores contain O, S and C^3^. Within the Earth, convection of this light-element-bearing liquid iron sustains the geodynamo, generating the magnetic field that shields the planet’s surface^4^. As a magnetic field plays a central role in preserving planetary atmospheres and surface environments, its detection is often considered a primary indicator of potential habitability in exoplanets. Recent studies further suggest that most super-Earths host at least partially molten metallic cores^5^, comparable to Earth’s. Understanding how such cores evolve and generate magnetic fields is therefore essential for assessing the interior dynamics and habitability of terrestrial exoplanets.

Light elements dissolved in the liquid core strongly influence its transport properties—such as viscosity and thermal and electrical conductivities—which are key parameters governing magnetic field generation^4^. During planetary evolution, crystallisation of the solid inner core expels these light elements into the overlying liquid, where their upward diffusion and accumulation provide a sustained source of compositional buoyancy that drives outer-core convection^4^.

Seismological analyses of body waves and normal modes reveal anomalously low velocities at the top of the Earth’s outer core, suggesting the presence of a stably stratified, non-convecting layer that could extend up to  ~ 450 km in thickness^6^, though its existence remains debated^7^. Independent evidence from geomagnetic fluctuations similarly indicates a stratified layer approximately 140 km thick^8,9^. The origin of this stable region remains uncertain, with both thermal^10,11^ and compositional^12–14^ mechanisms proposed. A preferential enrichment in oxygen at the top of the outer core relative to other light elements^14^, combined with non-ideal mixing behaviour^13,15^, has been suggested as a possible explanation for the observed reductions in seismic velocity and density. Oxygen partitioning models further imply that an early O-rich layer may have formed just beneath the core-mantle boundary, indicating that the early core was initially undersaturated in oxygen^16^. Changes in the Fe-O bonding environment within the outer-core have also been inferred from the B1-B2 phase transition of FeO near 240 GPa^17^, while ab initio molecular dynamics simulations of Fe_0.96_O_0.04_ indicate pressure-induced modifications of Fe-O coordination consistent with evolving bonding behaviour under core conditions^18^.

Despite extensive theoretical work, our current understanding of the melt structure of Fe-O under Earth’s outer-core conditions remains entirely based on ab initio calculations^18–21^. Our previous work on Fe_2_O_3_ did not allow to retrieve interatomic distances and coordination numbers due to reduced Q range^22^, and no other experimental measurement exists for iron oxide melts at pressures corresponding to the outer-core (136–330 GPa)^23^. Structural information for pure Fe melt has been obtained up to 275(9) GPa under laser-driven shock compression^24^, whereas Fe_0.92_O melt has been examined only up to  ~ 90 GPa by X-ray diffraction under static compression^25^. The scarcity of experimental data primarily reflects the extremely high melting temperatures of Fe-O systems, which make in situ structural measurements challenging under static compression. Laser-driven shock compression, by contrast, enables access to extreme pressure-temperature conditions, albeit on nanosecond timescales. Although kinetic effects can lead to differences from static phase equilibria (as seen recently in Fe_2_O_3_^26^), melting occurs on sub-nanosecond timescales above the liquidus^27^, allowing the melt structure relevant to planetary interior conditions to be probed reliably using dynamic compression techniques.

**Fig. 1:**
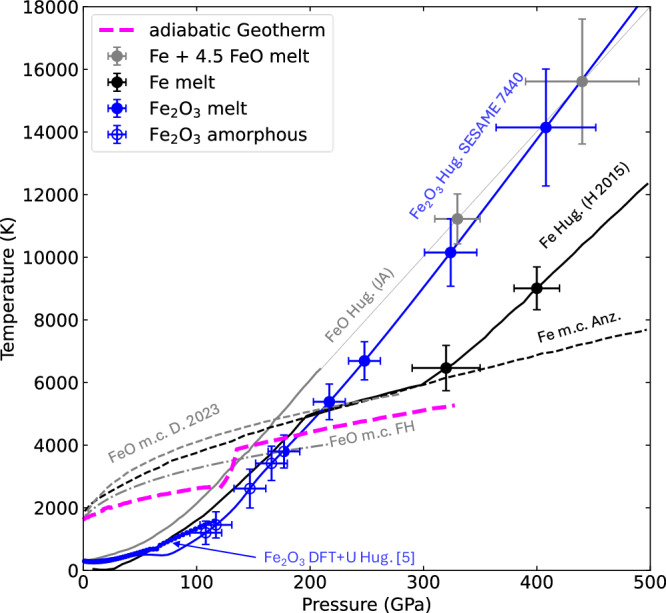
Pressure–temperature conditions of our data compared with the geotherm^72–74^. Temperatures are estimated from SESAME 7440^30^, consistent with previous Density Functional Theory (DFT+U) calculations^22^. Temperature error bars correspond to the temperature calculated at the lower and upper bounds of the pressure uncertainty. The FeO Hugoniot (Hug.) (dashed grey line) is linearly extrapolated from Jeanloz and Ahrens (JA)^29^ and the Fe Hugoniot from DFT calculations (H 2015)^28^. Melting curves (m. c.) are shown for Fe (Anz.)^75^ and FeO (D. 2023^40^ and FH^39^). The half-filled Fe_2_O_3_ data point at 177(14) GPa could be either liquid or amorphous.

**Fig. 2:**
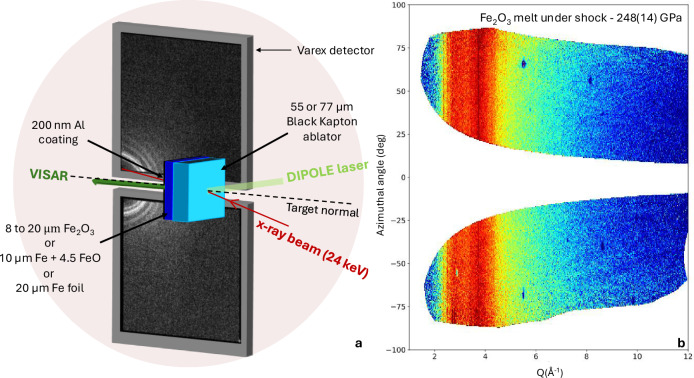
Experimental setup used in the present work. **a** Experimental setup at the High Energy Density (HED) endstation of the European X-ray Free Electron Laser facility (EuXFEL), showing a 2D raw diffraction image of an Fe_2_O_3_ target at ambient conditions prior to shock. The DiPOLE 100-X laser is shown in light green, the Velocity Interferometer System for Any Reflector (VISAR) probe in dark green, and the X-ray beam in red. Fe_2_O_3_ and Fe + 4.5 FeO layers were deposited on a black Kapton ablator via plasma sputtering, while the Fe foil was glued to the ablator. **b** Dewarped 2D X-ray diffraction (XRD) image from the Varex detectors for Fe_2_O_3_ melt under shock probed around 0.1 ns before the shock exits the target.

In this work, we use laser-driven shock compression to experimentally determine the evolution of the Fe-O bonding environment across the Earth’s outer-core pressure range in Fe^3+^ and Fe^2+^-rich iron oxide melts. We report measurements of the melt structure and density of Fe, Fe + 4.5 FeO (55.0(0.4) at% Fe and 45.0(0.4) at% O on average, as detailed in Supplementary Data 3, equivalent to Fe + 4.5 FeO in atomic proportion, assuming stoichiometric FeO composition, with Fe and FeO homogeneously distributed across the sample), and Fe_2_O_3_. Further details on initial samples are given in Supplementary Information, section “Targets Characterisation”. We study pressures ranging from 177 (14) up to more than 440(50) GPa, i.e., pressures directly relevant to the Earth’s outer core. Shock temperature could not be directly measured, and approximations are needed to evaluate temperature along the Hugoniot. In this work, we used previously calculated temperatures along the Hugoniot curve of Fe^28^, FeO^29^ and Fe_2_O_3_ (SESAME 7440^30^ and DFT+U^22^), resulting in temperatures between 3800(500)–16,000(2000) K, as shown in Fig. 1. Temperature error bars given in the text correspond to the temperature calculated at the lower and upper bounds of pressure uncertainty. We note that the Fe_2_O_3_ data points at 166(14), 177(14), and 217(14) GPa, and the Fe data point at 320(30) GPa, have a temperature within 1000 K of the geotherm. The primary diagnostic is in situ X-ray diffraction (XRD) measured in transmission from laser-driven, shock-compressed samples at the High Energy Density endstation of the European X-ray Free-Electron Laser (EuXFEL)^31^. The XRD intensities are fully corrected for solid angle, polarisation, the different apparent thickness due to different angles of the Al filter placed over the detectors, self-attenuation, ablator contribution, and residual ambient material, as detailed in the Methods section. We note that residual ambient Bragg peaks remain in the azimuthally integrated 1D XRD lineouts when the sample is probed at maximum compression but before complete release, resulting in a small unshocked fraction still appearing in the data. A velocity interferometer system for any reflector (VISAR) measured the shock breakout time, and hydrodynamic simulations were used to derive the corresponding pressures. The experimental setup is shown in Fig. 2.

## Results

### Phase evolution and melting of Fe, Fe + 4.5 FeO and Fe_2_O_3_ along the Hugoniot

**Fig. 3:**
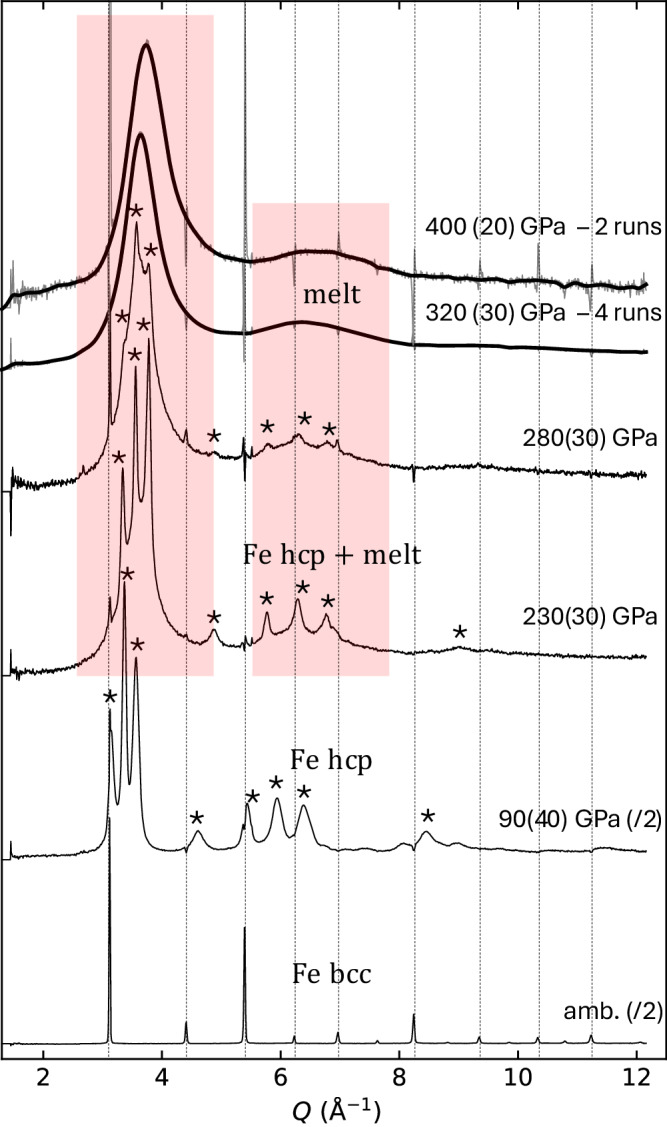
Fully corrected, azimuthally integrated 1D X-ray diffraction (XRD) lineout of Fe under laser-driven shock compression, showing the transformation from body-centred cubic (bcc) to hexagonal close-packed (hcp) Fe, followed by partial and then complete melting above 280(30) GPa. Vertical dashed lines mark residual ambient bcc peaks, and black stars indicate the main hcp peaks under pressure. Lattice parameters are provided in Section VII of the Supplementary Information and in Supplementary Data 1. Removal of remaining ambient material produces small dips at the positions of ambient peaks, reflecting sample texture and resulting in differences in relative peak intensities between the shocked and ambient samples. The diffuse scattering region is highlighted in red.

Fe transforms from a body-centred cubic (bcc) to a hexagonal close-packed (hcp) structure under shock compression at 13 GPa^32^. In Fig. 3, coexistence of hcp Fe and partial melt is observed from 230(30) GPa, while complete melting occurs above 280(30) GPa. Fully molten Fe has previously been reported under shock at 258(8) GPa^24^ and 273(2) GPa^33^, consistent with our pressure within the uncertainties.

**Fig. 4:**
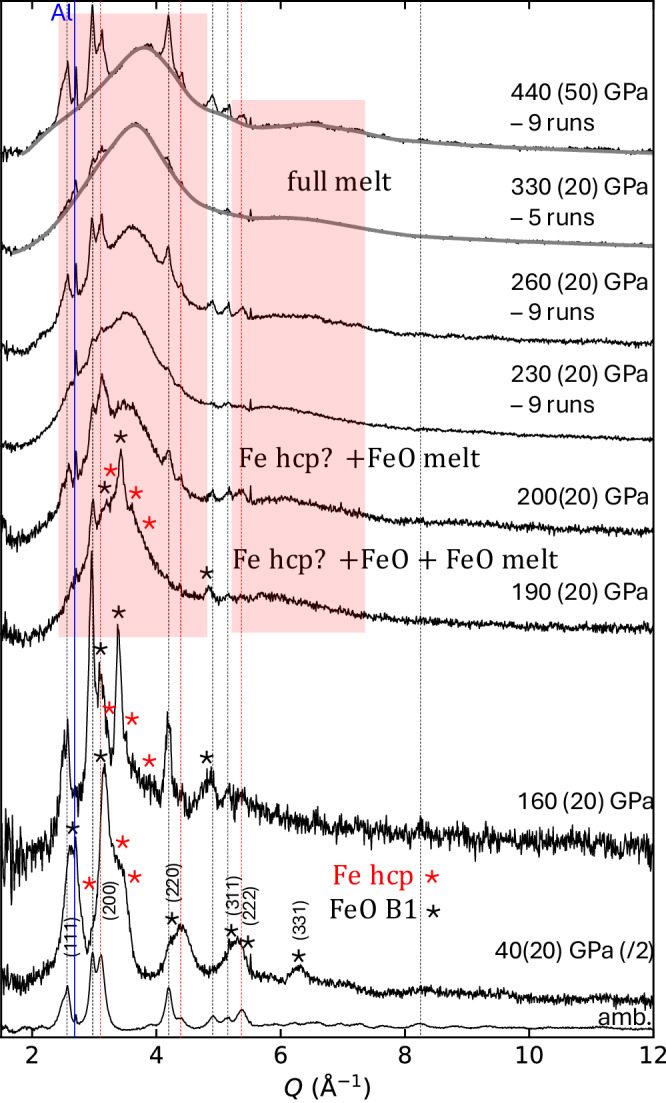
Fully corrected, azimuthally integrated 1D X-ray diffraction (XRD) lineout of Fe + 4.5 FeO under laser-driven shock compression. No phase transition is observed in FeO, which remains in the B1 (rocksalt) structure. Above 190(20) GPa, the disappearance of FeO peaks indicates melting along the Hugoniot. Hexagonal close-packed (hcp) Fe is seen at 40(20) GPa, but the hcp reflections become indistinct at higher pressures. Only data above 280 GPa (above the Fe melting point) are taken as fully molten Fe + 4.5 FeO. Vertical dashed lines in black and red mark residual ambient FeO and bcc Fe peaks, respectively, while the Al (111) peak due to the Al flash coating is marked in blue. Black stars denote the main FeO peaks, and red stars indicate hcp Fe peaks, under pressure. The diffuse scattering region is highlighted in red.

As seen in Fig. 4, FeO shows no phase transition along the Hugoniot and remains in the B1 (rocksalt) structure up to 190(20) GPa. This behaviour agrees with static compression phase diagrams^34,35^ and the pressure-temperature relation along the FeO Hugoniot^29^. A detailed analysis of volume evolution will be presented elsewhere. At 190(20) GPa, diffuse scattering appears around 3.4 and 6.2 Å^−1^ (highlighted in red in Fig. 4), indicating partial melting with Bragg peaks of the FeO B1 structure still visible. At 200(20) GPa, all FeO Bragg peaks disappear, suggesting that the Hugoniot crosses the FeO liquidus between 190–200(20) GPa. The bcc Fe present in the initial sample is observed to have transformed into hcp Fe at 40(20) GPa (bcc to hcp Fe transition is known to occur at 13 GPa^32^). At higher pressures, Fe reflections are no longer detected, possibly due to a very small domain size for hcp Fe (2–15 nm^36^) and compatibility strains between the Fe grains and the FeO matrix which could reduce the crystallinity of the Fe near its boundaries. Consequently, only data above 280 GPa where Fe is fully molten (see Fig. 3) are taken to represent complete Fe + 4.5 FeO melting. The melt signal observed above 280 GPa corresponds to a fully mixed Fe + 4.5 FeO melt, as the grain size observed on the initial sample by Scanning Transmission Electron Microscope is smaller than 10 nm (Figs. S4, 5). This small grain size allows complete mixing of Fe and FeO melt under high pressures and temperatures, based on diffusion coefficients available from ab initio molecular dynamics simulations^18^. Further discussion is provided in the Supplementary Material, section XIV.

**Fig. 5:**
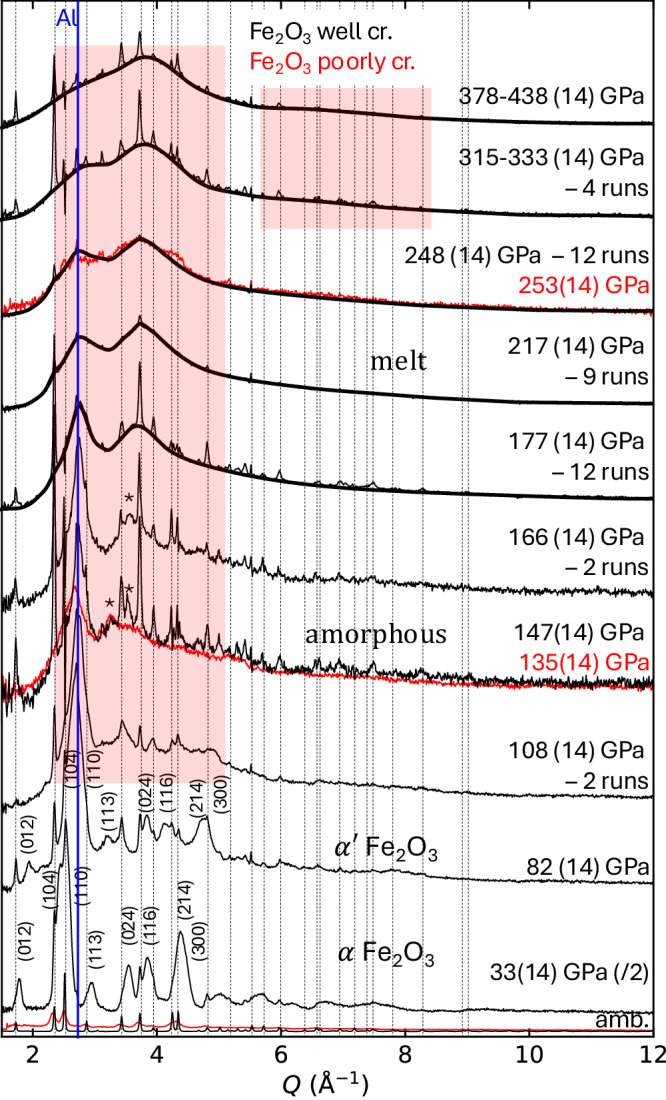
Fully corrected, azimuthally integrated 1D X-ray diffraction (XRD) lineout of Fe_2_O_3_ under laser-driven shock compression, showing: (1) the *α* to ${\alpha }^{{\prime} }$-Fe_2_O_3_ phase transition between 33(14) and 82(14) GPa; (2) amorphization from 108(14) GPa; and (3) melting from 177(14) or 217(14) GPa. Two samples of differing crystallinity were used: a well-crystalline sample (black) for most shots, and a poorly crystalline (cr.) sample (red) for two shots. Vertical black dashed lines mark residual ambient Fe_2_O_3_ peaks, while the Al (111) peak due to the Al flash coating is marked in blue. Black stars denote peaks visible in the amorphous phase. Removal of remaining ambient material produces small dips at the positions of ambient peaks, reflecting sample texture and resulting in variations in relative peak intensity between shocked and ambient samples. The diffuse scattering region is highlighted in red.

In Fig. 5, we see that Fe_2_O_3_ transforms from the *α* to the ${\alpha }^{{\prime} }$-Fe_2_O_3_ phase below 97(14) GPa. Supporting Le Bail refinements are provided in Figs. S14–16 of the Supplementary Information. This is consistent with previous shock-compression results where this isostructural transition occurred at 50–62 GPa, possibly alongside a spin transition^26^. None of the four phase transitions reported under static compression^37^ is observed. Diffuse scattering (highlighted in red in Fig. 5) and the disappearance of ${\alpha }^{{\prime} }$ Bragg peaks are seen from 108(14) GPa, indicating amorphisation. This is consistent with an earlier study, where amorphisation occurred at 122(3) GPa^22^, at temperatures too low to induce equilibrium melting. At 108(14) GPa, the temperature is estimated to be 1200(400) K according to SESAME 7440^30^ and 1400(300) K according to DFT+U^22^: this temperature remains below 1873 K, the melting point of Fe_2_O_3_ at ambient conditions^38^, and thus melting is unlikely. At 147(14) and 166(14) GPa, two peaks at 3.2 and 3.5 Å^−1^ (marked by black stars in Fig. 5) appear alongside the amorphous phase. These peaks intensify upon release and may correspond to residual ${\alpha }^{{\prime} }$; further discussion is provided in Sections IX and X of the Supplementary Information.

The comparison of two Fe_2_O_3_ samples with identical composition but differing initial crystallinity (Fig. 5) allows us to distinguish between amorphous and molten phases. At 135–147(14) GPa, the samples display distinct diffraction signatures, consistent with an amorphous or partially amorphous phase that retains features of the original crystallinity. In contrast, at 248–253(14) GPa, both samples exhibit nearly identical signals, indicative of complete melting. We thus conclude that Fe_2_O_3_ is amorphous at 147(14) GPa, and likely also at 166(14) GPa, as the corresponding patterns are similar (see Fig. 5 and section X of the Supplementary Information for more details). From 217(14) GPa onwards Fe_2_O_3_ is fully molten [XRD signatures at 217(14) and 248(14) GPa being nearly identical].

The position of the main amorphous peak at  ~ 2.74 Å^−1^ remains constant from 108(14) to 166(14) GPa, consistent with previous observations for amorphous Fe_2_O_3_^22^. Between 166(14) and 177(14) GPa, this peak broadens and decreases markedly in intensity, accompanied by the disappearance of all Bragg reflections. This behaviour indicates that the Fe_2_O_3_ Hugoniot crosses the solidus or liquidus near this pressure. A similar decrease and broadening of the first diffraction peak was previously observed between 145(12) and 151(12) GPa^22^. The higher transition pressure observed here may be due to the use of a black Kapton ablator instead of Parylene-N as used previously^22^. Parylene-N could have allowed for slight laser preheating, leading to melting at lower pressures along the Hugoniot.

Whether the structural change between 166(14) and 177(14) GPa corresponds to partial or complete melting remains uncertain. The Fe_2_O_3_ data point at 177(14) GPa and 3800(500) K (estimated from SESAME 7440^30^) lies close to the FeO melting curve of Fu and Hirose^39^, but is significantly below the temperatures reported by Dobrosavljević et al.^40^. The state of the sample at this pressure therefore remains ambiguous, and further comparison of the two Fe_2_O_3_ samples at 177(14) GPa will be required to resolve whether full melting occurs.

### Structure and density of Fe, Fe + 4.5 FeO and Fe_2_O_3_ melts under shock compression

**Fig. 6:**
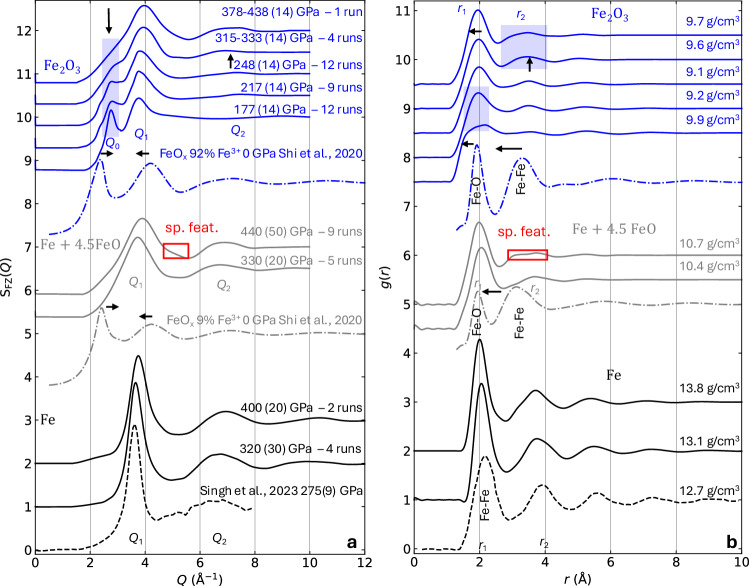
Liquid diffraction results. **a** Faber-Ziman structure factor S_FZ_(*Q*) and **b** corresponding pair distribution function *g*(*r*) for Fe, Fe + 4.5 FeO, and Fe_2_O_3_ melts, available in Supplementary Data 4. Data are shifted vertically by 0.5 or 1 for clarity. The density was refined during the calculation of *g*(*r*) using an iterative procedure^42^, as detailed in Methods. Denser packing of the melts is primarily explained by a decrease in Fe-Fe interatomic distances in Fe + 4.5 FeO, and Fe_2_O_3_ melts under shock, relative to the 0 GPa (ambient) melt from Shi et al.^47^. For Fe and Fe + 4.5 FeO, the *g*(*r*) shifts toward shorter interatomic distances with increasing pressure. In Fe_2_O_3_, two distinct changes (highlighted in blue) are observed: first, between 177(14) and 217(14) GPa, where the *Q*_0_ peak decreases and the first oscillation (*r*_1_) changes shape and width; and second, between 315–333(14) GPa, where the *Q*_2_ feature becomes pronounced, *r*_2_ increases, and *r*_1_ becomes sharper and more intense. The spurious features (sp. feat.) in high-pressure Fe + 4.5 FeO melt data (marked in red) arise from incomplete subtraction of ambient contributions.

To quantify the structural evolution and density of the melts under extreme conditions, we derived Faber-Ziman structure factors S_FZ_(*Q*) in Fig. 6 from the smoothed XRD lineouts shown in Figs. 3–5. The corresponding pair distribution functions *g*(*r*) and melt densities were then obtained using established iterative procedures^41–43^. This approach enables direct assessment of interatomic distances and coordination environments in the shocked liquids. Full details of the liquid diffraction analysis are provided in the Supplementary Information, including assessment of uncertainties based on the study of a commercial metallic glass from Goodfellow (Fe_78_B_13_Si_9_), and comparison of present results on Fe melt with previous experimental and theoretical works.

**Fig. 7:**
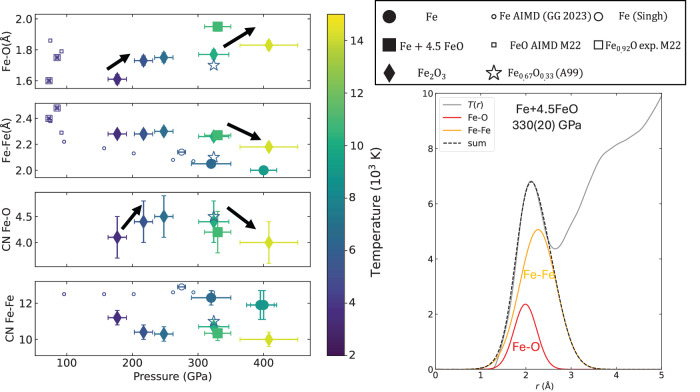
Interatomic distances and coordination numbers (CN) for Fe, Fe + 4.5 FeO, and Fe_2_O_3_ melts. Fits were performed on ${{{\mathcal{T}}}}(r)$, as detailed in the Methods. The O-O contribution is negligible and therefore excluded. The Fe-O CN for iron oxide melts under shock ranges between 4.0(0.4) and 4.5(0.4), with Fe-O bond lengths between 1.61 and 1.95 Å. Two structural changes are evident for Fe_2_O_3_ between 177(14) and 217(14) GPa, and again above 315–333(14) GPa. These correspond to variations in Fe-O distance and Fe-O CN, and are indicated by black arrows. The results agree well with previous ab initio molecular dynamics simulation studies (AIMD) of Fe (GG 2023)^44^, Fe_0.92_O (M22)^25^, and Fe_0.67_O_0.33_ (A99)^19^ melts, as well as experimental (exp.) shock data for Fe (Singh)^24^ and Fe_0.92_O (M22)^25^. Temperature estimates are based on SESAME 7440^30^ for Fe_2_O_3_, a linear extrapolation of the FeO Hugoniot^29^ for Fe + 4.5 FeO, and the Hugoniot for Fe calculated by Density Functional Theory (DFT)^28^.

The structure factors of Fe melt are consistent with those reported by Singh et al.^24^, obtained from XRD measurements under laser-driven shock compression on a synchrotron (see Fig. 6). Liquid diffraction peaks *Q*_1_ and *Q*_2_ (displayed in Fig. 6) shift toward higher scattering vectors with increasing pressure, corresponding in the pair distribution function to a decrease in interatomic distances (*r*_1_, *r*_2_ displayed in Fig. 6) and thus shorter Fe-Fe bonds. We obtain Fe-Fe interatomic distances of 2.05(0.03) Å at 320(30) GPa and 2.00(0.03) Å at 400(20) GPa, in agreement with ab initio molecular dynamics simulations of liquid Fe under pressure^44^, which report 2.05 Å at 323 GPa and 6370 K (Fig. 7). The Fe-Fe coordination numbers (CN) derived here are 12.3(0.4) at 320(30) GPa, while the data point at 400(20) GPa has too low shock fraction to extract CN ( ~ 49% resulting in greater uncertainty in the corrected XRD intensity according to Wark et al.^45^).

The structure factor of Fe + 4.5 FeO melt under shock differs markedly from that of FeO melt at ambient pressure, as shown in Fig. 6. The two diffraction peaks characteristic of ambient FeO-type melts^46,47^ merge into a single feature, *Q*_1_. Similarly, the first two oscillations in the ambient pair distribution function, attributed to Fe-Fe and Fe-O interatomic distances, coalesce into a single peak (*r*_1_), reflecting a pronounced decrease in the Fe-Fe distance. Both *r*_1_ and *r*_2_ shift toward shorter interatomic distances with increasing pressure, consistent with denser atomic packing in the melt. The pair distribution function remains otherwise similar between 330(20) and 440(50) GPa, apart from this systematic contraction, indicating that the Fe-O bonding environment is largely preserved with pressure. At 330(20) GPa, we find the Fe-O CN to be 4.2(0.4), a Fe-O distance of 1.95(0.03) Å, and an Fe-Fe distance of 2.27(0.03) Å (Fig. 7).

Similarly to what is observed for Fe + 4.5 FeO melt, the structure factor of Fe_2_O_3_ melt under shock is very different from Fe_2_O_3_-type melt at ambient pressure (Fig. 6). The two principal diffraction peaks, *Q*_0_ and *Q*_1_, evolve in opposite directions: *Q*_0_ diminishes with pressure and nearly vanishes at 378–438(14) GPa, whereas *Q*_1_ shifts from 3.77 to 3.97 Å^−1^ between 177(14) and 378–438(14) GPa. The absence of a significant shift in *Q*_0_, together with the emergence of a clear *Q*_2_ feature above 315–333(14) GPa, results in a structure factor that closely resembles that of the Fe + 4.5 FeO melt at similar pressures.

In the corresponding pair distribution functions (Fig. 6), Fe-O and Fe-Fe contributions merge into a single peak (*r*_1_), indicating increased structural compaction akin to that seen in Fe + 4.5 FeO melt. Between 177(14) and 217(14) GPa, the broadening and reshaping of *r*_1_ reflect a rise in the Fe-O CN from 4.1(0.4) to 4.4(0.4), and a lengthening of the Fe-O bond from 1.61(0.03) to 1.73(0.03) Å. The Fe-Fe distances remain at 2.30(0.03) Å, as can be seen from Fig. 7. From 217(14) to 315–333(14) GPa, the structure stabilises, with *r*_1_ remaining nearly constant (1.96–1.98 Å). At higher pressures, *r*_1_ shifts to shorter distances (down to 1.94 Å) and *r*_2_ becomes more pronounced, indicating a more densely packed network. These changes correspond to a reduction in the Fe-O CN to 4.0(0.4), a modest expansion of Fe-O bonds to 1.83(0.03) Å, and a contraction of Fe-Fe distances to 2.17(0.03) Å.

**Fig. 8:**
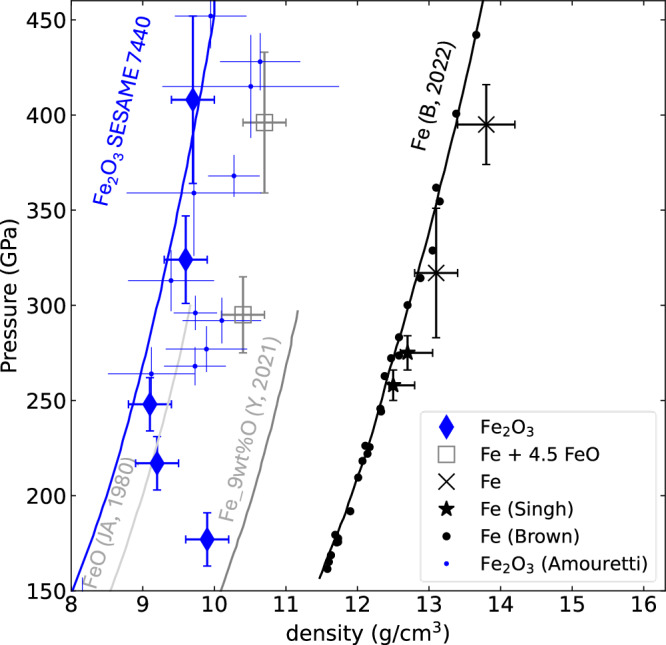
Densities extracted from liquid diffraction analyses. For Fe, results are compared with gas-gun data (Brown)^48^, laser-driven shock data (Singh)^24^, and theoretical calculations (B,2022)^76^. For Fe + 4.5 FeO, results are compared with gas-gun data for Fe_0_. 94O (JA, 1980)^29^ and Fe containing 9 wt% O (Y,2021)^49^. For Fe_2_O_3_, results are compared with SESAME 7440^30^ and laser-shock data (Amouretti)^68^.

The extracted densities shown in Fig. 8 are in good overall agreement with previous theoretical and experimental data, validating our data analysis approach. For Fe, results agree with liquid diffraction measurements^24^ and are slightly shifted toward higher density relative to the optical Hugoniot data of Brown et al.^48^. Fe_2_O_3_ densities are consistent with the SESAME 7440 equation of state and previous optical Hugoniot measurements within uncertainty above 248(14) GPa. Discrepancies at lower pressures are discussed below. As expected, densities for Fe + 4.5 FeO fall between those of FeO^29^ and Fe with 9 wt% O^49^.

## Discussion

We resolve the Fe-O bonding environment between 177 and 438 GPa, and 3800(500)–14,000(2000) K based on temperature estimates from SESAME 7440^30^ for Fe_2_O_3_, and a linear extrapolation of the FeO Hugoniot^29^ for Fe + 4.5 FeO. As we show in Fig. 7, under these extreme conditions, the Fe-O CN ranges between 4.0(0.4) and 4.5(0.4), and the Fe-Fe CN ranges between 10.0(0.4) and 11.2(0.4), for both Fe + 4.5 FeO and Fe_2_O_3_ melts. These values are consistent with ab initio molecular dynamics simulations of Fe_0.67_O_0.33_ melt by Alfè et al.^19^, which reported an Fe-O CN of 4.5 and an Fe-Fe CN of 11 at 324 GPa and 6000 K, although our data above 247(14) GPa were collected at significantly higher temperatures, as seen in Fig. 1. The effect of temperature on melt structure at such extreme pressures remains poorly constrained. However, we note that no significant changes have been observed in the Fe-O bonding environment of Fe_0.96_O_0.04_ at 135 GPa between 4600 and 5500 K^18^.

Our results suggest that the previously proposed increase in Fe-O coordination number from 6 to 8 in liquid FeO near 240 GPa, by analogy with the B1-B2 solid-state transition in FeO, is unlikely to hold^17^. Furthermore, no significant change is observed in the Fe-O bonding environment from 217(14) to 315–333(14) GPa, as shown in Fig. 7. Therefore, no layering is expected to result from changes in oxygen incorporation in liquid Fe between approximately 780 km below the Core-Mantle Boundary (CMB) and the inner-core boundary.

At ambient pressure, the Fe-O CN ranges from 4.5 in FeO-type melts to 5 in Fe_2_O_3_-type compositions^47^. Theoretical calculations indicate that these values arise from mixed 4- and 5-fold contributions in FeO-type melts and from mixed 4-, 5-, and 6-fold environments in Fe_2_O_3_-type melts^47^. Although further modelling is needed to quantify individual contributions under shock conditions, the present Fe-O CN values of 4.0-4.5(0.4) likely reflect similar coordination distributions involving 4-, 5-, and possibly 6-fold Fe-O environments.

This finding implies that the Fe-O bonding environment in iron oxide melts differs markedly from that in major lower-mantle Fe-bearing minerals, such as ferropericlase and bridgmanite, where Fe is surrounded by 6 or 8 oxygen atoms. It has been shown that oxygen solubility increases with pressure, allowing oxygen to be significantly incorporated into liquid Fe^50^. Generally, elements are more readily incorporated into a melt if their CN is higher than in the solid phase. Alfè et al.^19^ reported Fe-O and Fe-Fe CN values similar to ours using ab initio molecular dynamics simulations, estimating an average of 9 Fe atoms and 4 to 5 O atoms surrounding each oxygen atom, with potential charge transfer between O and Fe. This high oxygen solubility is thus explained by the large number of bonds formed by oxygen atoms in liquid Fe and by its small atomic radius, which allows oxygen to enter the dense network^19^.

Comparison of the pair distribution functions for the ambient-pressure melt and the shock-compressed data shows a clear structural change. The increase in density with pressure (Fig. 8) mainly results from a strong shortening of the Fe-Fe interatomic distance by around 1 Å, from 3.1 to 3.3 Å in ambient iron oxide melts^47^ to  ~ 2.30 Å under shock compression. In both Fe + 4.5 FeO and Fe_2_O_3_ melts, the Fe-Fe distance remains nearly constant between 177(14) and 333(14) GPa at  ~ 2.30 Å. These values are consistently larger than those in pure Fe melt (Fig. 7). Ab initio molecular dynamics simulations of Fe_0.67_O_0.33_ at 324 GPa and 6000 K^19^, and of Fe_0.96_O_0.04_ at 330 GPa and 5200 K, give Fe-Fe distances about 0.2 Å shorter than our results (Fig. 7). Distances between 2.3 and 2.5 Å are also reported for Fe_0.92_O at  ~ 90 GPa and 4000 K^25^. The slightly longer Fe-Fe bonds measured here may reflect the higher temperatures reached along the Hugoniot, where temperature increases significantly with pressure.

At 378–438(14) GPa and 14,000(2000) K, a distinct shift of the *r*_1_ feature in the pair distribution function of Fe_2_O_3_ melt is observed in Fig. 6. This shift corresponds to a reduction of the Fe-Fe interatomic distance by about 0.1 Å (Fig. 7). The shortening of the Fe-Fe distance is accompanied by a decrease in the Fe-O coordination number from 4.4(0.4) to 4.0(0.4) and an increase in the Fe-O bond length from 1.77(0.03) to 1.83(0.03) Å. Between 315 and 333(14) GPa, the structure factor and pair distribution function of Fe_2_O_3_ melt begin to resemble those of the Fe + 4.5 FeO melt. This convergence reflects a concurrent reduction in Fe-O coordination and elongation of the Fe-O bond, which approaches the values observed for Fe + 4.5 FeO melt at 330(20) GPa (Fig. 7). These structural changes may signal a modification in oxidation state, potentially associated with partial dissociation of Fe_2_O_3_ into FeO and O under extreme conditions. At ambient pressure, the oxidation enthalpy of Fe_2_O_3_ is  − 5.83 eV per mole of O_2_, indicating that dissociation into 2 FeO + O requires 2.92 eV (equivalent to  ~ 23,200 K)^51^. This temperature is considerably higher than that reached under shock at 378–438(14) GPa (around 14,100 K). However, recent studies suggest that the energy required for phase dissociation decreases with increasing pressure (e.g., MgSi_2_O_3_^52^). It is therefore possible that, under the present conditions, Fe_2_O_3_ partially dissociates into an FeO-type melt and oxygen at lower temperatures around 1 eV.

The Fe-O interatomic distances exhibit a complex evolution, varying between 1.61–1.95(0.03) Å across the temperature-pressure range investigated here, as shown in Fig. 7. Values reported for Fe_0.92_O at 90 GPa and 4000 K^25^ are within this range. The Fe-O distance obtained previously via ab initio molecular dynamics for Fe_0.67_O_0.33_ at 324 GPa and 6000 K^19^, and for Fe_0.96_O_0.04_ between 135 and 330 GPa and 4600–5500 K^18^, are also consistent with our measurements.

We first note that the Fe-O interatomic distance in the Fe +4.5FeO melt is larger than in all Fe_2_O_3_ compositions examined here. Moreover, Fig. 6 shows that up to at least 315–333(14) GPa, the structure factor and pair distribution function of the Fe + 4.5 FeO and Fe_2_O_3_ melts remain distinctly different. As illustrated in Fig. 7, this difference arises from the larger Fe-O bond length [1.95(0.03) Å] and slightly lower Fe-O coordination number [4.2(0.4)] in the Fe + 4.5 FeO melt. A similar contrast between Fe_2_O_3_ and FeO melts is observed at ambient pressure^47^. At 0 GPa, Fe^3+^-rich melts exhibit a higher Fe-O coordination number (5) and a shorter Fe-O bond length (1.92 Å), whereas Fe^2+^-rich melts show lower coordination (4.5) and longer Fe-O bonds (1.95 Å)^47^. These structural differences between Fe^3+^- and Fe^2+^-rich melts thus persist under extreme pressures and temperatures. They are, therefore, likely to be present at lower pressures as well, relevant to magma oceans (up to  ~ 60 GPa for the Earth^53,54^). The presence of Fe^3+^ in the early magma ocean is of particular importance for understanding the oxidation state of the Earth^55^ and Super-Earths^56^. In peridotitic melt, Fe^2+^ has been shown to act as a network modifier, while Fe^3+^ presents a more ambiguous role with a higher Fe-O CN^57^, in agreement with our results. The differential solubility of ferric and ferrous iron within the magma ocean is a key parameter for retracing the evolution of the oxidation state of the Earth and Super-Earths.

The Fe-O interatomic distance in Fe_2_O_3_ melt increases overall along the Hugoniot, from 1.61(0.03) to 1.83(0.03) Å. As shown in Fig. 7, the most pronounced increases occur between 177(14) and 217(14) GPa, and again above 315–333(14) GPa. The increases observed at 177(14) and 217(14) GPa are accompanied by a rise in the Fe-O coordination number from 4.1(0.4) to 4.4(0.4). A similar structural change has been reported for B_2_O_3_ between 4.3 and 5.7 GPa^58^. In that system, the structure factor exhibits a decrease in the first diffraction peak, similar to the reduction of the *Q*_0_ peak observed here between 177 and 217(14) GPa (Fig. 6). This behaviour is uncommon among oxide melts under compression. Interestingly, in B_2_O_3_, the increase in the B-O coordination number from 3 to 4 and the elongation of the B-O bond from 1.40 to 1.45 Å are associated with a viscosity decrease of nearly four orders of magnitude^58^. The change in Fe-O bonding environment observed here between 177(14) GPa (3800(500) K) and 217(14) GPa (5400(700) K) occurs at pressure and temperature conditions close to the geotherm (Fig. 1), and may therefore be of particular geophysical interest, as it corresponds to a depth of roughly 380 km below the core-mantle boundary^23^. The increase in Fe-O interatomic distances observed above 177(14) GPa suggests a slight decrease in oxygen solubility, linked to the longer and thus weaker bonds at depths greater than 380 km below the CMB. It has been proposed that oxygen enrichment at the top of the Earth’s outer core, originating from a partially molten, FeO-rich layer at the CMB, could explain the observed low velocity and low density^14^. Nevertheless, even assuming a maximum diffusion coefficient of 3.3 x 10^−8^ m^2^/s for oxygen within liquid Fe^18^, oxygen diffusing from the CMB is unlikely to reach a depth of more than 100 km, even over the entire history of the Earth. A thicker stable layer is, however, explained by a change in oxygen solubility at approximately 380 km depth, leading to higher oxygen content within the first 380 km of the outer core. If the observed change is accompanied by a slight drop in viscosity, similar to results seen in B_2_O_3_^58^, convection will not affect this outermost, more viscous layer. This mechanism therefore explains the presence of a relatively thick, stratified layer inferred from seismological and geomagnetic observations^6^. Nevertheless, it remains uncertain whether Fe_2_O_3_ is fully molten or partially amorphous at 177(14) GPa. Partial amorphisation could account for the anomalously high density measured at this pressure, as shown in Fig. 8.

Laser-driven shock compression at XFEL facilities now enables high-accuracy liquid diffraction measurements of complex polyatomic melts. Our measurements of iron oxide at Earth’s outer-core pressures reveal a Fe-O coordination number of 4.0–4.5(0.4), distinct from major lower-mantle minerals, with significant structural differences between Fe^3+^- and Fe^2+^-rich melts. These findings constrain oxygen solubility and the local environment in the Earth’s outer core throughout its geological evolution. The anomalous diffuse scattering observed in Fe_2_O_3_ at 177(14) GPa (corresponding to a depth of  ~ 380 km) warrants further investigation to elucidate the underlying structural transformations.

## Methods

### Targets

Seven distinct target configurations were used in the shock-compression experiments.

Two Fe_2_O_3_ samples were prepared: (1) a well-crystallised 20 μm Fe_2_O_3_ layer deposited from an Fe sputtering target onto a 55 or 77 μm black Kapton ablator using plasma sputtering, and (2) a poorly crystalline 8 μm Fe_2_O_3_ layer deposited from an Fe_2_O_3_ sputtering target onto a 55 μm black Kapton ablator by the same method. In both cases, a 200–400 nm Al coating was applied to enhance reflectivity for VISAR measurements. X-ray diffraction (XRD) and Electron MicroProbe Analyses (EMPA) (Supplementary Information Section I) confirm that both samples consist of pure Fe_2_O_3_. Scanning electron microscopy reveals a homogeneous and fully dense starting material.

The Fe + 4.5 FeO targets consist of a 10 μm layer containing 80.7(0.3)wt% Fe and 18.9(0.2)wt% O (55.0(0.4) at% Fe and 45.0(0.4) at% O), deposited via plasma sputtering from an Fe target onto a 77 μm black Kapton ablator. An additional 200–400 nm Al coating was applied for VISAR reflectivity. Bragg-Brentano XRD and Electron Microprobe analyses show that the initial material contains intergrown Fe (bcc) and FeO (B1) crystals, corresponding to Fe + 4.5 FeO in atomic proportions, assuming FeO stoichiometry.

The Fe target comprises a 20 μm Goodfellow Fe foil (99.99% purity) bonded to a 77 μm black Kapton ablator. The adhesive layer is approximately 5 μm thick, with minor variations.

The Cu target used to verify pressure calibration consists of a 25 μm Goodfellow Cu foil attached to a 55 μm black Kapton ablator using a similar adhesive layer of about 5 μm.

Isolated black Kapton targets were employed to calibrate the ablator pressure at specific laser intensities. These calibration targets consist of 77 μm black Kapton coated with a 200 nm Al layer on the VISAR side.

Finally, a 25 μm commercial metallic glass from Goodfellow (Fe_78_B_13_Si_9_) previously measured at I15-1 diffractometer at Diamond Light Source (data measured by Daniel Irving as part of the I15-1 mail-in service, proposal CY39017) at 76 keV^59^, was used to verify XRD data corrections and help to estimate uncertainties related to liquid diffraction analysis (described in Section XIII of the Supplementary Information).

### Experimental set-up

The experiment was performed at the High Energy Density (HED) endstation of the EuXFEL^31^, in IC2. We used the high-repetition-rate DiPOLE 100-X optical laser^31,60^ as the shock-compression driver. The frequency-doubled 515 nm laser beam was incident at 22.5^∘^ to the target normal and focused to a spot of  ~ 250 μm in diameter. Flat-top pulses of 4, 6, and 10 ns in duration delivered up to 50 J of energy to the target. Representative pulse profiles are provided in Section III of the Supplementary Information.

The x-ray probe beam, produced in self-amplified spontaneous emission (SASE) mode, had a photon energy of 24 keV and was focused to a spot size of 26 × 15 μm. It was directed at 22.5 degrees from the target normal and intersected the target at the centre of the laser focal spot. Two-dimensional X-ray diffraction (XRD) patterns were recorded using two Varex detectors. Their azimuthal and angular coverage are illustrated in Fig. 2. Before each laser shot, a preshot measurement was taken with the x-ray beam attenuated to 5% transmission and displaced laterally by 20 μm from the subsequent shot position. These preshots provided reference data under unshocked conditions.

### Integration and correction of XRD intensity

X-ray diffraction (XRD) intensities were renormalised to account for fluctuations in the XFEL beam using the in-chamber IPM diodes^31^. The data were fully corrected for solid angle, polarisation, and self-attenuation. Fluorescence from a 25 μm Rh foil (absorption edge at 23.22 keV) was employed to correct for the different apparent thickness due to different angles of the Al filter placed over the Varex detectors through a flat-fielding procedure similar to that previously applied for the same setup^61^. Two-dimensional XRD images were azimuthally integrated using the pyFAI library^62^.

To validate this procedure, we compared the azimuthally integrated XRD signal of a 25 μm-thick commercial metallic glass from Goodfellows (Fe_78_B_13_Si_9_) measured with the present setup with data acquired at beamline ID15 (Diamond Light Source) at 76 keV^59^. The two datasets show excellent agreement from 1.46 to 12.4 Å^−1^, with only minor discrepancies below 2.5 Å^−1^, as shown in Fig. S32 of the Supplementary Information.

For shock-compressed data, the residual contribution from the unshocked sample was removed following the procedure described by Wark et al.^45^. A sum of preshot measurements or a shot taken without the DiPOLE laser was used as the ambient reference. The removal is complicated by grain-to-grain variations in Fe and Fe_2_O_3_, which are textured and show changing Bragg peak intensities between shots. The Fe + 4.5 FeO samples also exhibited broad Bragg peaks, making ambient subtraction less precise. Nevertheless, the shocked fraction exceeds 79% for all liquid data, except for Fe at 400(20) GPa, where the shocked fraction is 49%.

The contribution from the black Kapton ablator was minimised using a structureless analogue for ambient black Kapton (C_22_H_10_N_2_O_5_), since no direct ambient measurement was performed. The ambient black Kapton contribution is particularly small in runs with a high shocked fraction (0–20% unshocked material remaining).

To remove the ablator contribution under shock, we measured fully shocked 77 μm black Kapton samples coated with 200 nm Al. The shocked state of black Kapton depends on the presence of an adjacent target layer, which induces a reshock, leading to higher pressures and lower temperatures than in isolated black Kapton. However, no significant variation in the compressed black Kapton XRD signal was observed between 21(1) and 122(4) GPa along the Hugoniot. We therefore consider the signal from isolated black Kapton at 122(4) GPa a suitable proxy for the ablator response. Diamond formation was detected in black Kapton at 56(2), 64(3), and 72(3) GPa, but no diamond peaks are expected under the conditions corresponding to the molten data.

All corrections described above were applied to each individual dataset prior to summation. Identical XRD signals from multiple runs were then combined to enhance the signal-to-noise ratio, a step crucial for reliable liquid diffraction analysis.

The signal-to-noise ratio (SNR) varies as a function of sample thickness and average atomic number. Consequently, the Fe dataset exhibits an overall higher SNR than the Fe_2_O_3_ and Fe + 4.5 FeO datasets. Similarly, Fe_2_O_3_ has a higher SNR than Fe + 4.5 FeO because it is significantly thicker. Variations in the SNR within a single sample are linked to the natural fluctuations of XFEL pulse intensity in SASE mode.

### Determination of interatomic distance and coordination number

For the monoatomic melt (Fe), interatomic distances were determined directly from the maximum of a skewed Gaussian fit to the first oscillation of the pair distribution function. The coordination number (CN) was obtained by integrating the area up to the first minimum of the radial distribution function, ${{{\mathcal{R}}}}(r)$, defined as

1 $${{{\mathcal{R}}}}(r)=4\pi {r}^{2}g(r){n}_{0},$$

where *n*_0_ is the atomic density.

For the polyatomic melts (Fe + 4.5 FeO and Fe_2_O_3_), the Fe-O and Fe-Fe contributions were each represented by a single Gaussian function. The parameters of these functions were optimised to reproduce the first oscillation of

2 $${{{\mathcal{T}}}}(r)=4\pi rg(r){n}_{0},$$

as shown in Fig. 7. The fitting procedure follows the approach described by Heinen and Drewitt^63^. The O-O contribution was neglected because it is small compared with the Fe-O and Fe-Fe components due to the difference in atomic numbers between O and Fe (8 versus 26).

In this model, the Gaussian area corresponds to the coordination number, and the fitted position [after correction for the offset between *g*(*r*) and ${{{\mathcal{T}}}}(r)$] yields the interatomic distance. All Gaussian fits are presented in Section XIII of the Supplementary Information. Reliable fitting could not be achieved for the Fe + 4.5 FeO dataset at 440(50) GPa, owing to the large fraction of unshocked material, which introduces spurious features in the pair distribution function (Fig. 6).

### Pressure determination

Where possible, a VISAR^64,65^ was used to determine the breakout time, i.e., the time when the shock exits the target. However, the setup was unable to provide access to the velocity history. A full summary of the VISAR measurements is given in the Supplementary Data 1.

A calibration curve relating the ablator pressure to laser intensity was established using isolated 77 μm-thick black Kapton samples coated with 200 nm of Al and verified using Cu targets under shock (details are provided in Section II of the Supplementary Information). Breakout times were measured by VISAR, and pressures were derived from the Hugoniot relation for polyimide^66^. The main sources of uncertainty include variations in black Kapton thickness (measured by touch probe to be between 76.6 and 77.3 μm), the coefficients of the polyimide Hugoniot equation, and the interpolation of data to fit a power-law function. The maximum uncertainty on the pressure is estimated at  ± 14 GPa. This calibration curve was used to determine the ablator pressure corresponding to a given laser intensity.

Pressures within the Fe, FeO (as a proxy for Fe + 4.5 FeO), and Fe_2_O_3_ targets were derived using the impedance-matching method^67^. The equation of state (EOS) for the black Kapton ablator was taken from SESAME 7770, while those for FeO and Fe_2_O_3_ were based on prior experimental data^29,68,69^. For Fe, SESAME 2140 and 2145 were used.

Hydrodynamic simulations were performed using the code MULTI^70^ to independently estimate the shock pressures in Fe_2_O_3_ and Fe + 4.5 FeO. The simulations used SESAME 7440^30^ for Fe_2_O_3_ and the FeO EOS of Jeanloz and Ahrens^29^, with real pulse profiles as input. A numerical intensity factor ( ~ 0.5–0.7) was applied to the experimental laser intensity to match the measured breakout times. The black Kapton ablator was again modelled using SESAME 7770. When breakout times were unavailable, we used the intensity multiplier determined on the closest shot performed at similar laser conditions (detailed in Supplementary Data 10).

Pressures derived from impedance matching and hydrodynamic simulations agree within 20 GPa for Fe + 4.5 FeO, and within 14 GPa for Fe_2_O_3_. These values are used as estimates of pressure uncertainty. For the Fe targets, only the impedance-matching method was applied, as the non-uniform glue thickness between the Fe foil and the black Kapton ablator introduced additional variability in breakout times. The dominant uncertainty arises from the ablator pressure calibration ( ± 14 GPa). Comparisons between the SESAME 2140 and 2145 EOS models yielded smaller discrepancies than those attributable to uncertainty in the ablator pressure. A complete summary of the pressures determined for all data points is provided in the Supplementary Data 1 and 2.

## Supplementary information

Supplementary information

Description of Additional Supplementary File

Supplementary Data 1

Supplementary Data 2

Supplementary Data 3

Supplementary Data 4

Transparent Peer Review file

## Data Availability

The raw VISAR and X-ray diffraction data generated in this study have been deposited in the European XFEL Metadata Catalogue database under 10.22003/XFEL.EU-DATA-006746-00^71^. Data will be accessible from 19/11/2027 and can be accessed before upon request to the corresponding author. The structure factors and pair distribution functions generated in this study, along with their pressure and temperature conditions are provided in the Supplementary data files.
