# Supplementary material for: Structural evolution of iron oxides melts at Earth’s outer-core pressures
Source: Nat Commun. 2026 Jul 10;17:8527. doi: 10.1038/s41467-026-75204-4 (PMC13482581; doi:10.1038/s41467-026-75204-4)
Supplement: Supplementary file 1 [file 41467_2026_75204_MOESM1_ESM.pdf]

Supplementary Information: Structural evolution of iron oxides melts at Earth's outer-core pressure

Céline Crépisson,[1,*] Mila Fitzgerald,[1] Domenic Peake,[1] Patrick G. Heighway,[1] Thomas Stevens,[1] Adrien Descamps,[2] David McGonegle,[3] Alexis Amouretti,[4] Karim Alaa El-Din,[1] Michal Andrzejewski,[5] Sam Azadi,[1,6] Erik Brambrink,[5] Carolina Camarda,[5] David Alexander Chin,[7] Samuele Di Dio Cafiso,[8] Ana Coutinho Dutra,[1] Hauke Höppner,[8] Kohdai Yamamoto,[4] Phani S. Karamched,[9] Zuzana Konôpková,[5] Motoaki Nakatsutsumi,[5] Norimasa Ozaki,[4,10] Danae N. Polsin,[7] Jan-Patrick Schwinkendorf,[8] Georgiy Shoulga,[8] Cornelius Strohm,[5] Minxue Tang,[5] Harry Taylor,[1] Monika Toncian,[8] Yizhen Wang,[1] Jin Yao,[11] Gianluca Gregori,[1] Justin S. Wark,[1] Karen Appel,[5] Marion Harmand,[12] and Sam M. Vinko[1]

[1]*Department of Physics, Clarendon Laboratory, University of Oxford, Parks Road, Oxford OX1 3PU, UK*
[2]*School of Mathematics and Physics, Queen's University Belfast, University Road, Belfast BT7 1NN, United Kingdom*
[3]*AWE, Aldermaston, Reading, RG7 4PR, United Kingdom*
[4]*Graduate School of Engineering, University of Osaka, Suita, Osaka 565-0871, Japan*
[5]*European XFEL, Holzkoppel 4, 22869 Schenefeld, Germany*
[6]*Department of Physics and Astronomy, University of Manchester, Oxford Road, Manchester M13 9PL, United Kingdom*
[7]*University of Rochester Laboratory for Laser Energetics, Rochester, NY, USA*
[8]*Helmholtz-Zentrum Dresden-Rossendorf (HZDR), Bautzner Landstraße 400, Dresden, 01328, Germany*
[9]*Department of Materials, University of Oxford, Parks Road, Oxford OX1 3PH, UK*
[10]*Institute of Laser Engineering, University of Osaka, Suita, Osaka 565-0871, Japan*
[11]*National Thin Film Cluster Facility for Advanced Functional Materials, Department of Physics, University of Oxford, Parks Road, Oxford OX1 3PU, UK*
[12]*PIMM, Arts et Metiers Institute of Technology, CNRS, Cnam, HESAM University, 151 boulevard de l'Hôpital, 75013 Paris, France*

(Dated: June 11, 2026)

I. TARGETS CHARACTERIZATION

Iron oxides were deposited via plasma sputtering using Oxygen and Argon flux with Fe sputtering target for Fe + 4.5 FeO and well crystallized Fe_2O_3, and Fe_2O_3 sputtering target for Fe_2O_3 poorly crystallized. Deposition was performed on black Kapton, taped onto microscope glass slides. Poorly crystallized Fe_2O_3 was deposited at the National Thin Film Facility for Advanced Functional Materials (NTCF), hosted by the Department of Physics at the University of Oxford.

Scanning Electron Microscope (SEM) imaging was performed at the Physics department, University of Oxford, on all the samples with the help of J. Brown. We can see typical columnar texture for both Fe_2O_3 samples in Fig. S2 while it is less visible for Fe + 4.5 FeO sample. Samples prove to be dense and homogeneous.

X-ray diffraction (XRD) measurements were performed on the full deposited microscope slide using Bragg-Brentano diffractometer with a Co source (6.92 keV) at the Earth Sciences department, University of Oxford, with the help of K. Sokol. In Fig. S1, we compare the Bragg-Brentano measurements with the one acquired on ambient sample with the present X-ray Free-Electron Laser (XFEL) set-up described in Fig. 2 of the main paper (24 keV). Columnar texture of both Fe_2O_3 samples implies differences in relative peak intensities depending on the orientation of the grains probed, which explains the difference between the two types of measurements observed for the two Fe_2O_3 samples in Fig. S1. For Fe + 4.5 FeO samples we can see that FeO related peaks have similar intensity between the two measurement while Fe body centred cubic (bcc) peaks intensity do vary in link with Fe texture. While both Fe_2O_3 samples are presenting Fe_2O_3 Bragg peaks, we can see that one (in red in Fig. S1) has broader peaks, indicating that it is poorly crystallized.

Electron MicroProbe Analyses (EMPA) were performed at the Earth Science department, University of Oxford with the help of A. Matzen. Fe_2O_3 and Fe + 4.5 FeO samples were mounted in resin and polish to expose a section, as shown in Fig. S3. Measurements were then performed along three lines across around 2 cm of exposed samples, either

* Corresponding author. Email: celine.crepisson@physics.ox.ac.uk

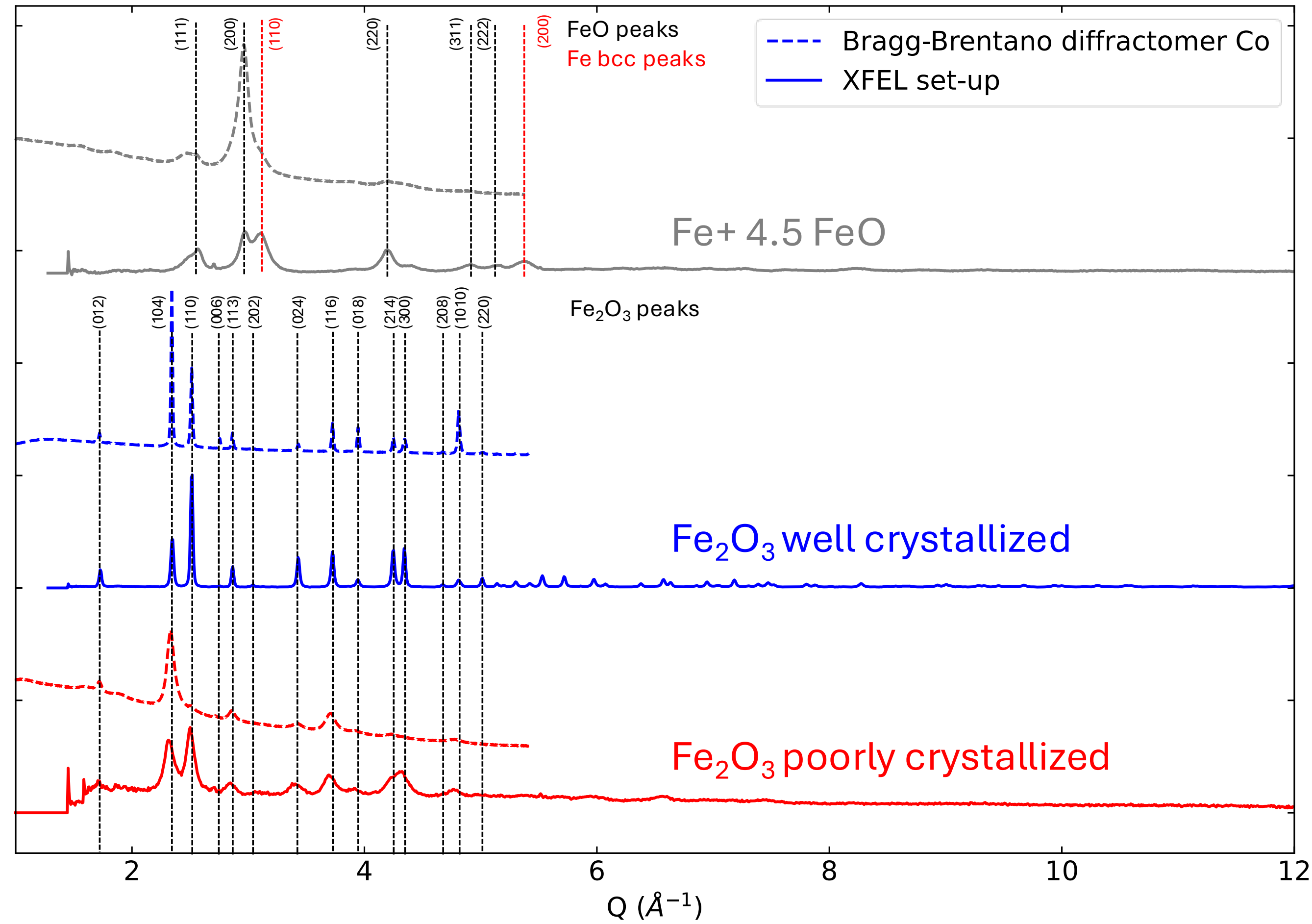

Figure S1. X-ray diffraction of a full slide of deposited sample (76 x 26 mm) using Bragg-Brentano diffractometer (reflection geometry with deposited iron oxide facing the x-ray) with Co source in dashed line compared to ambient measurement with the present XFEL set-up in plain line. Differences in relative peak intensity are mostly linked to the texture of the sample which implies a change in peak intensity depending of the orientation of the grains probed. Vertical dashed lines indicate Bragg peaks indexation for alpha-Fe_2O_3 [1], FeO rocksalt structure and Fe bcc.

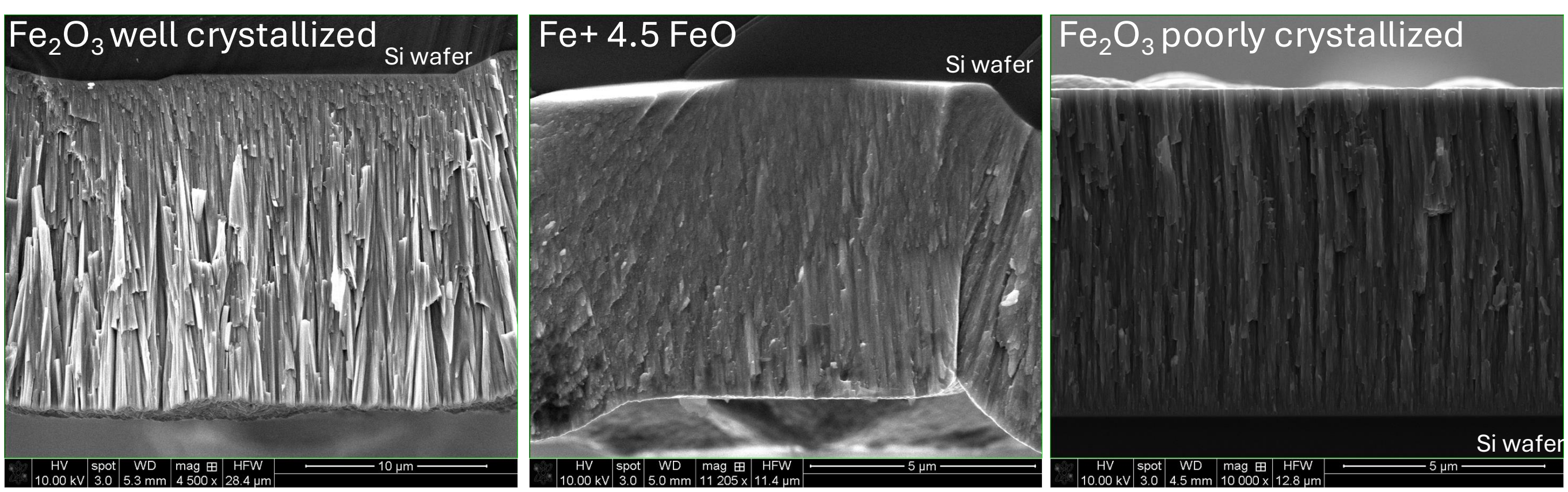

Figure S2. Scanning electron imaging (Secondary electron image) of a thin section (section was manual and is not perfectly perpendicular to the deposition plane) of the different samples deposited on silicon (Si) wafer. Position of the Si wafer is indicated. We can see that grain size is smaller on the substrate side. Working distance (WD), voltage (HV), spot size (size) (3 µm) and magnification (mag) used for the imaging are reported at the bottom of the image.

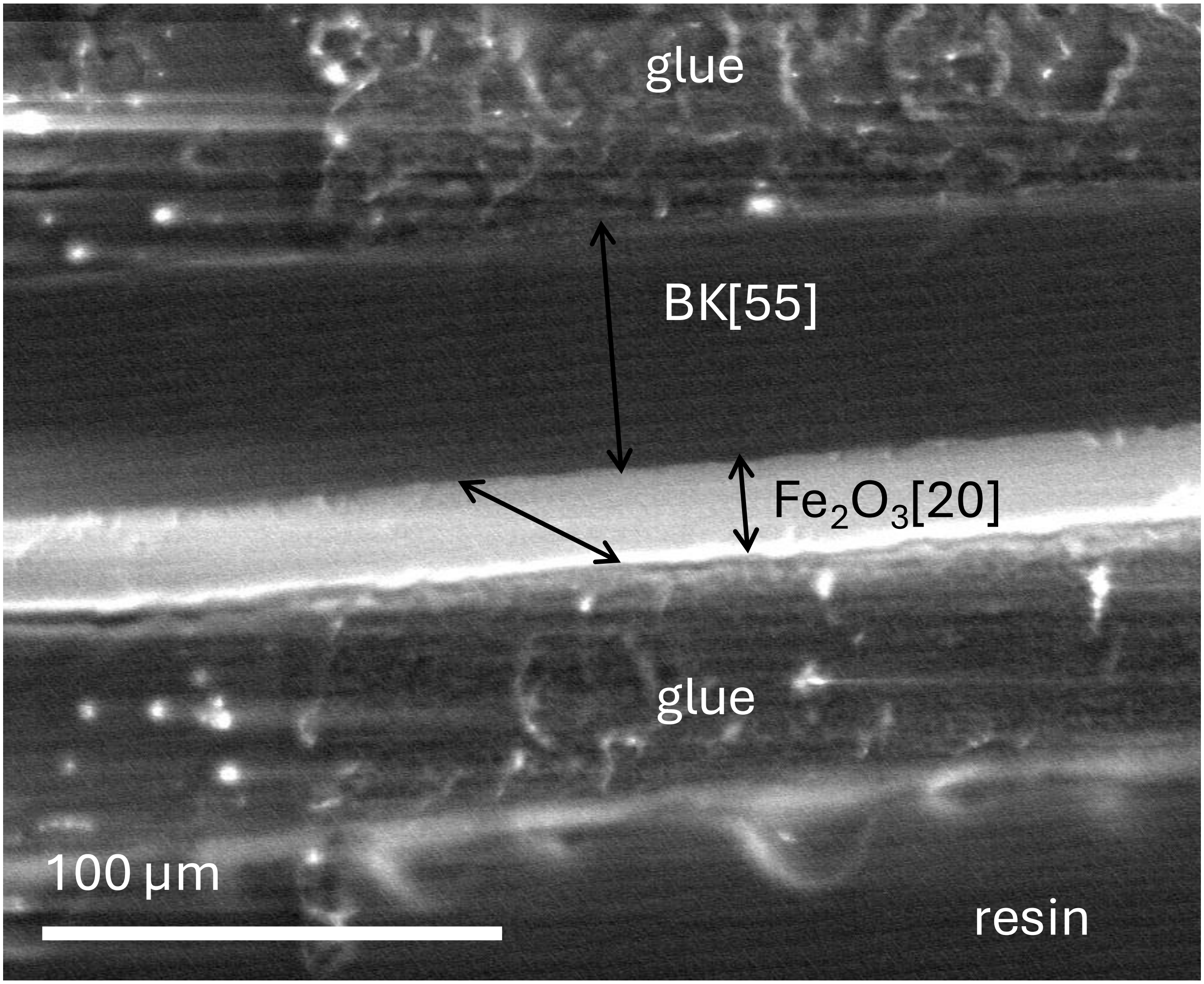

Figure S3. Secondary electron image at the electron microprobe showing section exposed and analyzed. Example of straight and diagonal lines are shown. Sample is glued on its thickness on a cover glass, mounted in resin and polished to exposed the iron oxide sample so that neither glue nor glass is present on top of the sample. Sample is polished down to 0.25 µm using diamond paste.

straight or diagonal (around 45 degrees angle). Fe, Mg and O were measured using respectively fayalite, hematite and MgO standards. Magnetite standards were analysed proving the quality of the analysis for iron oxides. Fe_2O_3 samples proved homogeneous in section and across the sample with a composition in atomic percent of 40.4(0.2)at% Fe and 59.6(0.2)at% O. Fe + 4.5 FeO samples also proved homogeneous in section and across the sample with a composition in atomic percent of 55.0(0.4)at% Fe and 45.0(0.4)at% O, which indicates a Fe + 4.5 FeO composition (assuming stoichiometric FeO). EMPA analyses are given in Supplementary Data 3.

The exact $Fe_{1-x}O$ stoichiometry could not be determined because bcc-Fe and wüstite grains were intermingled at sub-micron length scales, smaller than the EMPA interaction volume. The $Fe_{1-x}O$ sample is also showing an intrinsic residual stress from thin layer sputtering due to PVD. From preshot measurements and regions remaining at ambient conditions during driven shots, the initial molar volume was determined to be $V_0 = 11.61(0.03)$ $cm^3.mol^{-1}$. Owing to residual stress, the $Fe_{1-x}O$ lattice parameter cannot be used to infer stoichiometry via the relation of McCammon and Liu [2]. Nevertheless above 5 GPa, FeO is expected to be close to stoichiometric in presence of Fe excess [3, 4].

$Fe_{1-x}O$ is known to exist in composition ranging from $Fe_{0.88}O$ to $Fe_{0.96}O$ at ambient conditions [5]. Our sample is composed of Fe and $Fe_{1-x}O$. Volumes are determined by the position of the Bragg peak from XRD measurement. V_0 Fe is 7.148(0.05) $cm^3 \cdot mol^{-1}$ (average of 24 data points from preshot measurement or remaining ambient under shock

not all presented in this work but to be submitted separately) and V_0 $Fe_{1-x}O$ of 11.614(0.03) $cm^3 \cdot mol^{-1}$ (average of 32 data points from preshot measurement or remaining ambient under shock not all presented in this work but to be submitted separately). The initial volume found for $Fe_{1-x}O$ corresponds to a pressure of 8-11 GPa if we assume a bulk modulus of 141.5 or 185 GPa (taking the extreme value published so far [2, 6, 7]), a FeO composition, i.e. an expected volume of 12.252 $cm^3 \cdot mol^{-1}$ at ambient conditions [2] and using second order Birch-Murnaghan EOS. Therefore, it is possible that our sample is stoichiometric FeO at ambient condition due to this initial compression as it has been shown that above 5 GPa, $Fe_{1-x}O$ in presence of Fe is stoichiometric FeO [3, 4]. Density of our sample can be calculated as follows:

$$\rho_{\text{sample}} = \frac{(AM_{\text{Fe}} + BM_{\text{Fe}_{1-x}\text{O}})}{(AV_{\text{Fe}} + BV_{\text{Fe}_{1-x}\text{O}})} \tag{S1}$$

with A and B the atomic proportions of Fe and $Fe_{1-x}O$ in the sample and M_{Fe} and $M_{Fe_{1-x}O}$ the average molar mass and V_{Fe} and $V_{Fe_{1-x}O}$ the measured volume in $cm^3.mol^{-1}$ detailed above. We know the Fe and O contents based on EMPA analysis (55.0(0.4) at% Fe and 45.0(0.4) at% O: depending on the stoichiometry we can find A and B the proportion of Fe and $Fe_{1-x}O$. If we assume a stoichiometric FeO, we have $A = 1$, and $B = 4.5$, while if we assume $Fe_{0.88}O$, we have $A = 1.54$ and $B = 4.5$. Thus density could at the maximum vary from 6.00 $g.cm^{-3}$ for $Fe_{0.88}O$ composition to 6.38 $g.cm^{-3}$ for stoichiometric FeO. For the highest pressure calculated (440(50) GPa) using an initial density of 6.38 $g.cm^{-3}$, if the density is changed to 6.00 $g.cm^{-3}$, this decreases the estimated pressure by around 30 GPa, while it only decreases the pressure by 10 GPa at 200(20) GPa. We thus note that the effect of the initial density on pressure estimate remains within previously determined error bars.

A thin section of the Fe + 4.5 FeO sample was prepared (from the substrate to the free surface of the deposition) using a Zeiss Auriga dual beam Focussed Ion Beam (FIB) - SEM. The prepared lamella was thinned to sub-100 nm thickness and observed using a JEOL GrandARM 300F available at the David Cockayne Centre for Electron Microscopy at the Department of Materials, University of Oxford. The instrument was operated in Scanning Transmission Electron Microscope (STEM) mode at 300 KV with a Cold Field Emission Gun, capable of atomic resolution. We observed grain size below 10 nm throughout the sample as shown in Fig. S4 and Fig. S5.

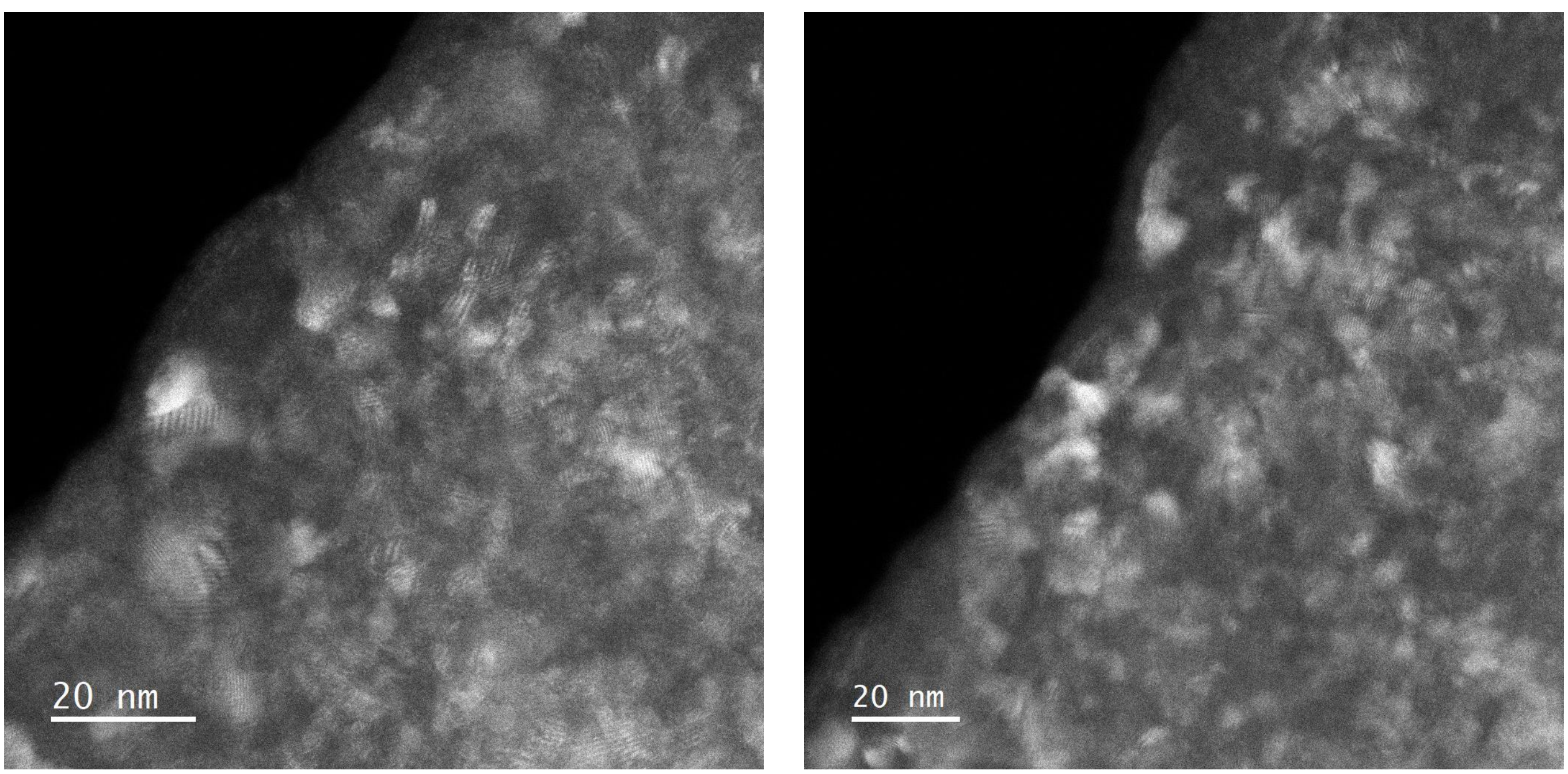

Figure S4. Annual Dark Field STEM images recorded in 2 positions on a FIB section of the initial Fe + 4.5 FeO sample. The edge shows the exposed sample surface and the substrate is towards the bottom right direction in the image.

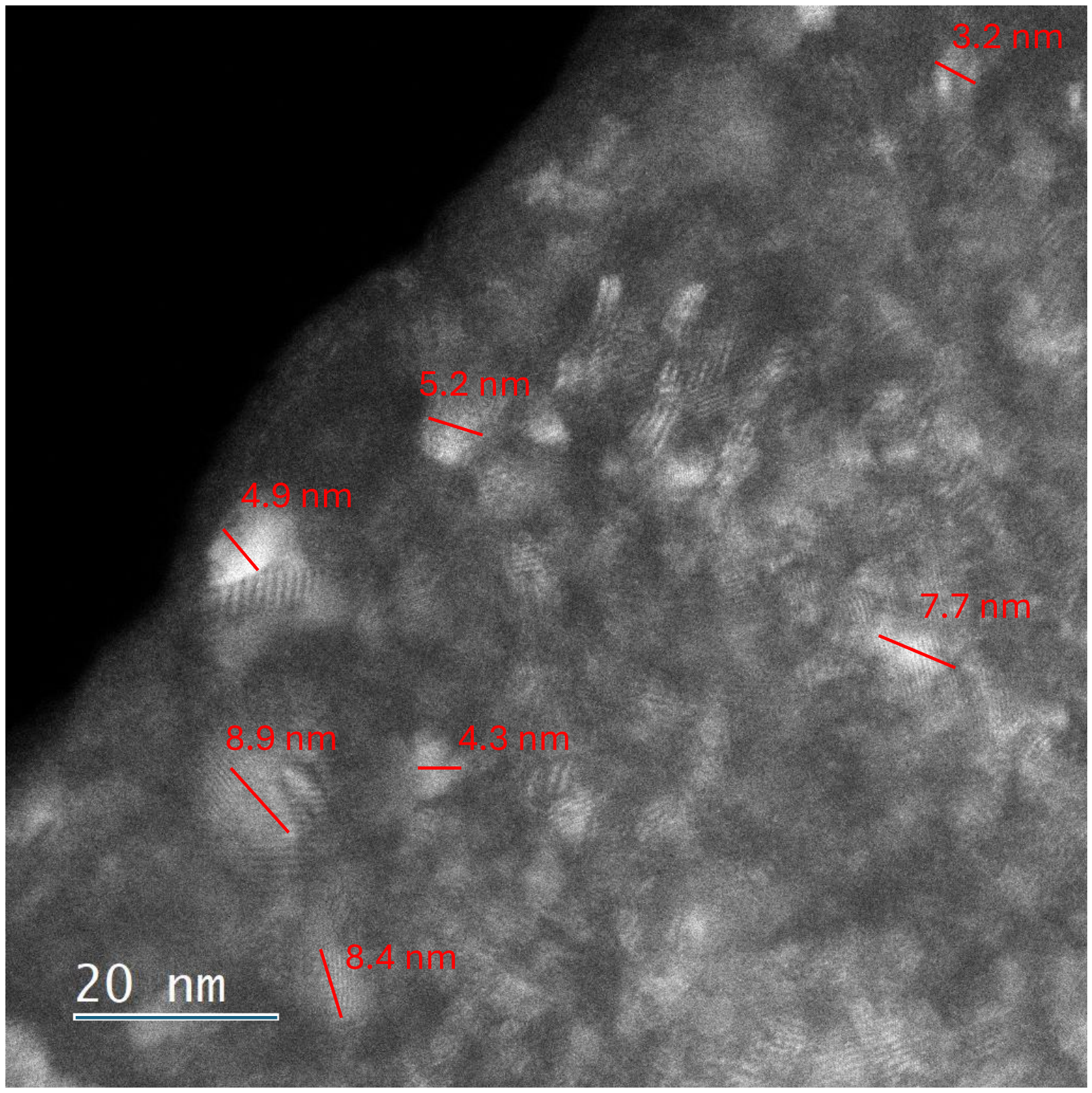

Figure S5. Few sizes of grain are shown on the annual Dark Field STEM image shown on Fig. S4 left.

II. PRESSURE CALIBRATION CURVE

Laser shots were performed on isolated black Kapton samples (77 µm black Kapton with 200 nm Al coated on the VISAR side) to determine transit time via VISAR at a given laser intensity. Transit time was determined with great accuracy (around 25 ps) using the fiducial position [8]. Pressure was then calculated using polyimide equation of state and thickness of black Kapton (measured to 76.6 to 77.3 µm using touch probe) and results are shown in Fig. S6. Errors arise first from uncertainty in black Kapton thickness and polyimide equation of state [9]: at the maximum ±6 GPa. Main cause of error comes from the interpolation of the data using a power law curve which can reach 10 GPa. Combination of uncertainties can reach at the maximum ±14 GPa which is used as an error estimate when using the power law calculated here to infer pressure in black Kapton at a given laser intensity. This pressure calibration curve was used in this work to determine pressure via impedance match method[10] in our samples. Pressure in function of laser intensity was also estimated by using 20 µm Cu foil glued on top of 55 µm black Kapton ablator. Cu volume under shock was estimated based independently on copper faced-centre cubic Bragg peak (200) and (111). Difference in volume based on those two diffraction peaks was used to estimate the error in the measurement. Pressure in Cu at a given volume was determined using SESAME 3336 similarly to Sharma et al. 2020 [11]. Pressure in the black Kapton ablator was determined by using the impedance match method: SESAME 7770 was used for black Kapton and SESAME 3336 for Cu. We compare our results with a previous study using similar experimental set-up (Gorman et al., 2024) [12] after recalculation of the pressure in the black Kapton ablator using full equation of state. Although pressure in black Kapton ablator in function of laser intensity is slightly lower when based on transit time in black Kapton compared to determination from Cu XRD volume, they are in relative agreement and coherent with previous experiment with similar set-up [12].

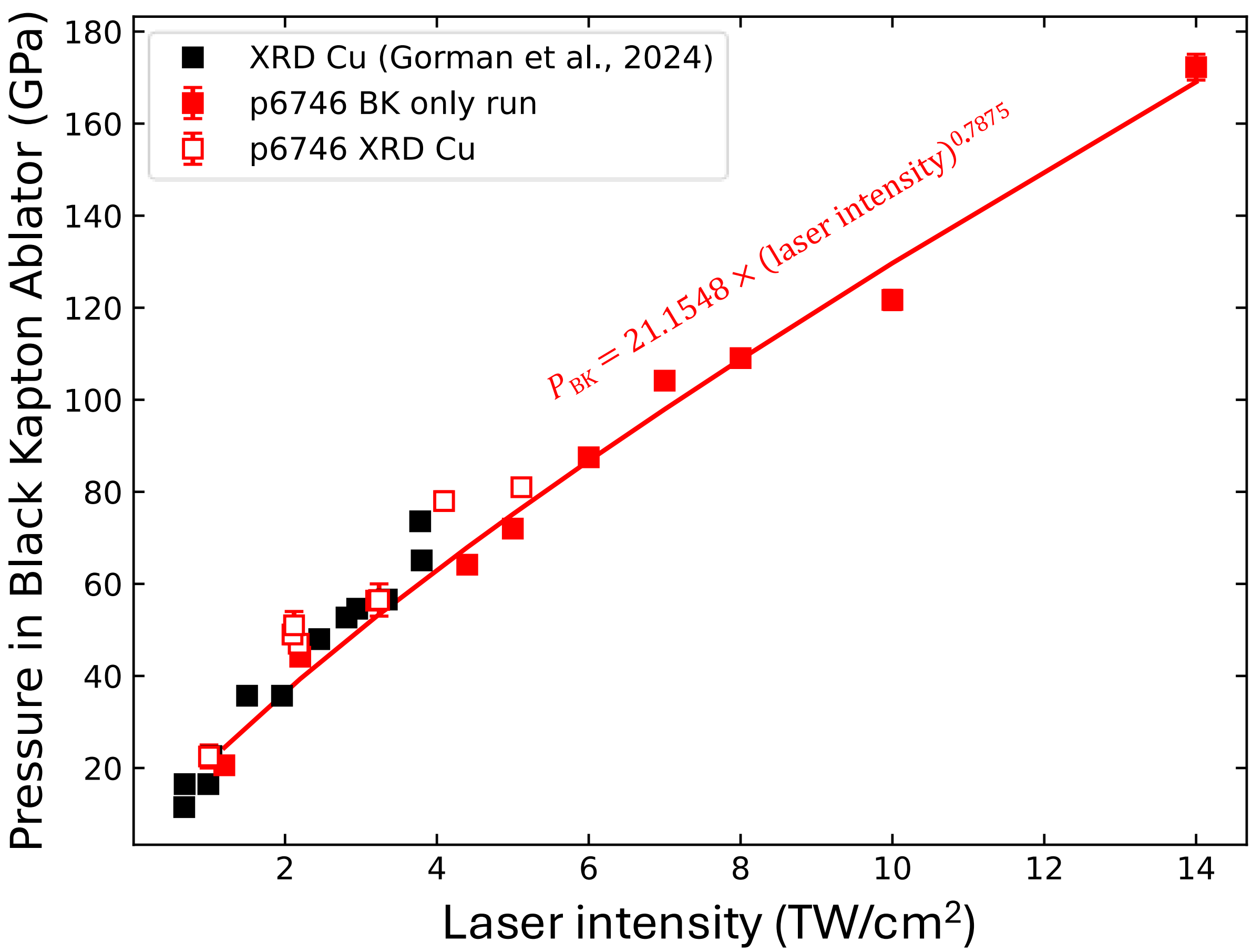

Figure S6. Pressure in black Kapton ablator based on transit time measured by VISAR on 77 µm black Kapton sample with 200 nm Al coating on VISAR side. Comparison with pressure in black Kapton inferred from Cu volume determined from XRD and using the impedance match method.

III. HYDRODYNAMIC SIMULATIONS

Hydrodynamic simulations using the code MULTI [13] were performed to determine pressure in Fe + 4.5 FeO and Fe_2O_3 samples based on the transit time measured by VISAR. We use SESAME 7770 for black Kapton, SESAME 7440 [14] for Fe_2O_3 and as an approximation we use FeO Hugoniot relation from Jeanloz and Ahrens, 1980 [7] for Fe + 4.5 FeO sample. We give example for 10 ns, 6 ns and 4 ns pulse durations in Fig. S7. We can see that for 6 and 4 ns pulse durations for Fe_2O_3 sample which is relatively thick (20 µm) the sample is not in an homogeneous state when probed and we thus use hydrodynamic simulations to determine the range of pressures in the probed sample at the given probed time. Results of all hydrodynamic simulations are reported in Supplementary Data 1. For Fe no hydrodynamic simulation was performed as transit time is not reliable due to variation in the glue layer.

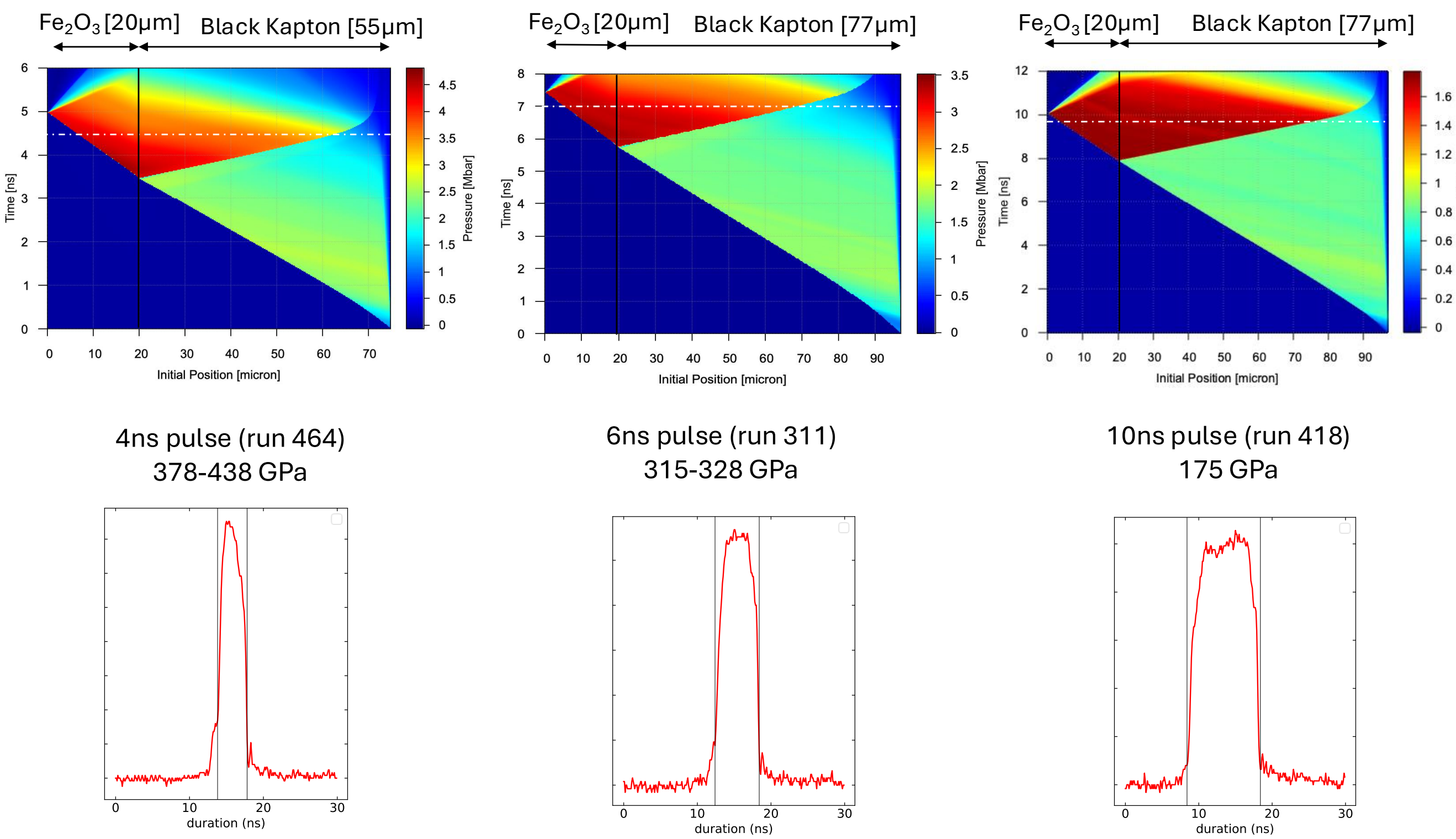

Figure S7. Hydrodynamic simulations in Fe_2O_3 matching the transit time measured by VISAR as shown in Supplementary Data 1. White dashed line indicated the time at which the sample is probed. Laser pulse is arriving from the right. Real pulse profiles are shown at the bottom: vertical black line delimit the actual pulse profile used in the hydrodynamic simulations.

As Fe + 4.5 FeO sample has a different initial density (ρ_{sample}) than EOS initial density used in the hydrodynamic simulation (see details in section I) and for impedance match (ρ_{eos}) we correct for this effect afterward following Rankine-Hugoniot equations:

$$P = \rho_0 U_{\text{s}} U_{\text{p}} \tag{S2}$$

Thus:

$$P = P_{\text{hydro}} \frac{\rho_{\text{sample}}}{\rho_{\text{eos}}} \tag{S3}$$

IV. RENORMALIZATION OF THE FLUX USING THE DIODE

OPT IPM diode present in the chamber [15] were used to renormalize the data to correct for XFEL beam flux variations. We can see in Fig. S8 that the OPT IPM diode channel reading is well correlated with the XRD 2D image

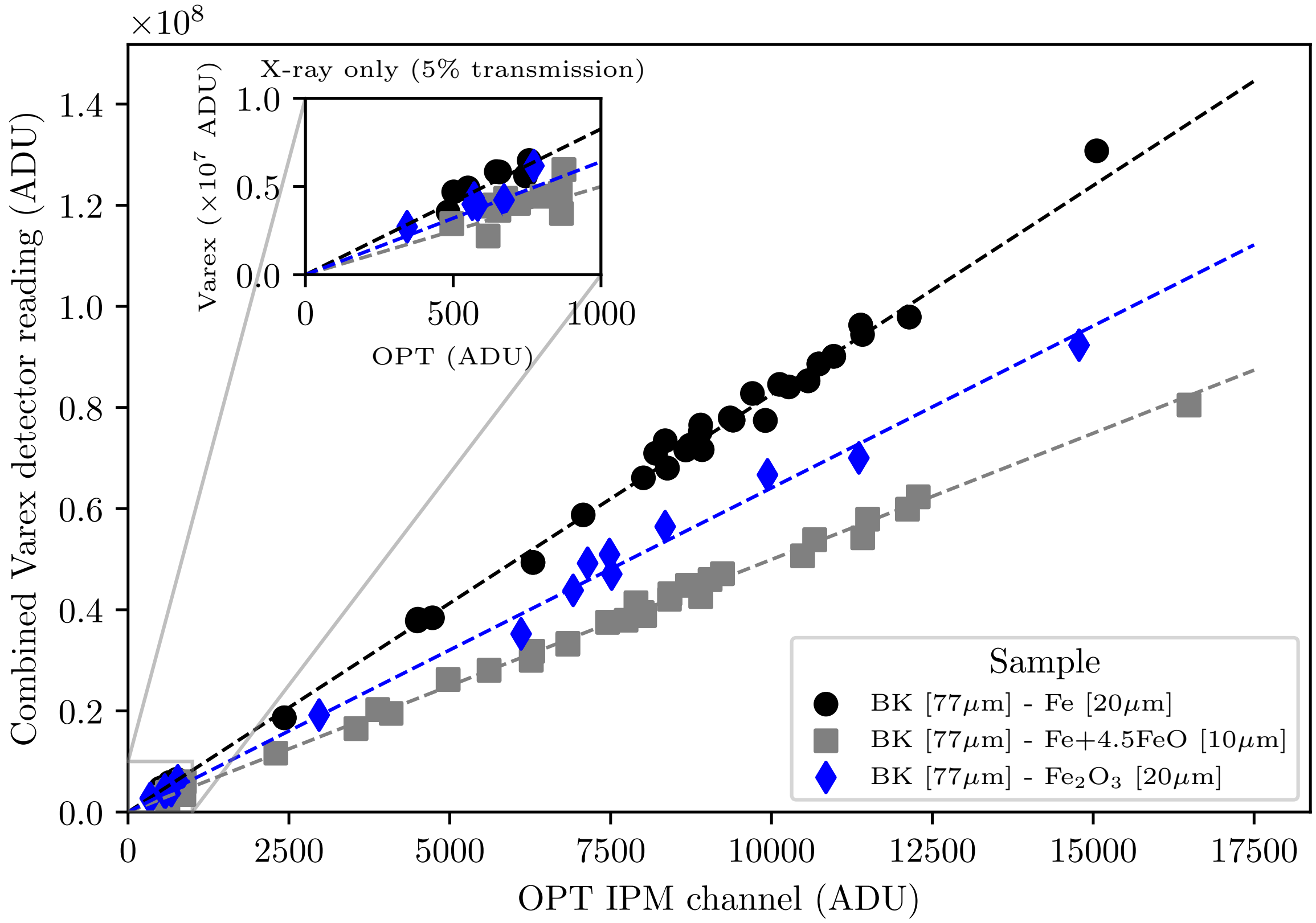

Figure S8. OPT IPM channel reading in function of the combined Varex detector reading in analog-to-digital units (ADU). OPT IPM diode can be reliably used to renormalize for x-ray flux variation across the experiment for all targets and for both ambient measurements before the shot (5% transmission) and shots.

intensity represented here by the combined Varex detector reading.

V. CONTRIBUTION OF THE ABLATOR TO THE XRD SIGNAL OF LIQUID

XRD signal of the black Kapton under shock displayed in Fig. S9 is varying significantly between ambient black Kapton and compressed black Kapton from 21(1) GPa. No further change is identified from 21(1) up to 122(4) GPa under shock. This result is in agreement with previous laser and explosives studies [16] which evidenced a volume discontinuity along the Hugoniot around 26 GPa and XRD under laser-driven shock study which determined a melting pressure of 32(3) GPa [9]. Small pressure differences are likely to be linked to the type of polyimide used in the different studies. Run slightly in release (up to 0.2 ns) are also very similar to fully compressed one.

In Fig. S7 we can see that for data point at 175 GPa, which is the lowest pressure at which we performed liquid diffraction analysis, black Kapton under shock is at 84 GPa (prior to entry of the shock in the Fe_2O_3 layer) while after entry of the shock in the Fe_2O_3 layer, black Kapton experiences a reshock leading to a pressure of 170 GPa. Upon reshock the black Kapton has a lower temperature compared to a single shock. Indeed, based on hydrodynamic simulation we found a temperature of 1.2 eV for black Kapton under shock at 170 GPa while a temperature of 0.5-0.6 eV is observed upon reshock with an adjacent Fe_2O_3 layer as seen in Fig. S7. We also see in Fig. S7 that black kapton state is not homogeneous at probed time (indicated by a white dashed line). In this work, we assume that black Kapton XRD signal will not significantly change upon shock and reshock above 170 GPa. As there is so far no clear black Kapton melting curve, this hypothesis will however need to be further confirmed. Within this approximation, we thus use data point at 122(4) GPa to represent black Kapton contribution, being the highest pressure available in our dataset, at which black Kapton is entirely compressed. We use a smooth lineout of this data point as shown in Fig. S10 to avoid adding noise to melt signal of interest. For ambient black Kapton, we use a structureless black Kapton model whose high scattering angle contribution was recalibrated using the data point at 21(1) GPa as no isolated ambient black Kapton was measured during the experiment.

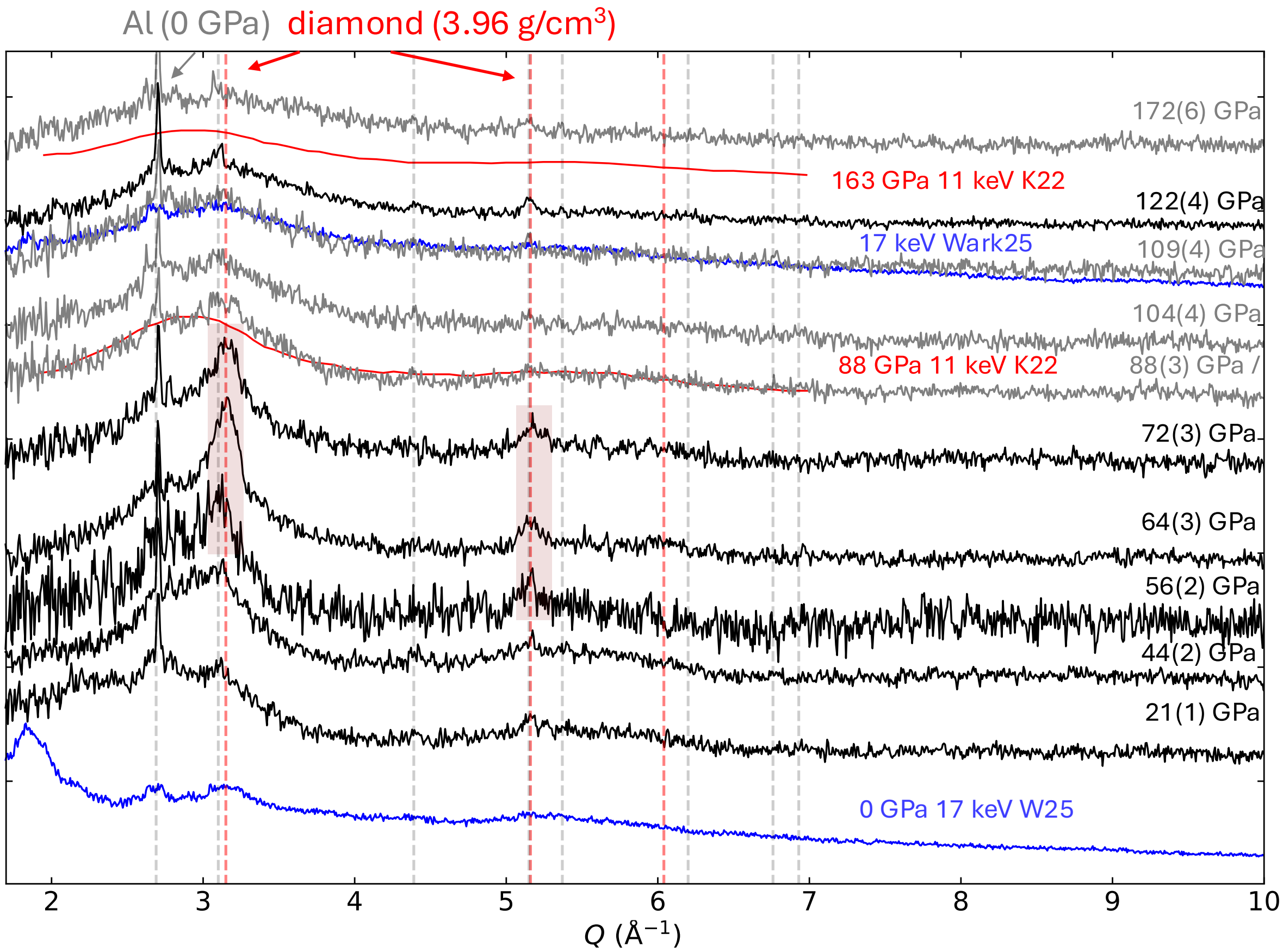

Figure S9. Azimuthally integrated 1D lineout for isolated black Kapton (77 µm) sample with 200 nm Al coating on top under shock. Data in black are probed before breakout time, i.e., the moment when the shock leaves the black Kapton layer, while data in grey are probed slightly after breakout time (up to 0.2 ns) as detailed in Supplementary Data 1. Data are compared with previous XRD data of black Kapton under shock (fully corrected) using the same data treatment regarding XRD correction and the same experimental set-up but at 17 keV (Wark25 [17]). We can see a change of XRD intensity at high angle in link with the Compton scattering which is slightly different at different x-ray energy, although the signal under shock is overall the same. We also compare our data to polyimide under shock measured at 11 keV (K22 [9]) which also prove to be similar. We thus use data point at 122(4) GPa to represent black Kapton contribution as it is the highest data point with relatively good signal over noise ratio. Al Bragg peaks, in link with Al coating on top of the black Kapton are indicated in vertical dashed light grey lines, and diamond peak at 3.96 g/cm^3 are indicated in vertical red dashed lines.

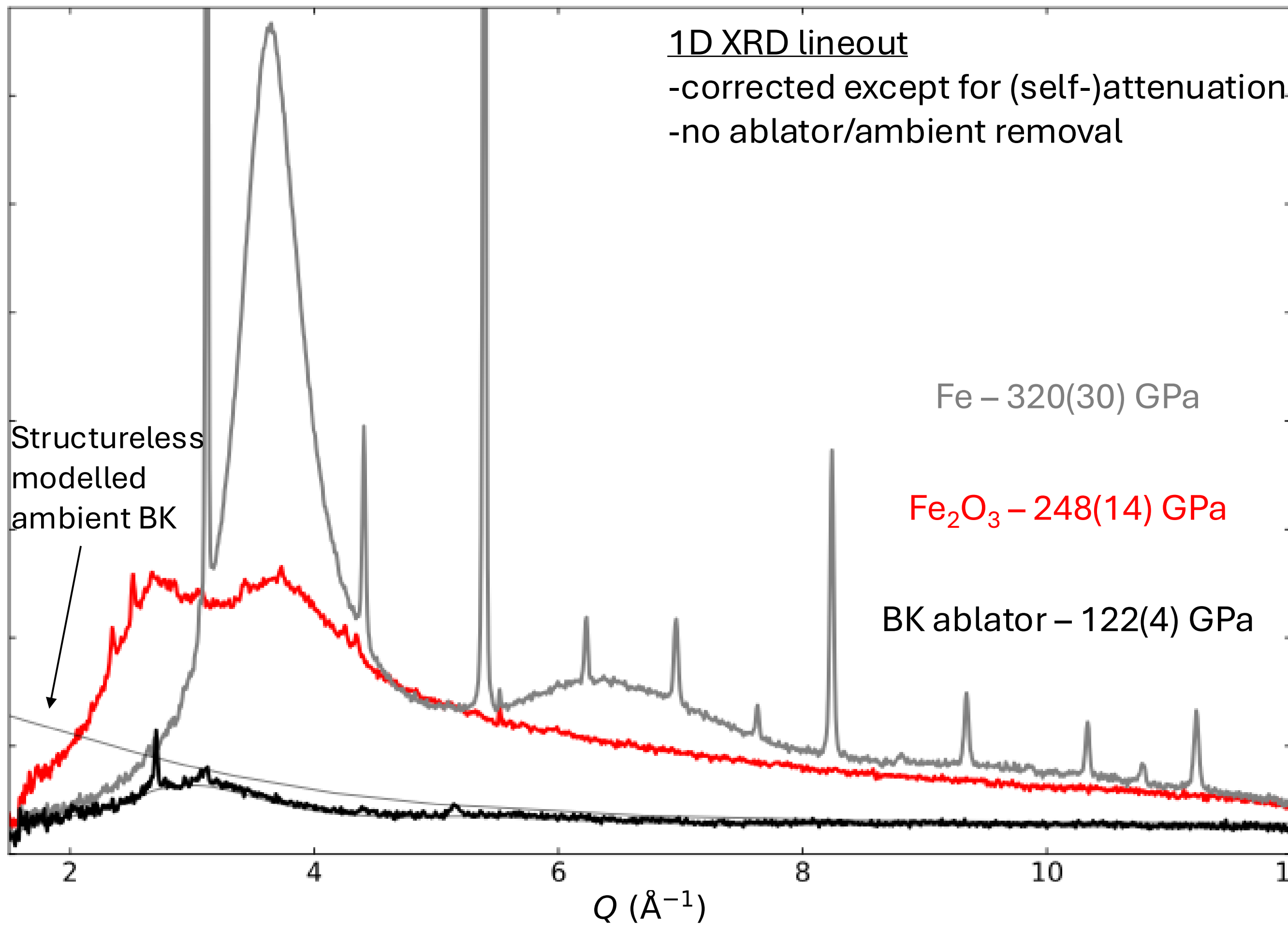

Figure S10. Azimuthally integrated XRD 1D lineout showing relative intensity of black Kapton under shock, Fe and Fe_2O_3 melt under shock. We can see that black Kapton ablator contribution is significant under shock for Fe_2O_3 melt.

VI. DIAMOND FORMATION IN BLACK KAPTON

Diamond peaks are observed clearly in black Kapton under shock at 56(2), 64(3) and 72(3) GPa as seen in Fig. S9. They correspond to a density of 3.96 g/cm^3 in diamond which is expected at these pressures and temperatures [18]. They lie in a high temperature region compared to diamond formation in PET [19, 20], assuming that temperature in black Kapton is well reproduced by SESAME 7770 as seen in Fig. S11. Diamond peaks are unlikely above around 90 GPa in shocked black Kapton due to high temperature, located above the diamond melting curve although it remains unclear if a diamond peak is observed in our data at 122(4) GPa as a faint peak is seen at the expected location (Fig. S9). Nonetheless, in reshocked black Kapton, temperature is lower than the Hugoniot as previously discussed. If we take the lower pressure data point for which we performed liquid diffraction analysis, i.e. 177(14) GPa in Fe_2O_3, black Kapton experiences a pressure of around 170 GPa and a temperature of around 0.5 eV (5,800 K). As seen in Fig. S11, this condition in black Kapton lies at the high pressure edge of the diamond formation field. Therefore, higher investigated pressures are unlikely to lead to diamond peak. Moreover, no diamond peak was observed in our XRD data under shock.

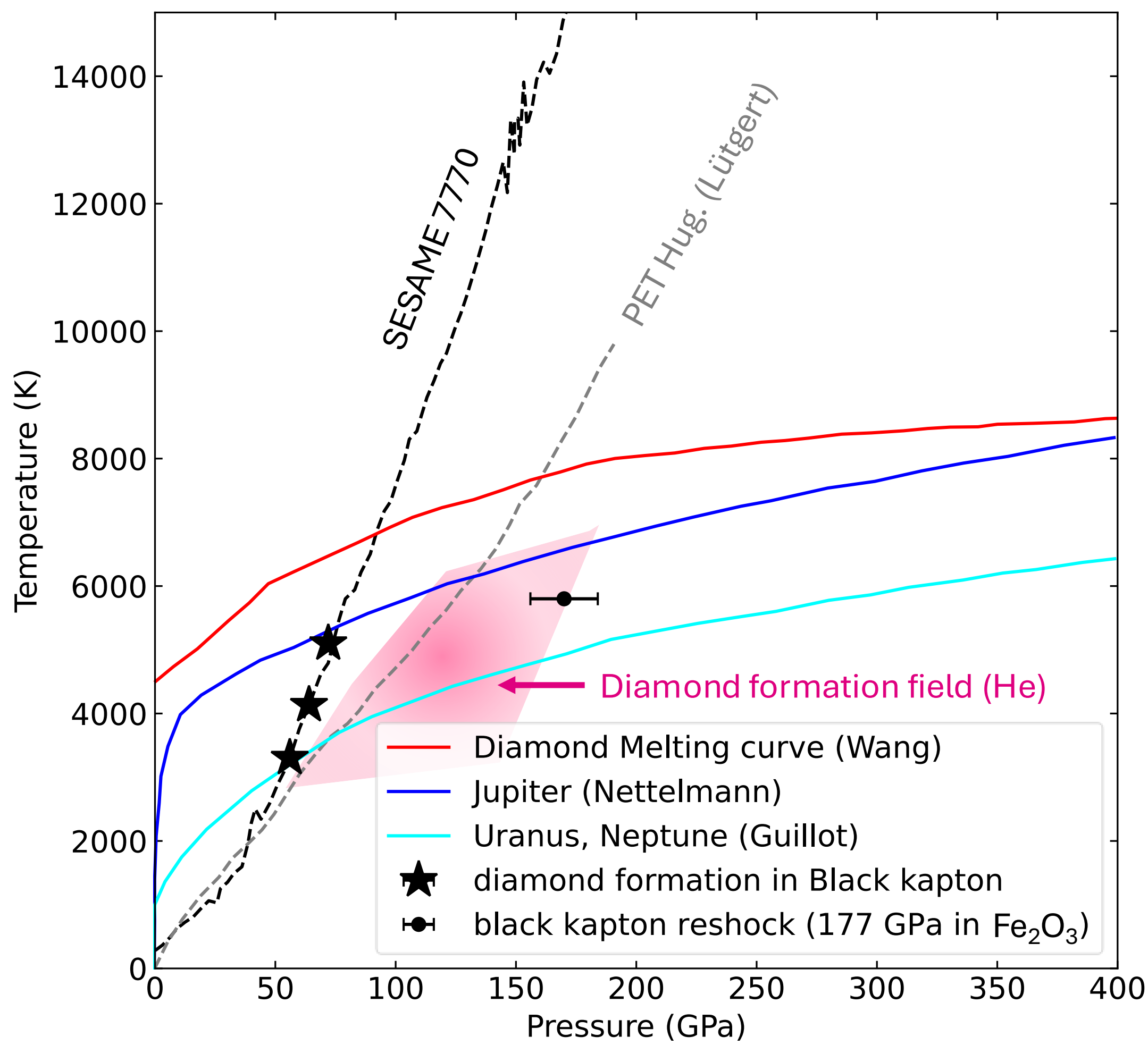

Figure S11. Figure adapted from He et al., 2022 [19] showing diamond formation field along with diamond melting curve (Wang [21]), PET Hugoniot [22] and Pressure, temperature within Jupiter (Nettelmann [23]) and Uranus and Neptune (Guillot [24]). Diamond formed in black Kapton under single shock observed here are seen at higher temperature than expected: however we make here the assumption that SESAME 7770 reproduce well black Kapton equation of state which remains hypothetical.

VII. STUDY OF SOLID FE

Fe transform from Fe bcc into Fe hexagonal close packed (hcp) structure under pressure as seen in Fig. S12. Fe hcp cell parameters are in agreement with previous laser-driven shock compression as seen in Fig. S13. We note that Fe hcp is still observed at 280(30) GPa, which is higher than previous study although within our pressure uncertainties.

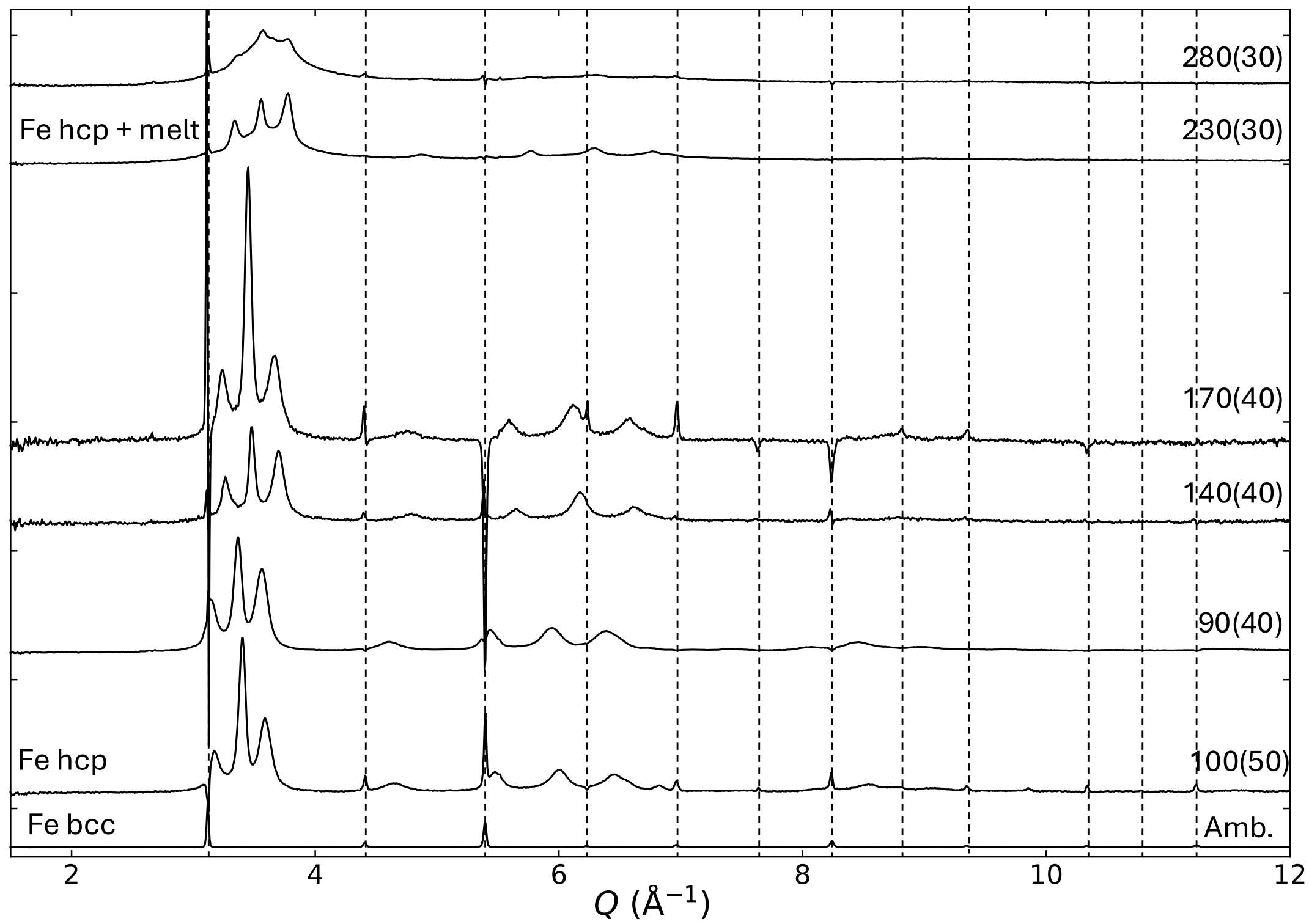

Figure S12. Azimuthally integrated XRD 1D lineout for Fe solid. Vertical dashed line indicate Fe bcc ambient Bragg peaks. We can see that relative intensity of ambient peaks are varying in link with texture of the rolled Fe foil.

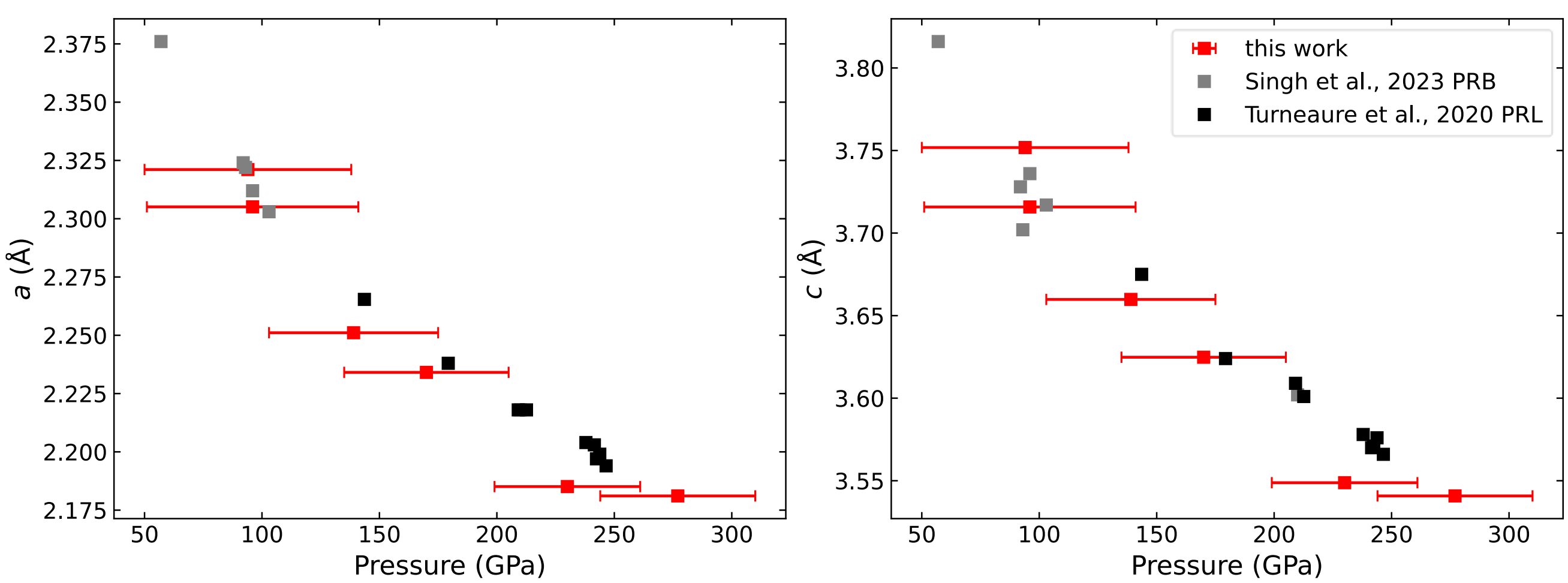

Figure S13. Fe hcp cell parameters in function of pressure (from impedance match) compared to previous studies of Fe using XRD under laser-driven shock compression: Turneaure et al., 2020 PRL [25] and Singh et al., 2023 PRB [26].

VIII. STUDY OF SOLID CRYSTALLINE FE_2O_3

We performed Le Bail refinement on the solid phases of Fe_2O_3, at ambient conditions, and under shock at 33(14) GPa and 82(14) GPa. It is not possible to perform Rietveld refinements on the data due to the complex microstructure of the samples under shock. Refinements shown in Fig. S14, Fig. S15 and Fig. S16 indicate that the reflections observed are well explained by an α to α'-Fe_2O_3 phase transition similarly to previous study of Fe_2O_3 under laser-driven shock compression [27]. None of the phase transitions reported under static compression [28] are observed. The presence of a small Al contribution is due to the approximately 200 nm coating on the target. One data point presents both α and α'-Fe_2O_3 phases: hydrodynamic simulations showing pressures of 42 and 64 GPa (Fig. S17). We report cell parameters in Supplementary Data 1. We can see that at 97(14) GPa, several α'-Fe_2O_3 Bragg peaks disappeared in link with amorphization under shock at around 108(14) GPa (Fig. S17).

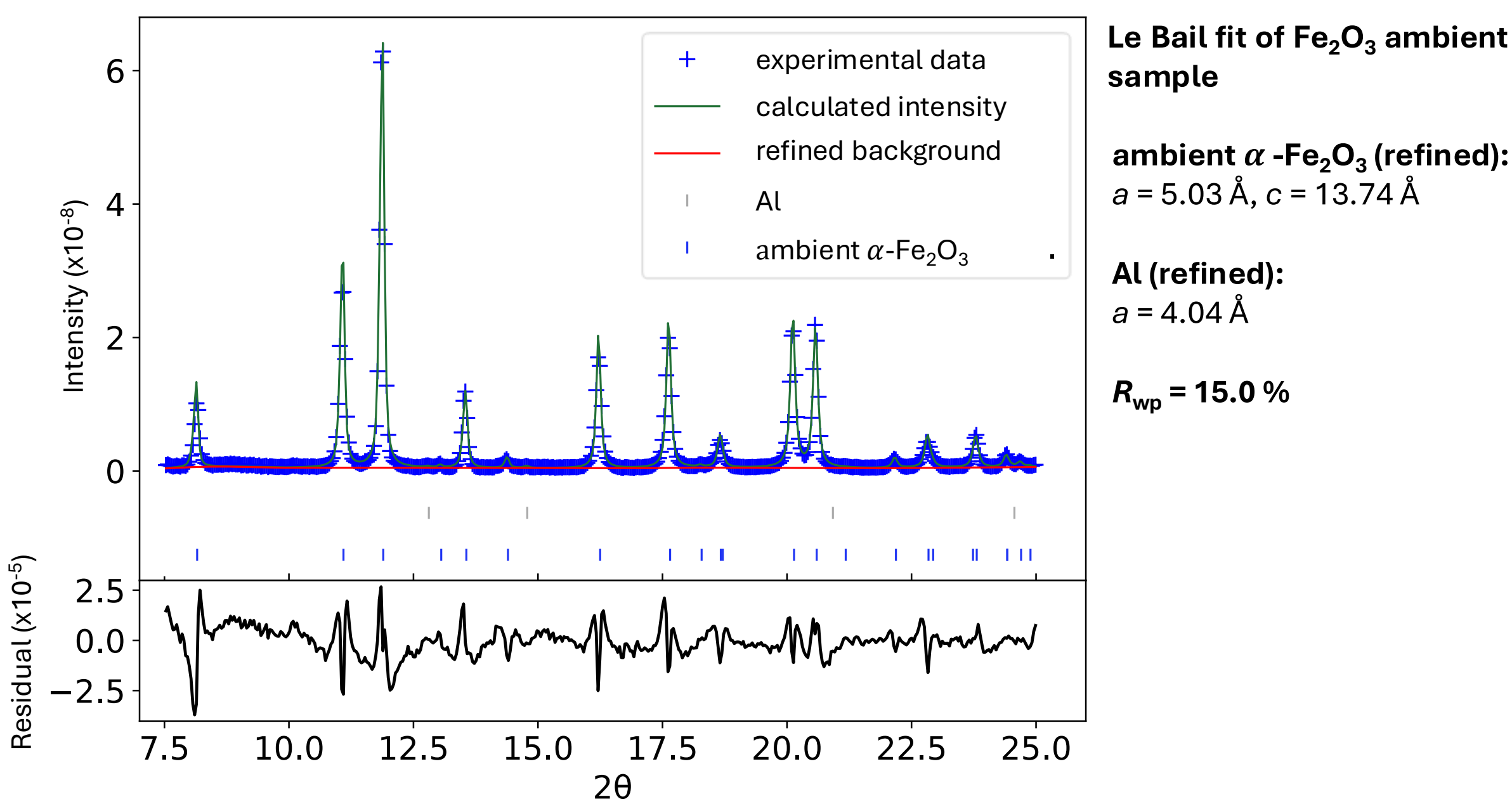

Figure S14. Le Bail refinement of ambient sample performed on azimuthally integrated 1D XRD lineout after combining the two VAREX detectors and correcting the XRD intensity, as detailed in the Methods section C of the main paper. Fitted cell parameters of α-Fe_2O_3 phase and Al are given, along with weighted profile R-factor (R_{wp}) of the fit.

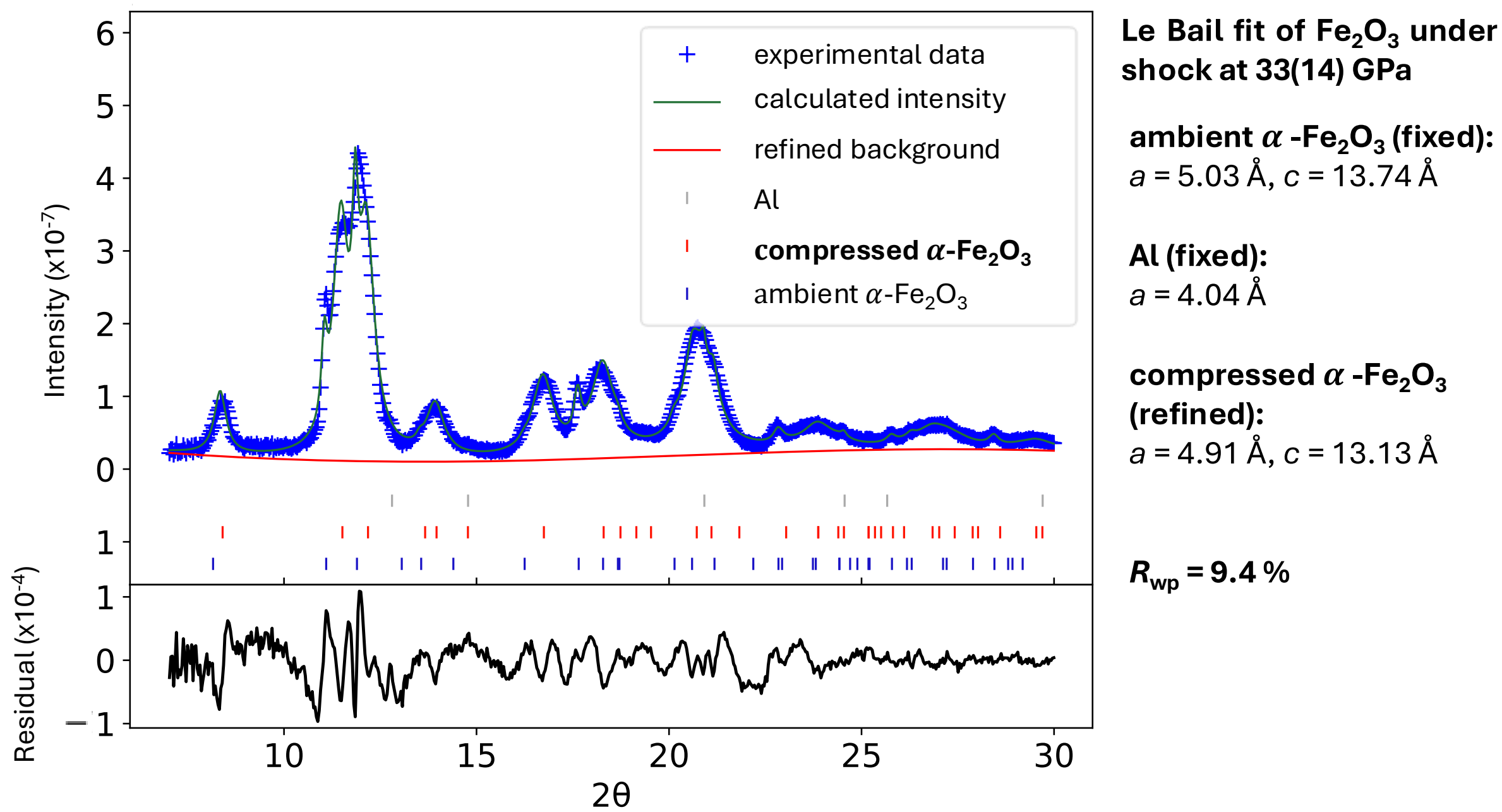

Figure S15. Le Bail refinement of shocked sample at 33(14) GPa performed on azimuthally integrated 1D XRD lineout after combining the two VAREX detectors and correcting the XRD intensity, as detailed in the Methods section C of the main paper. Cell parameters of ambient α-Fe_2O_3 phase and Al are fixed to the value found by Le Bail refinement of the ambient sample (Fig. S14). Fitted cell parameters of compressed α-Fe_2O_3 phase are given, along with weighted profile R-factor (R_{wp}) of the fit.

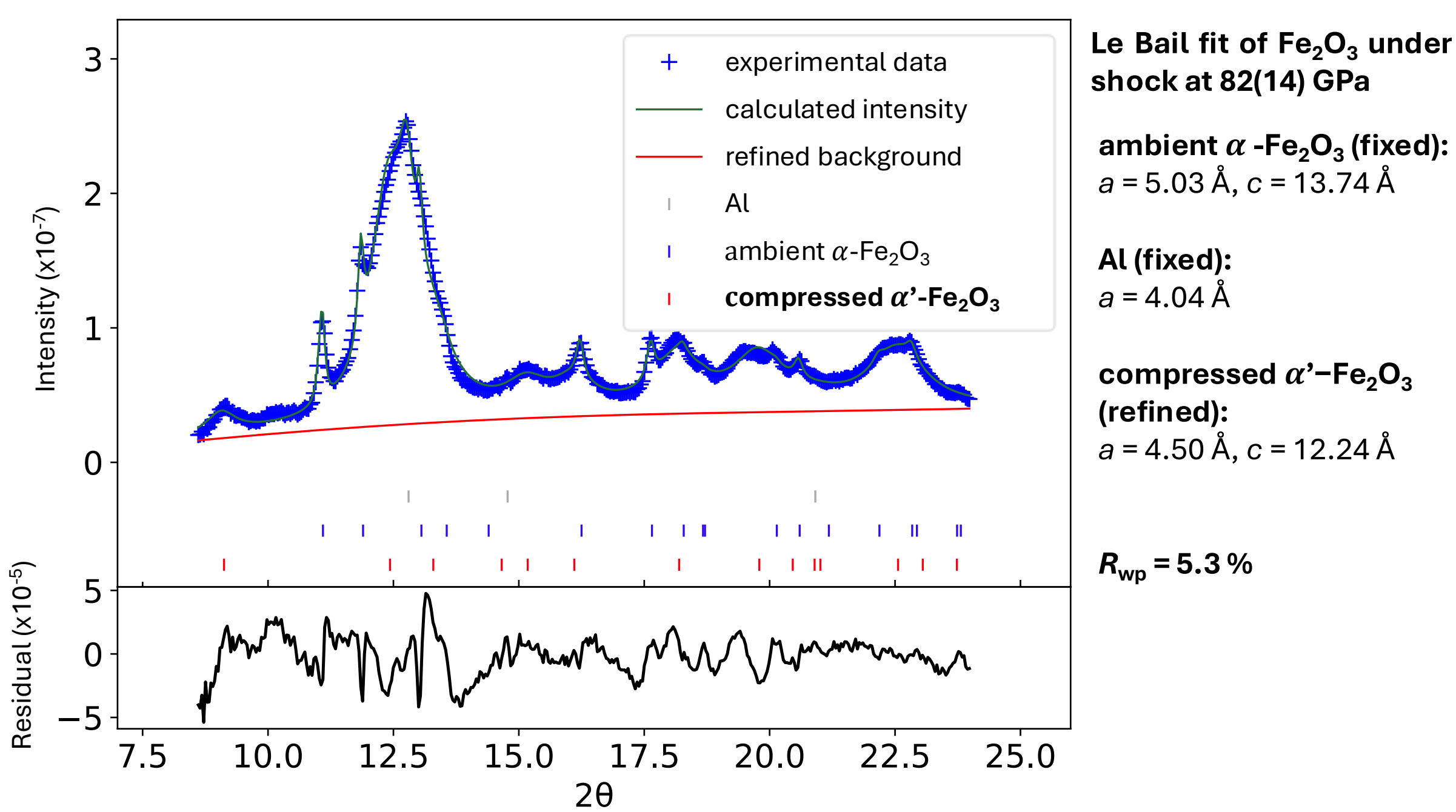

Figure S16. Le Bail refinement of shocked sample at 82(14) GPa performed on azimuthally integrated 1D XRD lineout after combining the two VAREX detectors and correcting the XRD intensity, as detailed in the Methods section C of the main paper. Cell parameters of ambient α-Fe_2O_3 phase and Al are fixed to the value found by Le Bail refinement of the ambient sample (Fig. S14). Fitted cell parameters of compressed α'-Fe_2O_3 phase are given, along with weighted profile R-factor (R_{wp}) of the fit.

When integrating separately the 2 Varex detectors we can see in Fig. S17 that a significant shift is observed in peak positions (between around 0.03 to 0.05 Å^{-1}) between the 2 Varex detectors for α-Fe_2O_3 under shock at 33(14) GPa whereas it is less visible for α'-Fe_2O_3 peaks. We note a small difference in peak position in the starting material linked to a small mismatch between the 2 Varex detector geometry calibration based on CeO_2 run. This mismatch is smaller than 0.007 Å^{-1} and is thus significantly smaller than the shift observed between the position of α-Fe_2O_3 peaks between the two Varex detectors. The shift of Bragg peak position in function of the azimuthal angle observed in Fig. S18 for α-Fe_2O_3 under shock at 33(14) GPa can be linked to strength effect which implies a variation of the Bragg peak position in function of the azimuthal angle [29]. Columnar texture of the sample explains the asymmetry observed between the 2 Varex detectors.

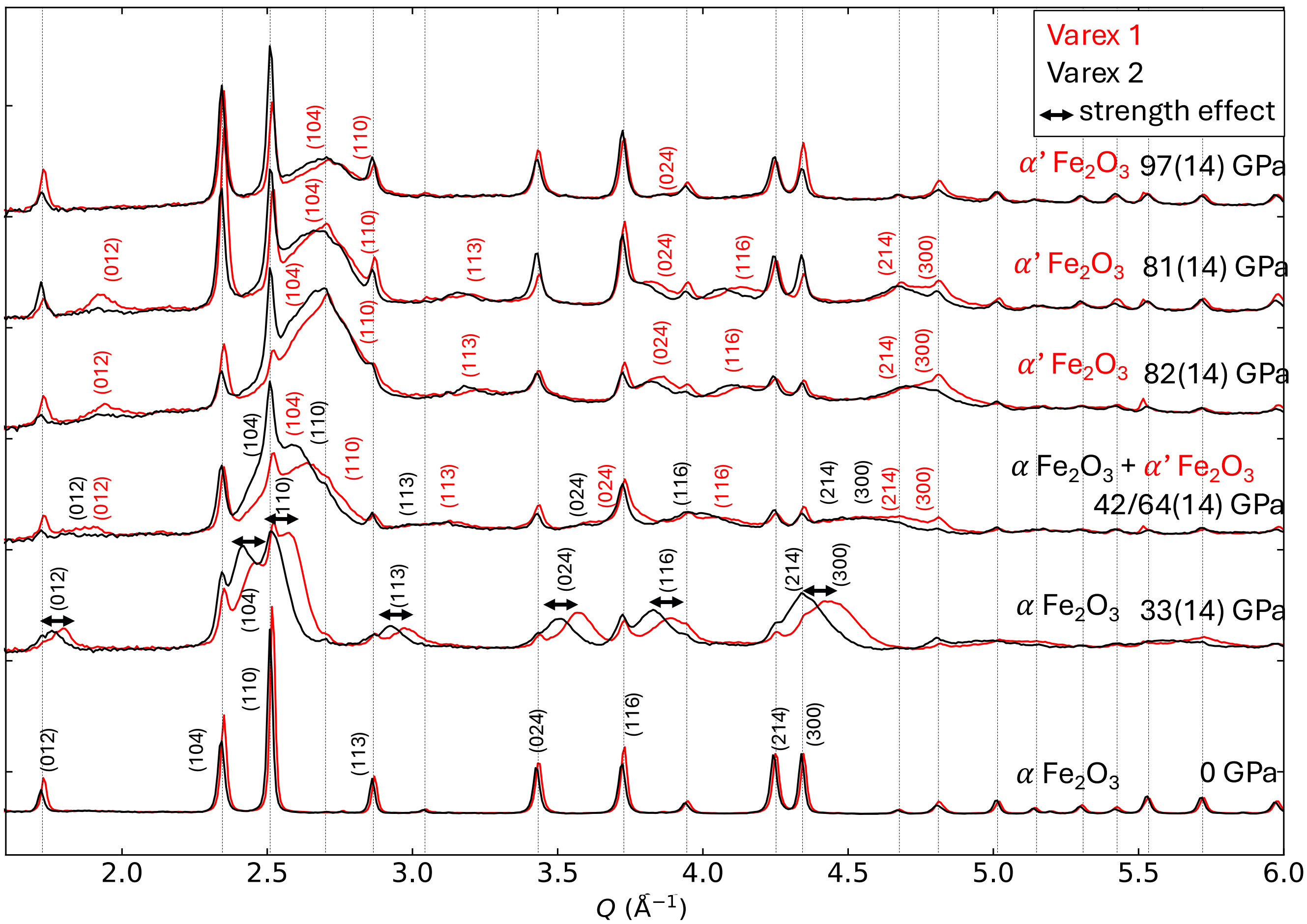

Figure S17. Azimuthally integrated 1D lineout for Fe_2O_3 under shock. We can see α to α'-Fe_2O_3 transition. A significant shift of Bragg peak position ($>$ 0.03 Å) indicated by black arrows is observed between Varex 1 and Varex 2 for the α phase under shock, which is interpreted as a strength effect.

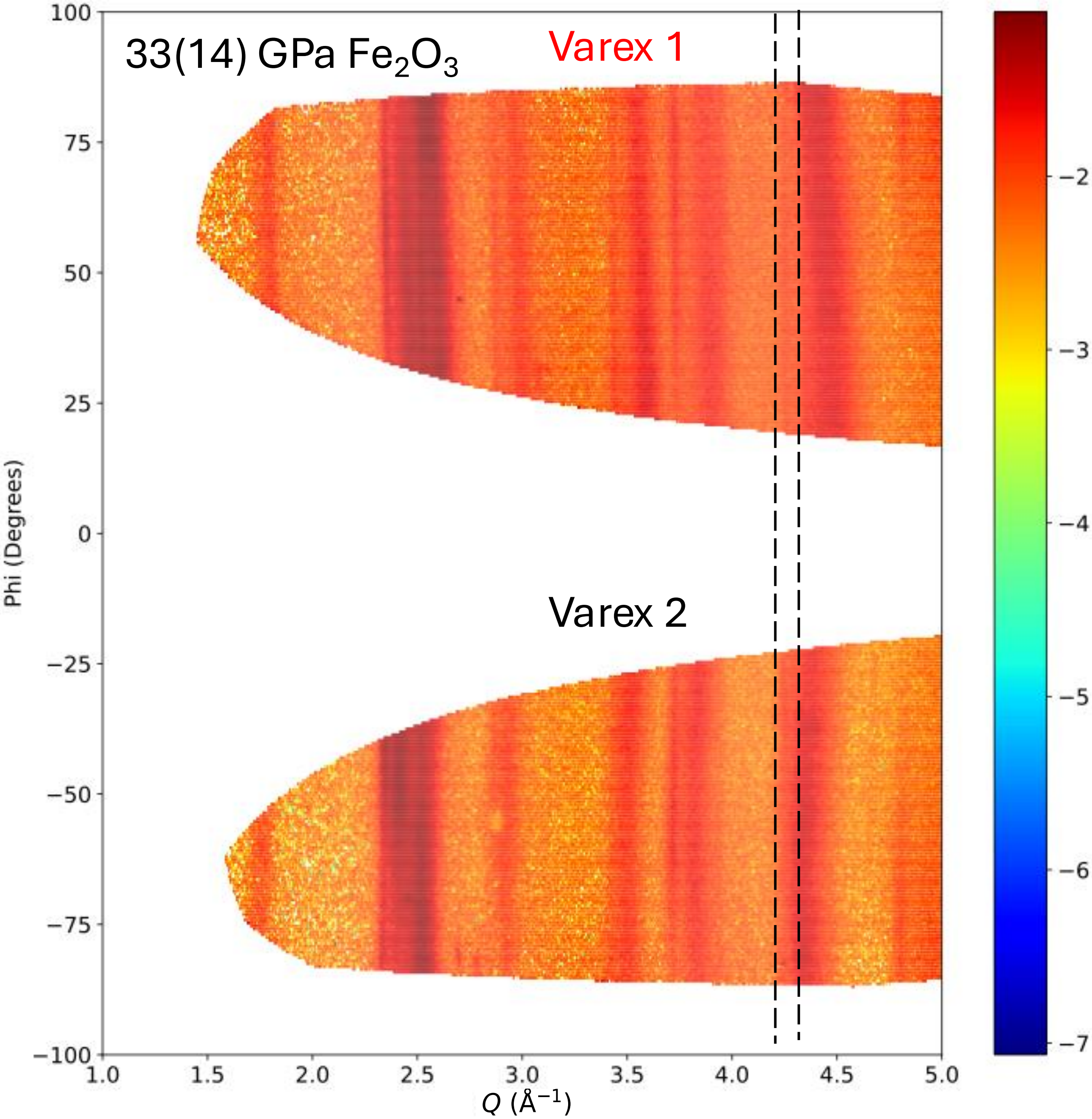

Figure S18. 2D XRD dewarped image for α-Fe_2O_3 under shock. Phi is the azimuthal angle. Vertical dashed black lines are used to help assessing the bending of the lines in link with the strength effect.

IX. RELEASE STUDY IN AMORPHOUS FE$_2$O$_3$

Two peaks are observed in Fe_2O_3 amorphous phase at 117(14), 147(14) and 166(14) GPa at 3.2 and 3.5 Å^{-1}. They are clearly shifting toward higher scattering vector with pressure. These two peaks are seen to become more intense upon release although they are not clearly shifting in scattering vectors as seen in Fig. S19. They could be linked to recrystallization of Fe_2O_3 in a hot α or α'-Fe_2O_3 upon release.

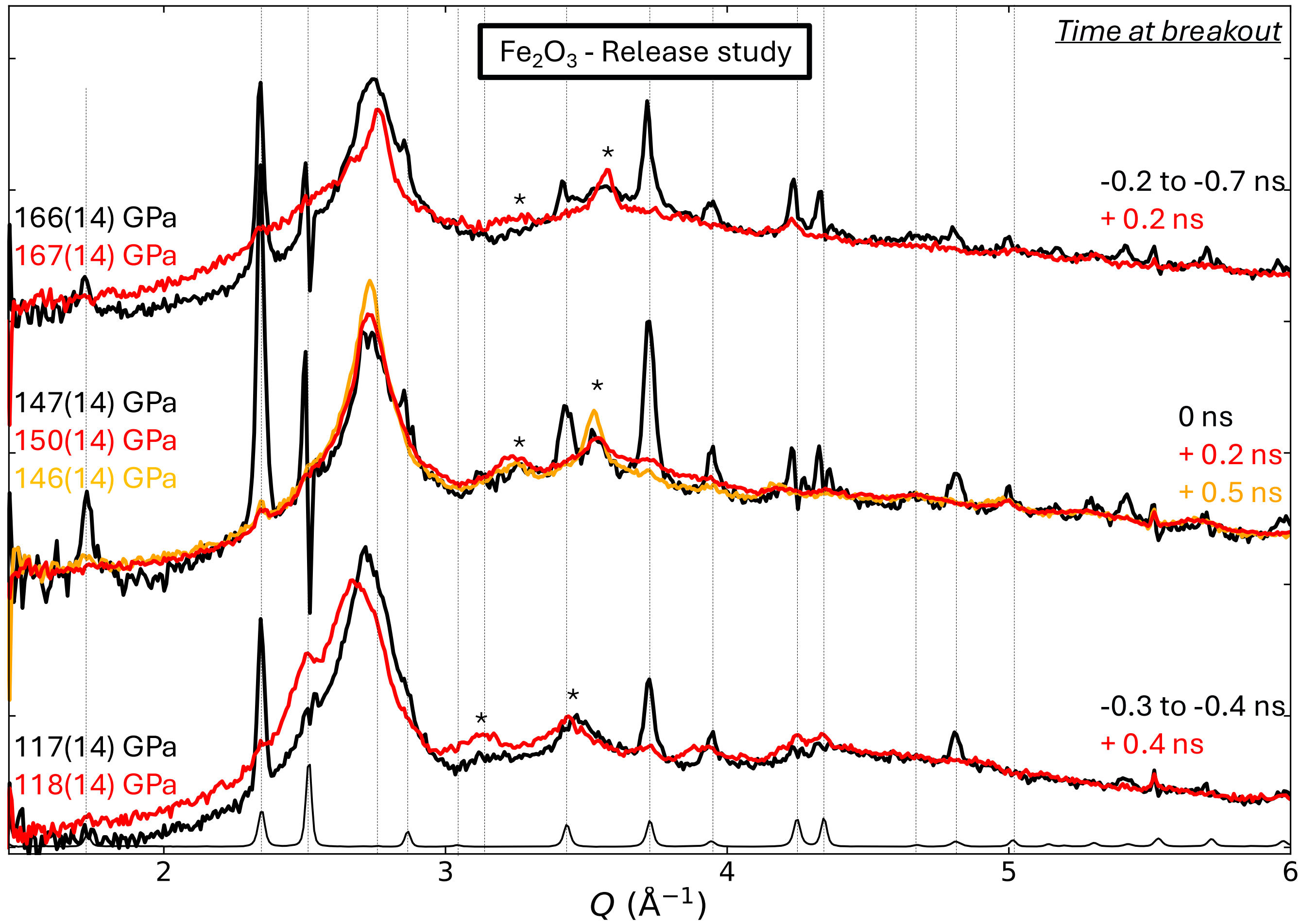

Figure S19. Azimuthally integrated 1D lineout for Fe_2O_3 under shock and release in the amorphous state from 117(14) to 166(14) GPa. The time at which the sample is probed compared to the breakout time, which is the time when the shock exits the Fe_2O_3 layer, is indicated on the right. For data points without VISAR measurements (at 147(14) and 146(14) GPa as well as one of the two summed runs at 166(14) GPa), breakout times are assumed to be the same to the ones measured for run performed at the same laser conditions. For run at 147(14) GPa this approximation seems incorrect as ambient peaks are still clearly visible.

X. MELT AND AMORPHOUS IN TWO Fe_2O_3 SAMPLES

Two Fe_2O_3 samples described in Section I, have been studied under shock. Fe_2O_3 poorly crystalline is shown to be highly textured as strong variations in XRD intensity are observed along the azimuthal angle in Fig. S20. Moreover, Fe_2O_3 poorly crystalline has a slightly larger volume, as Bragg peaks are shifted slightly toward lower scattering vector. This difference in volume is explained by differences in the sputtering process used to deposit the samples.

Under shock we can see that the XRD signal at 247(14) and 253(14) GPa is similar for both samples in Fig. S21. However, at 147 and 135 GPa, differences in the height and the sharpness of the first diffraction peak are observed. Moreover, this first diffraction peak is slightly shifted toward lower scattering angle for Fe_2O_3 poorly crystalline, which we linked to the initial larger volume of the sample. The differences observed between the two samples at 135-147 GPa are explained by an amorphous phase retaining the initial crystallinity of the sample as well as its initial shift in volume, whereas similarity of the signal at 247-253 GPa is attributed to a melt.

Two peaks are present in the amorphous phase at 135-147 GPa, the first peak is clearly visible for both samples. The 2 peaks are still visible at 166(14) GPa as also seen in Fig. 5 of the main paper. These peaks could be attributed to α' Fe_2O_3 Bragg peaks (113) and possibly (024). They are seen to grow in release in Fig. S19.

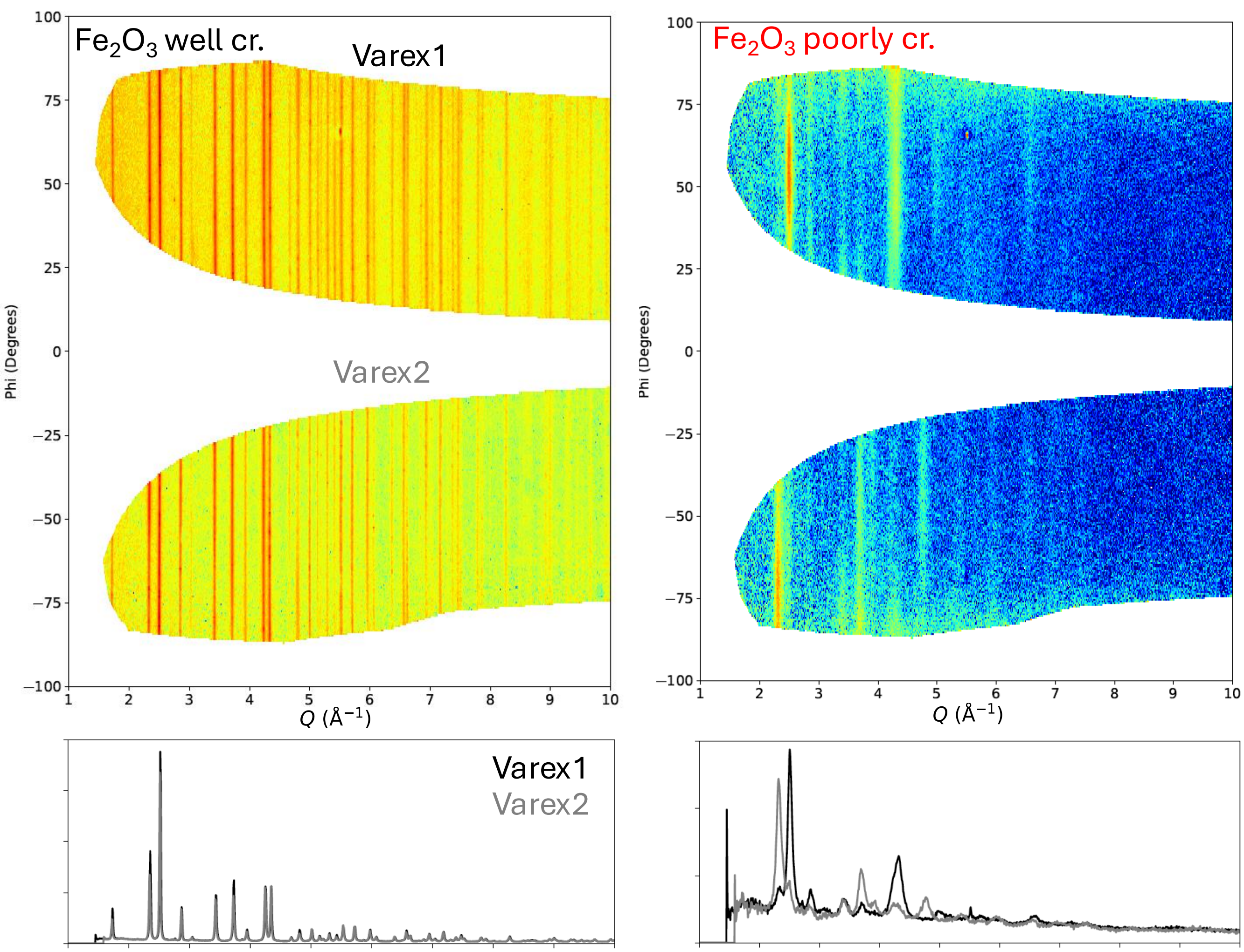

Figure S20. Dewarped 2D XRD images and corresponding azimuthally integrated lineout for ambient Fe_2O_3 poorly crystalline (cr.) and Fe_2O_3 well crystalline. Crystallinity of the sample is different due to difference in the synthesis via sputtering process as described in Section I. Poorly crystalline sample is shown to be heavily textured.

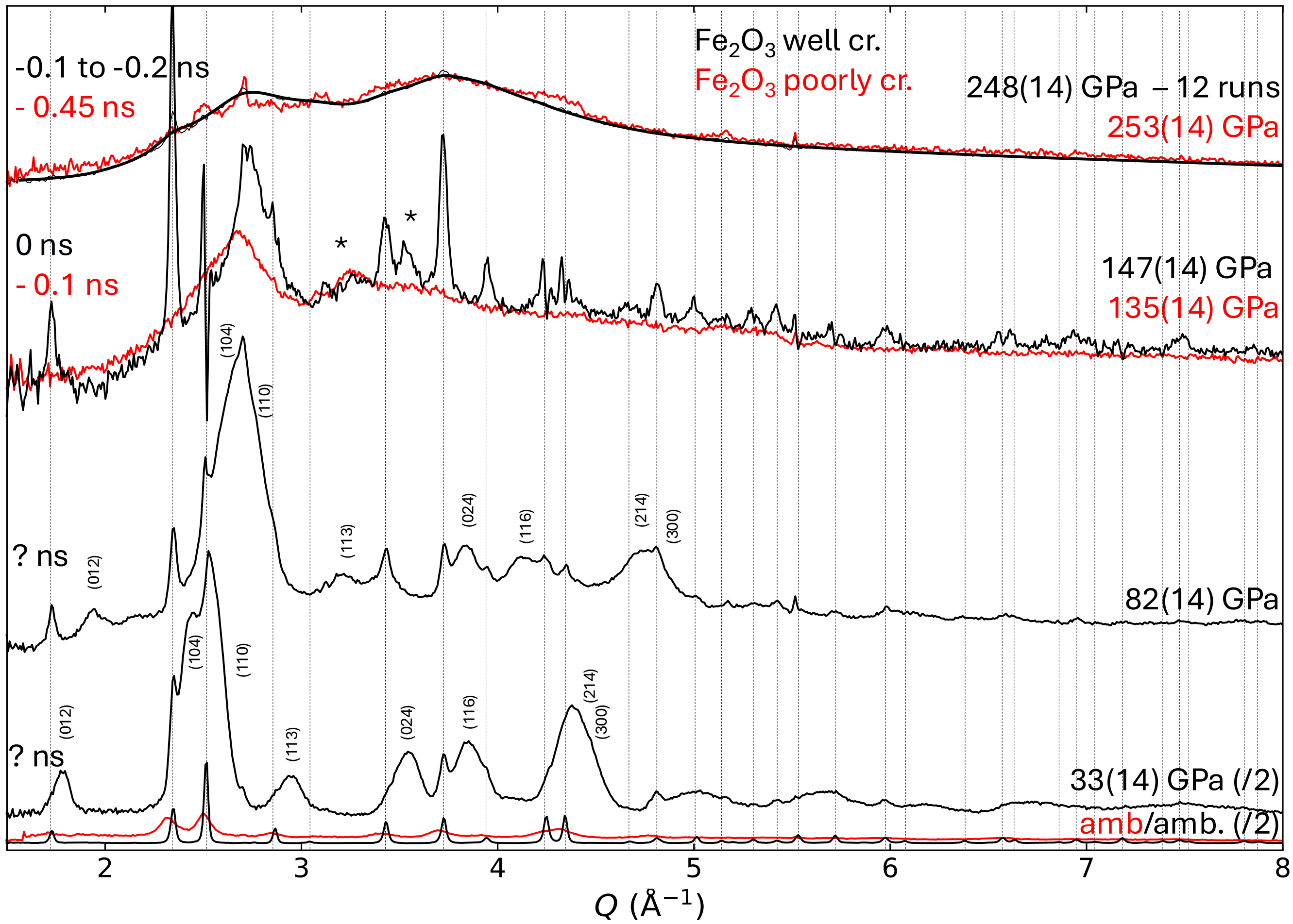

Figure S21. Azimuthally integrated 1D lineout for Fe_2O_3 ambient (amb.) and under shock for both well and poorly crystalline (cr.) Fe_2O_3 samples. Times to breakout time, i.e. the time at which the shock exits the target are indicated on the left. This is not known for run at 33(14) and 82(14) GPa as no VISAR was measured at these conditions. Moreover, for run at 147 (14) GPa and for 10 of the 12 runs at 247(14) GPa, breakout times are assumed to be the same to the ones measured for runs performed at the same laser conditions. For run at 147(14) GPa this approximation seems incorrect as ambient peaks are still clearly visible. Two peaks indicated by a black star are seen in the amorphous phase and could be attributed to remaining α' Fe_2O_3 Bragg peaks (113) and possibly (024).

XI. LIQUID DIFFRACTION ANALYSIS

The Faber-Ziman structure factor $S_{\mathrm{FZ}}(Q)$ was calculated as [30] :

$$S_{\mathrm{FZ}}(Q) = \frac{I^{\mathrm{coh}}(Q) - (\langle f^2 \rangle - \langle f \rangle^2)}{\langle f \rangle^2}, \tag{S4}$$

with $\langle f^2 \rangle$ and $\langle f \rangle^2$ the average effective electronic form factor calculated using atomic form factor from Hadju [31] and I^{coh} the coherent intensity of the XRD profile defined as

$$I^{\mathrm{coh}}(Q) = \alpha^{\mathrm{FZ}} I(Q) - \frac{1}{N} \sum_p I_p^{\mathrm{incoh}}(Q), \tag{S5}$$

with I_p^{incoh} the incoherent Compton scattering intensity [31], N the number of atoms, p the different atomic species, and α^{FZ} a normalization factor [30]. We used XRD data up to approximately 12 Å^{-1} for Fe and 9-10 Å^{-1} for iron oxide melts, as detailed in Section XI. The structure factor was extrapolated to a value of 1 up to 10 Å^{-1} for iron oxides, where needed.

We calculate the Fourier transform of the structure factor as

$$F(r) = \frac{2}{\pi} \int_0^{Q_{\max}} \sin(Qr)[S_{FZ}(Q) - 1]\,\mathrm{d}Q, \tag{S6}$$

with r the radial interatomic distance and $Q_{\max}$ the highest available scattering momentum. The pair distribution function $g(r)$ is then given by

$$g(r) = \frac{n(r)}{n_0} = 1 + \frac{F(r)}{4\pi n_0}, \tag{S7}$$

with $n(r)$ the atomic density at a distance r from a given atom and n_0 the average atomic density at the experimental conditions. To determine $g(r)$ we use an iterative process [32–34]. We minimize the background in $g(r)$ for the smallest interatomic distances ($r < r_{\min}$) where no real signal is expected:

$$\begin{gathered} r < \mathrm{r}_{\min} \Rightarrow g(r) = 0 \Rightarrow F(r) = -4\pi r n_0 \\ \Delta F(r) = F(r) + 4\pi r n_0 \end{gathered} \tag{S8}$$

where $\Delta F(r)$ denotes the uncertainty in $F(r)$. $\Delta F(r)$ is then evaluated and subtracted from $F(r)$ to obtain the corresponding $g(r)$, from which an updated $S_{\mathrm{FZ}}(Q)$ is calculated. This process is iterated until convergence. The residual $\Delta F(r)$ is minimized by refining both the density n_0 and the cutoff distance $r_{\min}$. To achieve this, we generate residual maps showing the variation of $\Delta F(r)$ as a function of n_0 and $r_{\min}$ for each dataset. Initial estimates of n_0 and $r_{\min}$ are obtained from these maps, after which both parameters are refined using the BOBYQA optimization algorithm.

We determine a smooth baseline from the fully corrected XRD intensity as shown in figs. 3,4,5 of the main paper. Faber-Ziman structure factor is then calculated by using data point up to around 9 Å^{-1} for iron oxide and 12 Å^{-1} for Fe. Fe dataset has a higher signal-over-noise ratio in link with higher atomic number and thicker sample, which explain why we could use the data till larger Q range without producing an artefact in the pair distribution function. Faber-Ziman structure factor is extrapolated to a value of 1 at higher scattering vector and termination ensured to be smooth as shown in Fig. S22,Fig. S23,Fig. S24. Maximal value of scattering vector used to determine the pair distribution function is then fixed to 9-10 Å^{-1} for iron oxide and 11.9-12 Å^{-1} for Fe as indicated in Fig. S26,Fig. S27,Fig. S28. Faber-Ziman structure factor is extrapolated down to 0 Å^{-1} (below 1.6 Å^{-1}) to the value measured at the lowest accessible scattering vector.

During calculations of the pair distribution via the iterative process described above we minimize the residual $\Delta F(r)$. In Fig. S25,Fig. S26,Fig. S27,Fig. S28 we represent the value of $\Delta F(r)$ in function of $\mathrm{r}_{\min}$ and the density. A slight ambiguity can arise in the choice of a $\mathrm{r}_{\min}$/density pair as different values can lead to a minimization of $\Delta F(r)$ within a given interval. Our choice has been guided (within an acceptable value of the density) by (1) the value of the residual $\Delta F(r)$, (2) the minimization of oscillation at low interatomic distance, and (3) the consistency of $\mathrm{r}_{\min}$ across one type of sample as we expect it to be relatively similar.

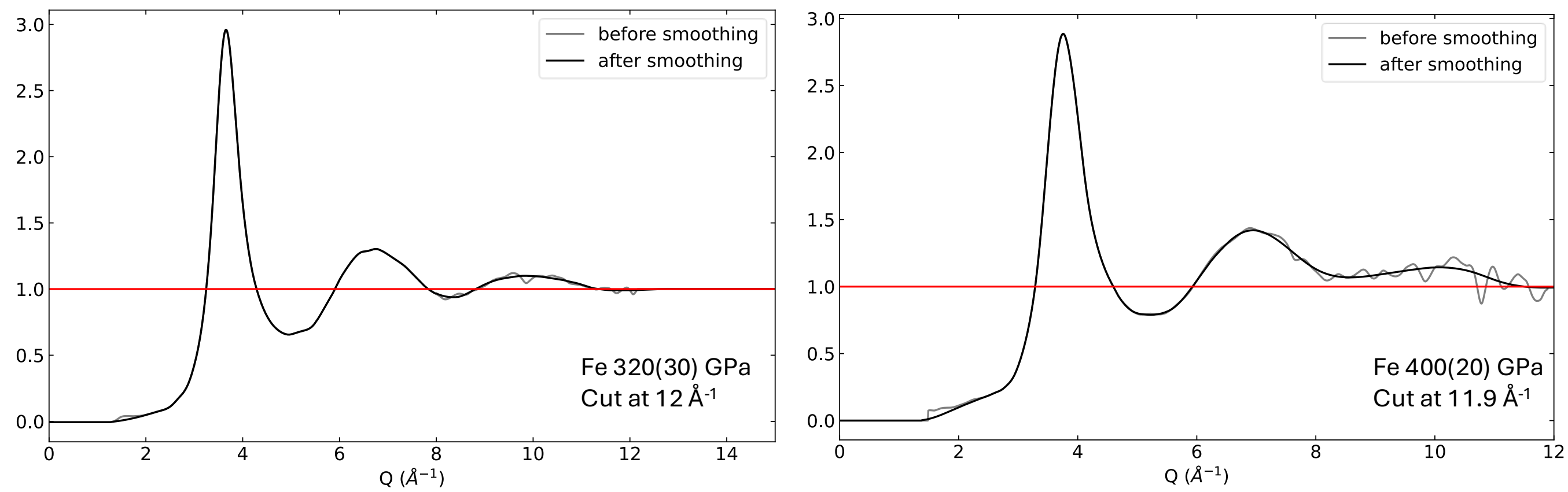

Figure S22. Initial Faber-Ziman structure factor for Fe melt under shock calculated by considering the fully corrected 1D lineout azimuthally integrated XRD signal. XRD signal was cut at the value indicated on each graph and prolongated to a limit value of 1. Termination was then carefully smoothed to avoid artifact when calculating the pair distribution function.

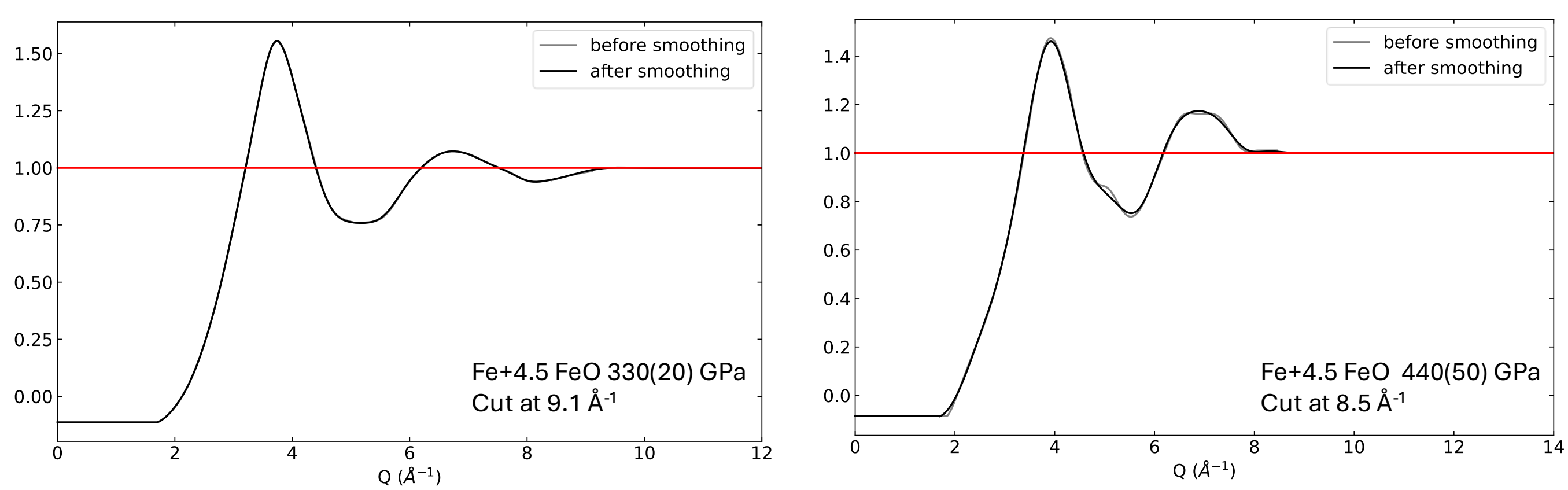

Figure S23. Initial Faber-Ziman structure factor for Fe + 4.5 FeO melt under shock calculated by considering the fully corrected 1D lineout azimuthally integrated XRD signal. XRD signal was cut at the value indicated on each graph and prolongated to a limit value of 1. Termination was then carefully work out to avoid artifact when calculating the pair distribution function.

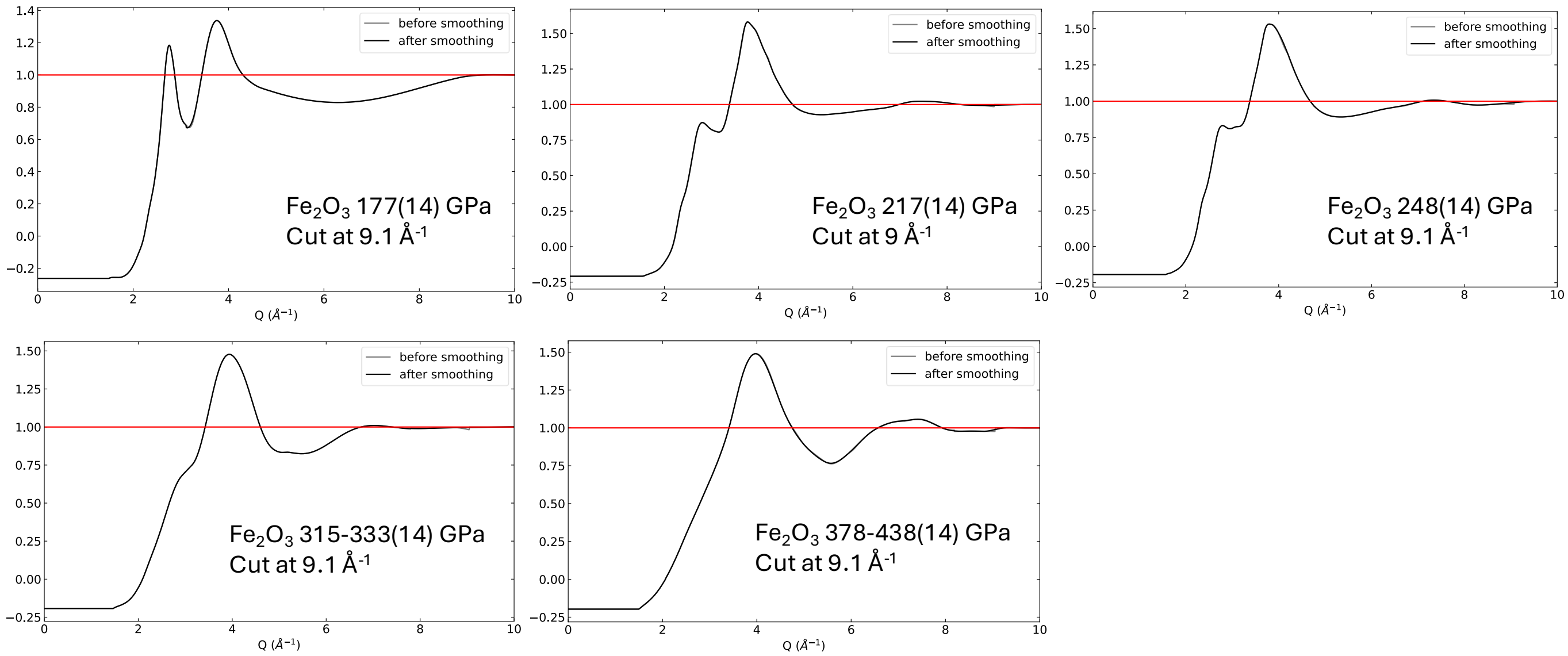

Figure S24. Initial Faber-Ziman structure factor for Fe_2O_3 melt under shock calculated by considering the fully corrected 1D lineout azimuthally integrated XRD signal. XRD signal was cut at the value indicated on each graph and prolongated to a limit value of 1. Termination was then carefully work out to avoid artifact when calculating the pair distribution function.

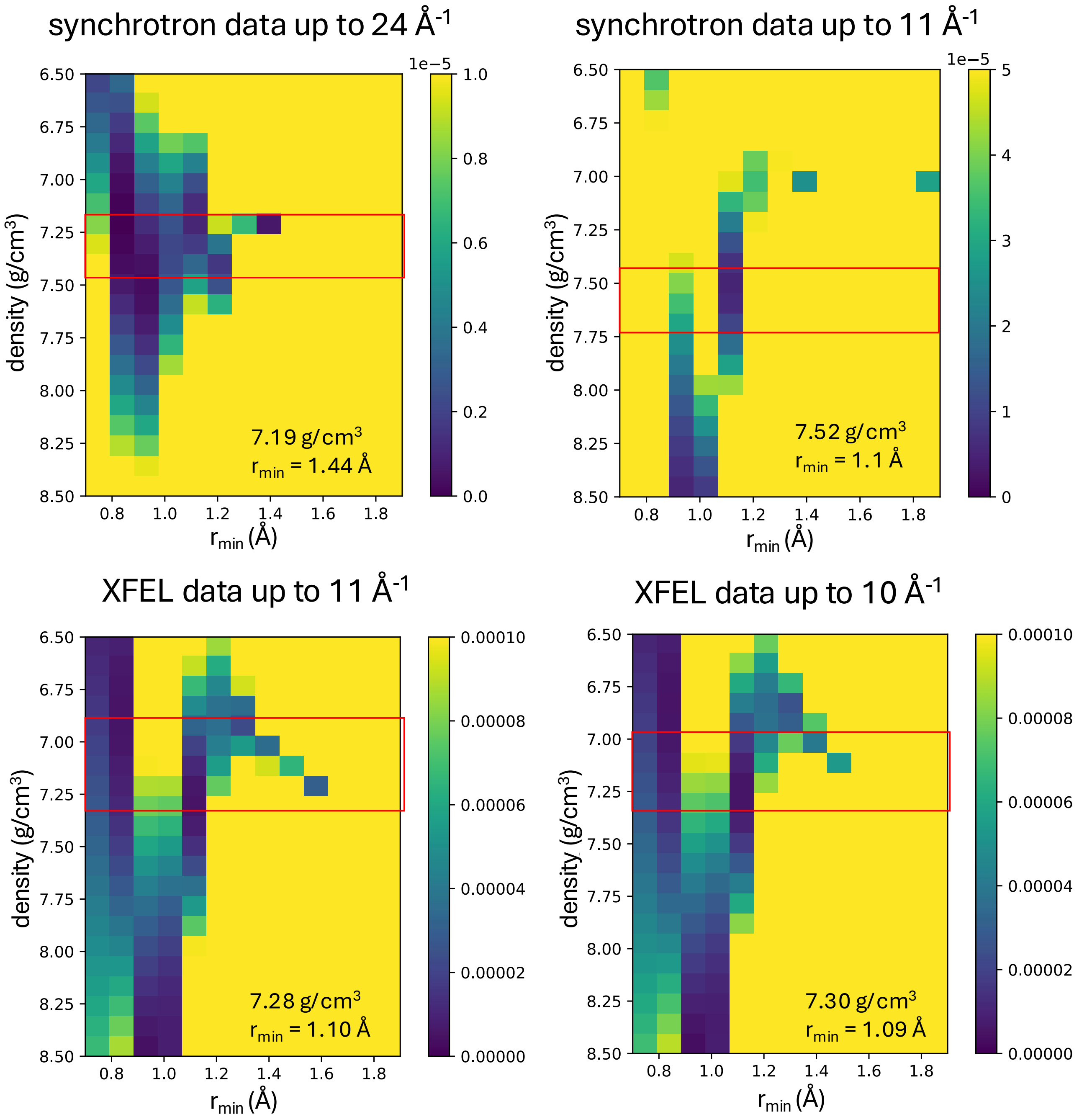

Figure S25. Map of residual for the metallic glass in function of r_{min} and the density. We can see that different r_{min} can lead to different density, the range of which is highlighted in red.

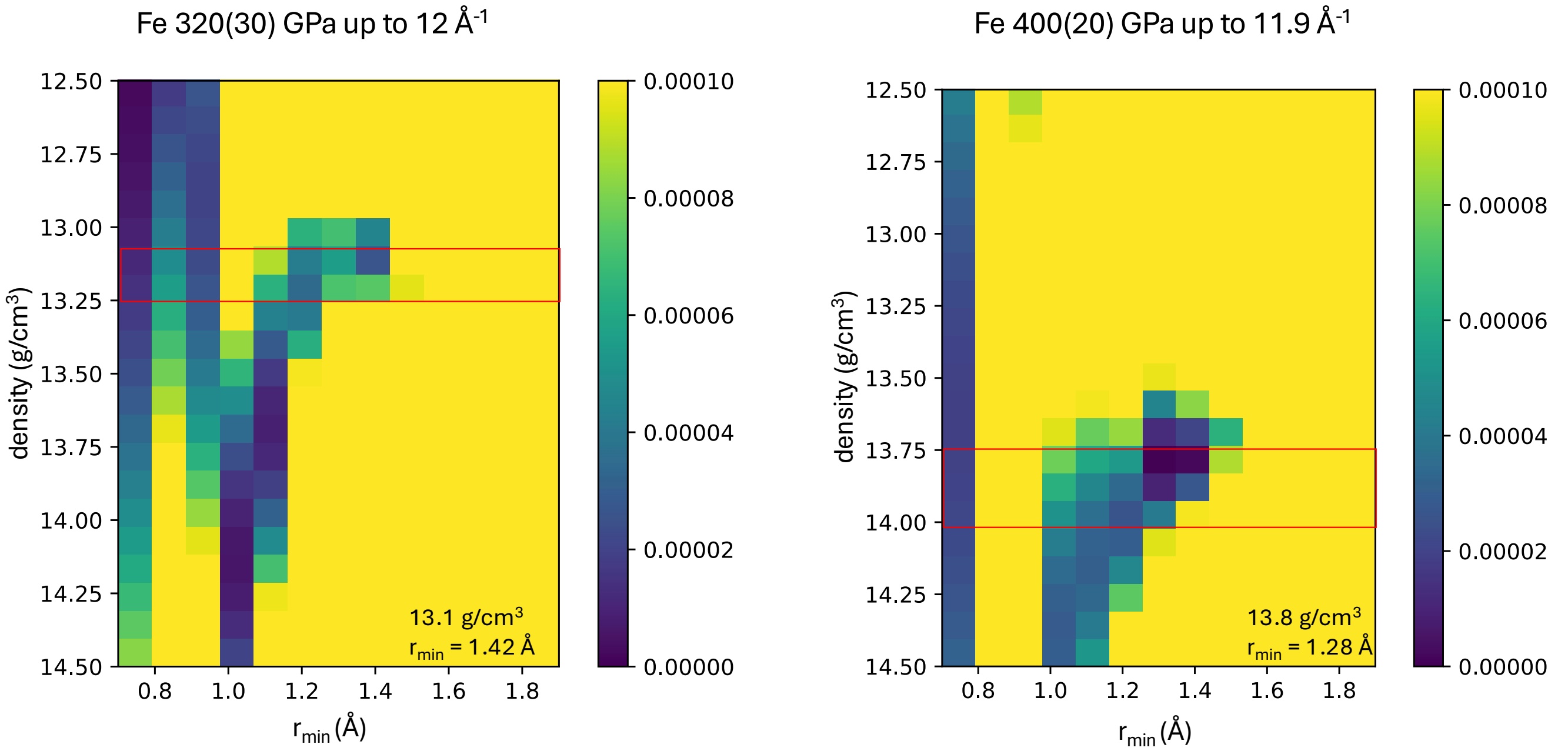

Figure S26. Map of residual for Fe in function of r_{min} and the density. We can see that different r_{min} can lead to different density, the range of which is highlighted in red.

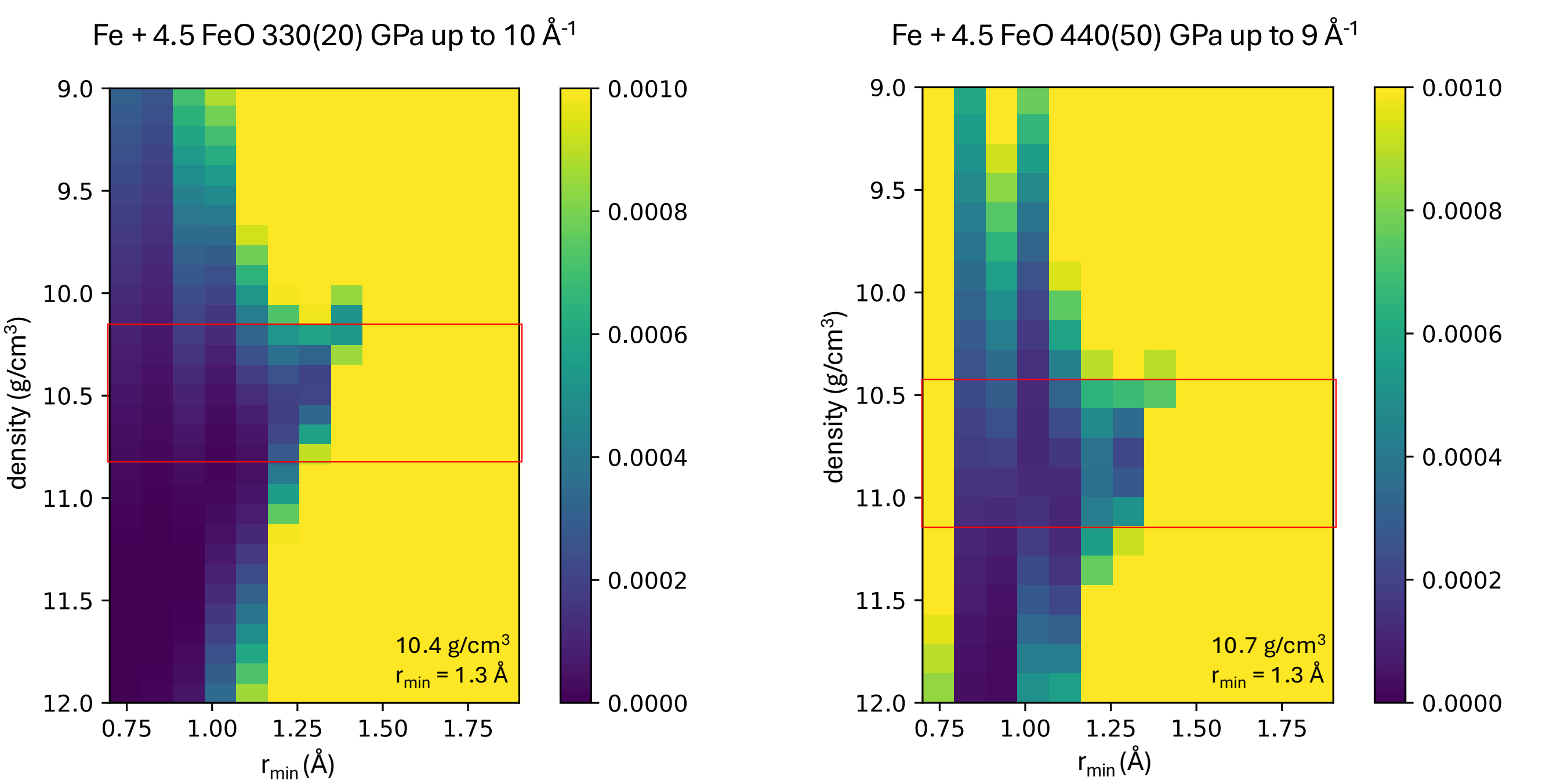

Figure S27. Map of residual for Fe + 4.5 FeO in function of r_{min} and the density. We can see that different r_{min} can lead to different density, the range of which is highlighted in red.

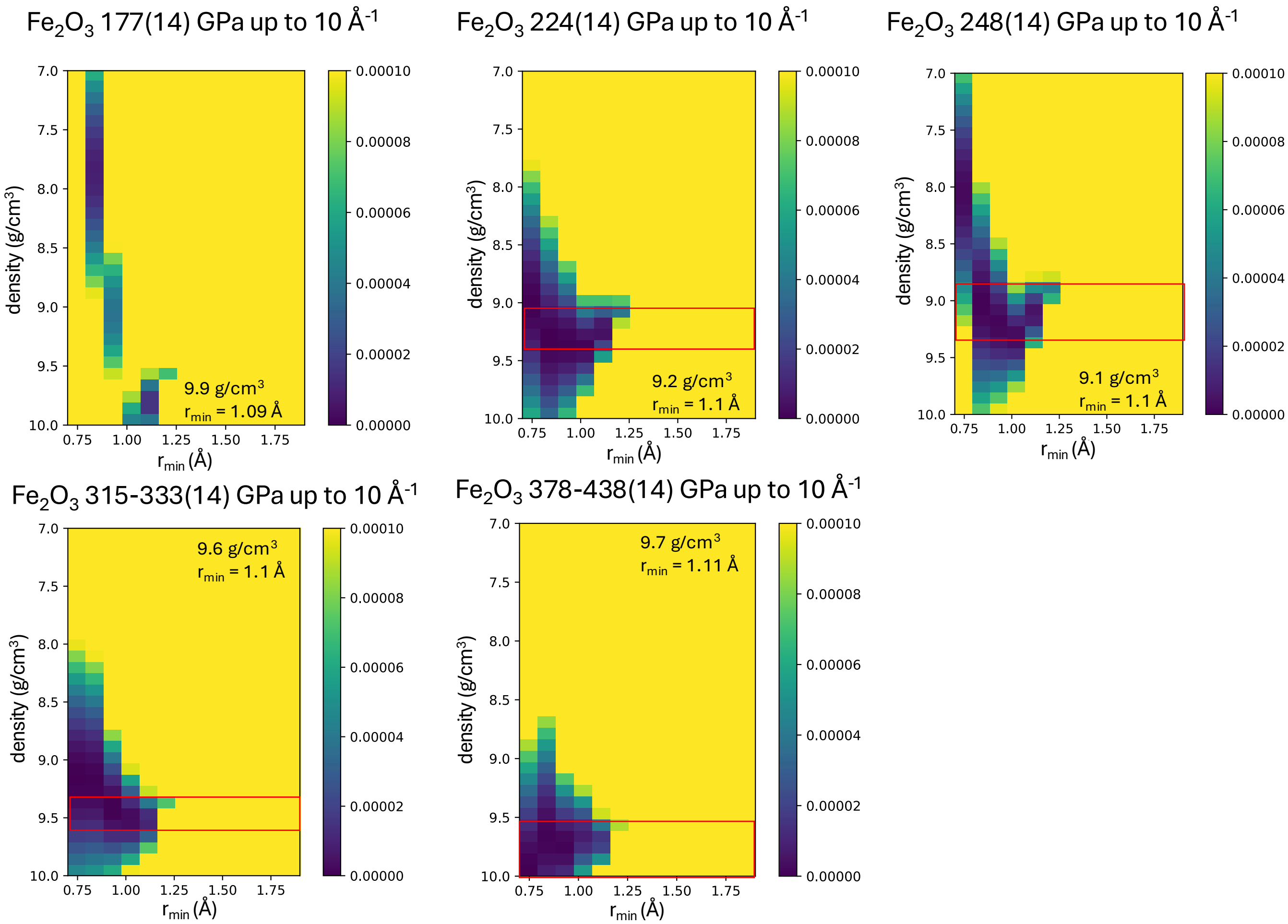

Figure S28. Map of residual for Fe_2O_3 in function of r_{min} and the density. We can see that different r_{min} can lead to different density, the range of which is highlighted in red. Data point at 177(14) GPa shows however that very different values of r_{min} and density are possible unlike other data point.

XII. COORDINATION NUMBER AND INTERATOMIC DISTANCES EVALUATION

Coordination number can be calculated from the area below the radial distribution function, $\mathcal{R}(r)$, defined as

$$\mathcal{R}(r) = 4\pi r^2 g(r) n_0, \tag{S9}$$

where n_0 is the atomic density. for monoatomic melt such as Fe. In Fig. S29, we can see that the area below the first oscillation of the $\mathcal{R}(r)$ for the metallic glass for the present measurement at XFEL cut at 10 Å is smaller by 0.21 compared to the measurement from the synchrotron.

We calculate the coordination number for Fe using the $\mathcal{R}(r)$ in Fig. S30. To avoid spurious oscillations remaining at low interatomic distances for data point at 320(30) GPa the area below the first oscillation was only integrated from 1.58 Å (indicated by a black vertical line). The area below the first oscillation was integrated up to the first minimum following this first oscillation which is indicated as a black, grey or blue vertical line for respectively present data points at 320(30) GPa, 400(20) GPa and data point from Singh et al., 2023 [26] at 275(9) GPa. We note that Singh et al., 2023 reported a Fe-Fe CN of 12.9 at this condition [26], the difference of 0.1 in CN might only be linked to the digitization of the data. Smaller CN at 400(20) GPa is linked to small shock fraction (49%) compared to the entire dataset where shock fraction was found to be superior to 79%. We estimated a ±0.4 error on CN based on data point at 320(30) GPa, assuming a Fe-Fe CN of 12.7 based on both theoretical and experimental studies [26, 35] as discussed in the main paper.

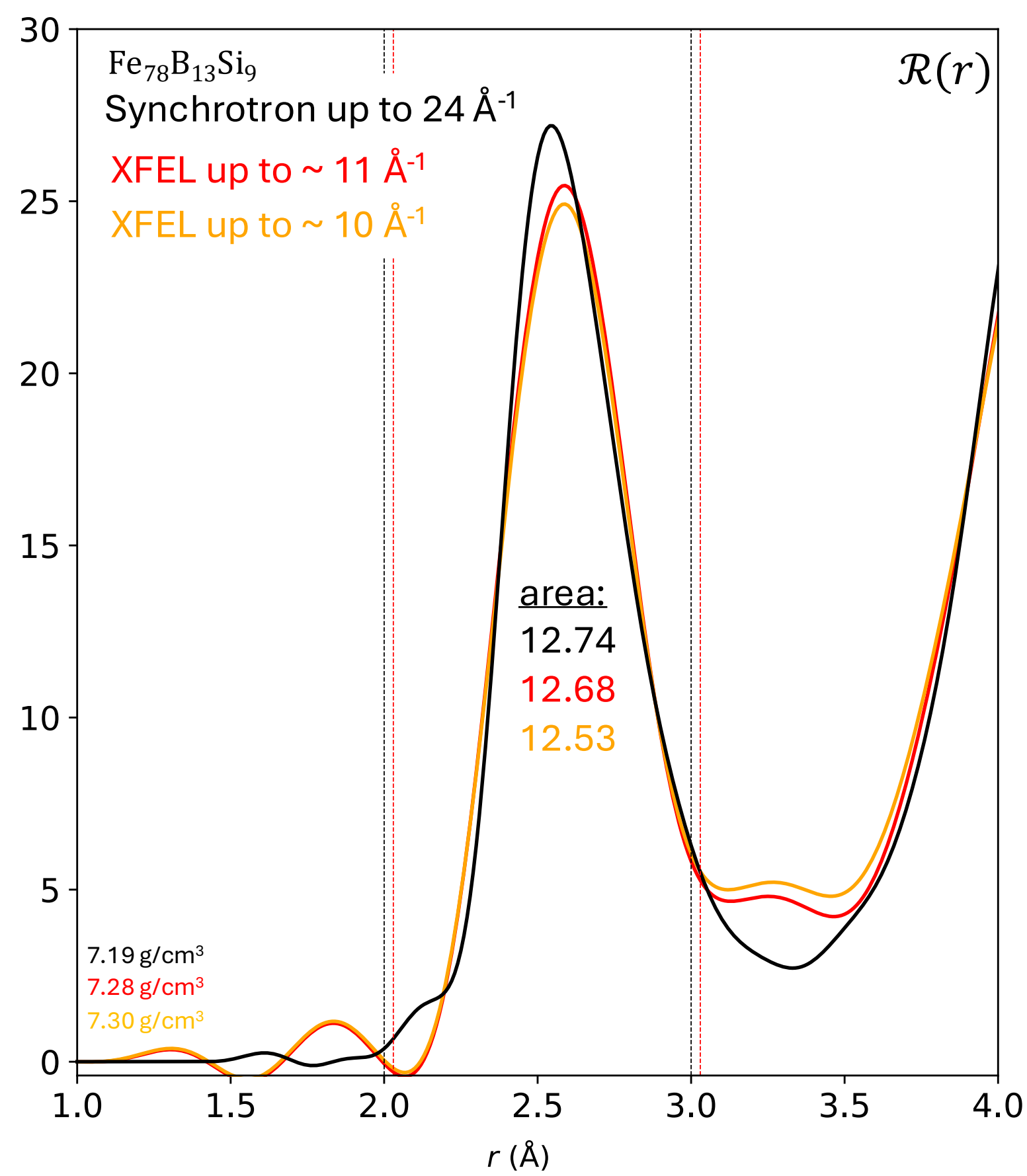

Figure S29. Radial distribution function, $\mathcal{R}(r)$, calculated for the metallic glass. Area delimited by the 2 vertical black dotted lines is integrated for the synchrotron data, and area delimited by the 2 vertical red dotted lines for XFEL data. We avoid spurious oscillations around 3 Å by cutting the area of interest below. Difference between the integrated area is 0.21 at the maximum.

For polyatomic melts (i.e. Fe_2O_3 and Fe + 4.5 FeO) we use Gaussian fitting of the first oscillation of $T(r) = 4\pi r g(r) n_0$ function following procedure described in Heinen and Drewitt, 2022 [36]. Fits are reported in Fig. S31. O-O contribution was neglected due to very small contribution relatively to Fe-O and Fe-Fe in link with the difference

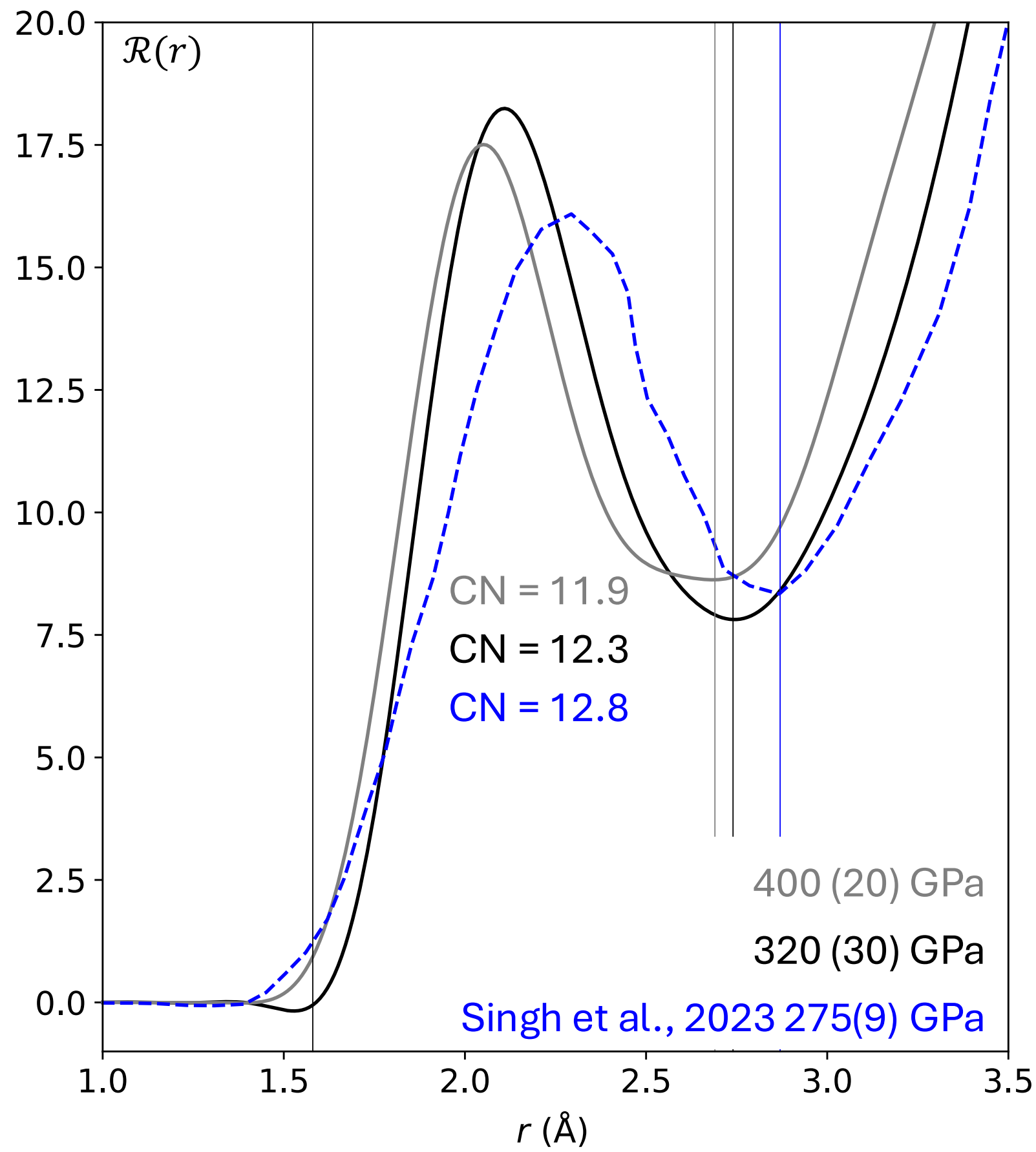

Figure S30. Radial distribution function for Fe under shock. Area under the first oscillation represents the Fe-Fe coordination number (CN). We can see that present data seem to give lower CN than expected, by around 0.4 for a shock fraction of 79%, i.e. data point at 320(30) GPa while low shock fraction can induce a significantly larger error bar as seen for data point at 400(20) GPa with a shock fraction of 49%.

in atomic numbers. We keep a similar errors on CN, density and interatomic distance as the ones determined for Fe at 320(30) GPa although it is difficult to correctly determined an error for CN for polyatomic melt as it also depends on the goodness of the fit.

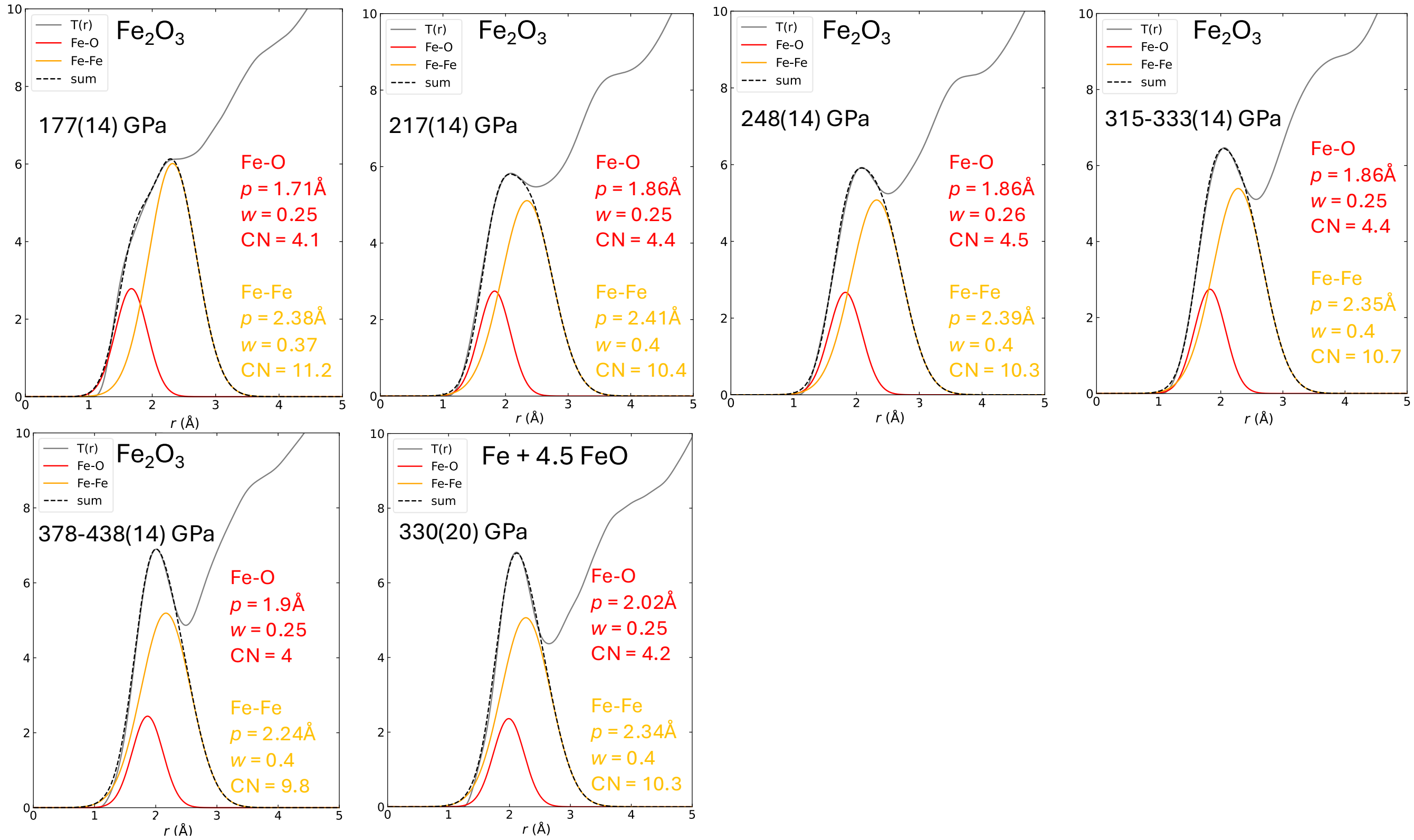

Figure S31. Gaussian fit for Fe-O and Fe-Fe contribution for the different iron oxide melts data points. We fit the maximum position of the Gaussian, p, the width, w and the area, the latter corresponds to the coordination number (CN).

XIII. UNCERTAINTY QUANTIFICATION OF ANALYSIS FROM LIQUID DIFFRACTION RESULTS

X-ray diffraction of liquids to obtain pair distribution functions and densities is well established under static compression [37], but remains rare for non-monoatomic compositions under dynamic (shock) conditions [38, 39]. Therefore, we place particular emphasis on validating the method and quantifying uncertainties in interatomic distances, coordination numbers (CN), and densities. In this aim (1) XRD intensities were carefully corrected for solid angle, polarization, the Al filter on the Varex detector, self-attenuation, ablator contribution, and residual ambient material, as detailed in Methods of the main paper and (2) we analysed a commercial metallic glass from Goodfellow ($Fe_{78}B_{13}Si_9$), previously measured at the Diamond Light Source synchrotron (beamline ID15) [40], together with Fe melt under shock, i.e., a monoatomic melt extensively studied under extreme conditions both experimentally [26, 41] and theoretically [35, 42].

Liquid diffraction data for the metallic glass, obtained both from synchrotron measurements [40] and from the present XFEL setup, are shown in Fig.S32. Metallic glass was measured on the exact same glass at synchrotron Diamond Light Source. Four layers of the glass were used on the I15-1 diffractometer at Diamond Light Source (data measured by Daniel Irving as part of the I15-1 mail-in service, proposal CY39017). It was run in transmission geometry with a 2D detector placed behind the sample and perpendicular to the beam in standard configuration at 76 keV. The structure factors and corresponding pair distribution functions derived from the two measurements show overall good agreement. In the pair distribution function, the position of the first oscillation is shifted by +0.03Å, which we adopt as the uncertainty on interatomic distances in the following analyses. The area of the first main oscillation decreases by 1.6% in the present measurement relative to the synchrotron data, based on integration of the first peak in the radial distribution function (see Section XII of the present Supplementary Information). The difference between the full synchrotron measurement (up to 24 $Å^{-1}$) and the XFEL data truncated at 10 $Å^{-1}$ is 0.11 $g.cm^{-3}$ (1.5%). This is the same cut-off applied to the iron oxide data shown in Fig. S24.

Regarding Fe melt, we obtain Fe–Fe interatomic distances of 2.05(0.03) Å at 320(30) GPa and 2.00(0.03) Å at 400(20) GPa, in agreement with ab initio molecular dynamics simulations of liquid Fe under pressure [35], which report 2.05 Å at 323 GPa and 6,370 K (Fig. 7 of the Main paper). The Fe–Fe coordination numbers (CN) derived here are 12.3 at 320(30) GPa and 11.9 at 400(20) GPa, consistent with ab initio molecular dynamics predictions of 12.5–12.6 from 96 to 323 GPa [35], and with experimental values of 12.7 and 12.9 at 258(8) and 275(9) GPa, respectively [26]. The calculated melt densities are 13.1 $g.cm^{-3}$ at 320(30) GPa and 13.8 $g.cm^{-3}$ at 400(20) GPa, which agree with previous theoretical and experimental results within ∼0.4 $g.cm^{-3}$.

The Fe data point at 400(20) GPa has a relatively low shock fraction of ∼49%, resulting in greater uncertainty in the corrected XRD intensity according to Wark et al. [17]. All other data points used for liquid diffraction analysis have shock fractions above 79%, ensuring high accuracy in the corrected intensities [17]. Shock fractions for all measurements are listed in the Supplementary Data 1. We therefore use the Fe data point at 320(30) GPa (shock fraction 89%) to estimate uncertainties on the liquid density and coordination number (CN). No significant variation in corrected XRD accuracy was observed for shock fractions between 80% and 100% [17]. The resulting uncertainties are ±0.4 for CN (3.1%, assuming CN = 12.7 for Fe, based on ab initio molecular dynamics simulations and experimental values [26, 35, 41]) and ±0.3 $g.cm^{-3}$ (2.3%) for density. These errors are larger than those derived from the metallic glass measurements, indicating that additional uncertainties arise from subtraction of the ablator and residual ambient material, factors absent in the metallic glass case.

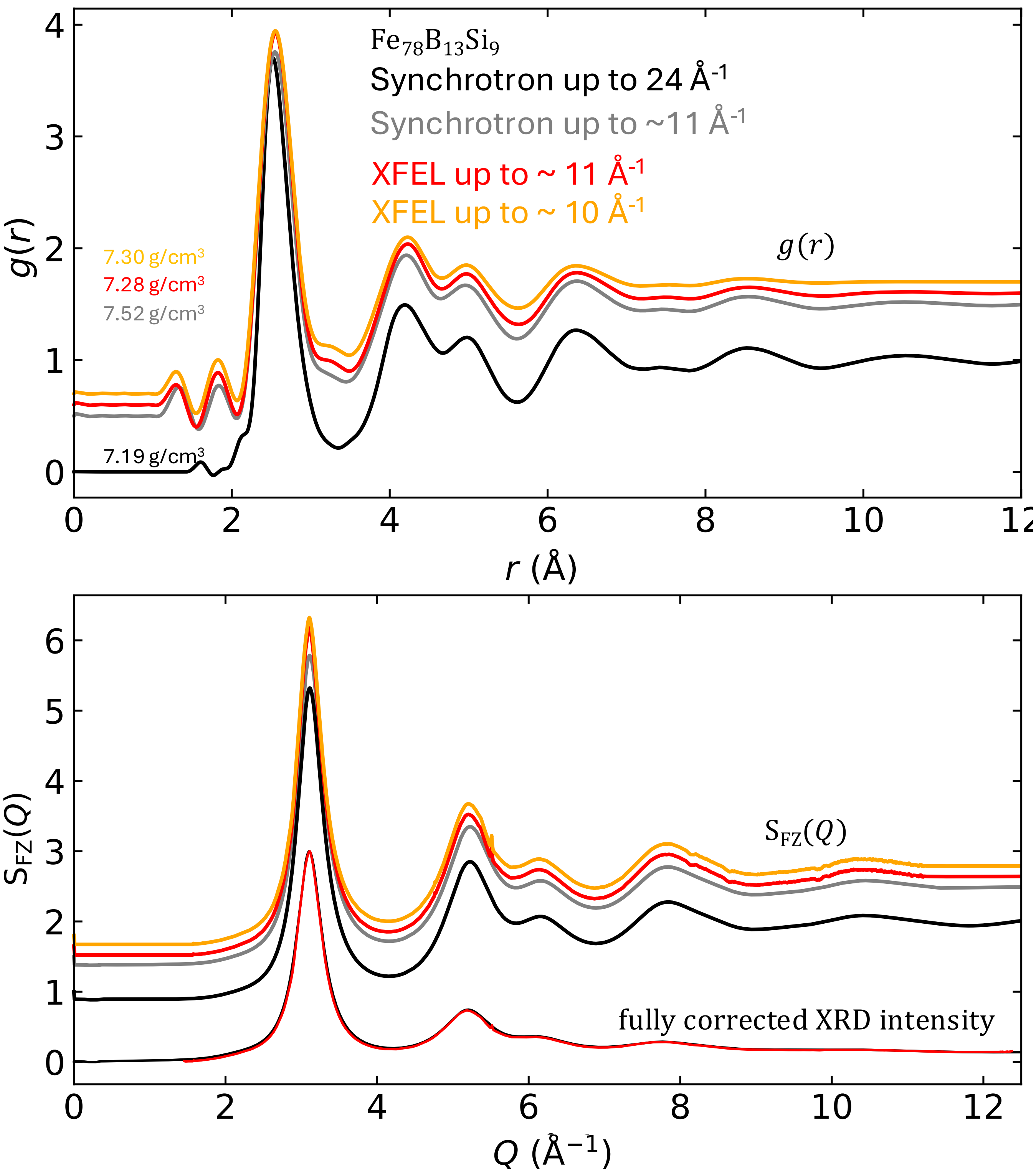

Figure S32. Fully corrected, azimuthally integrated 1D XRD lineout, Faber-Ziman structure factor $S_{FZ}(Q)$, and pair distribution function $g(r)$ for ambient commercial metallic glass ($Fe_{78}B_{13}Si_9$) measured both at the Diamond Light Source synchrotron (beamline I15-1, 76 keV) [40] and with the present experimental setup at 24 keV (XFEL).

XIV. HOMOGENEITY OF FE + 4.5 FEO MELT OBSERVED UNDER SHOCK

The Fe + 4.5 FeO sample was treated as a complete melt under two specific conditions:

- 330 GPa and 11,200(800) K – 10.4 g/cm^3, for which a 10 ns laser pulse was used.
- 440 GPa and 16,000(2,000) K – 10.7 g/cm^3, for which a 6 ns laser pulse was used.

These are extreme conditions, where diffusion coefficients (D) are poorly constrained. Posner et al., 2017 [43] reported, via ab initio molecular dynamics simulations, oxygen and iron diffusion coefficients in liquid Fe up to 5,200 K and 330 GPa. They found at this condition $D_O = 1.5 \times 10^{-8}$ m^2/s and $D_{Fe} = 0.58 \times 10^{-8}$ m^2/s, in relative agreement with Pozzo et al., 2013 [44]. Currently, no diffusion coefficient is available for iron oxide melts at these conditions. To assess the effect of temperature (T) on these coefficients, we use an Arrhenius law:

$$D = A \exp\left(\frac{-Q}{RT}\right) \tag{S10}$$

where A is the pre-exponential factor, Q is the activation enthalpy, and R is the universal gas constant. Posner et al, 2017 [43] showed that the activation energy Q for oxygen is nearly constant at 50 kJ/mol, while it increases significantly with pressure for Fe, from 50 kJ/mol up to 125 kJ/mol at 125 GPa. This implies that at 330 GPa, the activation energy for Fe reaches 248 kJ/mol.

At 330 GPa, the ratio of diffusion coefficients between 11,200 K and 5,200 K is calculated as follows:

$$\ln\left(\frac{D_{11200}}{D_{5200}}\right) = \frac{Q}{R}\left(\frac{1}{5200} - \frac{1}{11200}\right) \tag{S11}$$

Which gives:

$$D_{11200} = D_{5200} \times \exp\left[\frac{Q}{R}\left(\frac{1}{5200} - \frac{1}{11200}\right)\right] \tag{S12}$$

Using $Q_{Fe} = 248$ kJ/mol, we find D_{11200}(Fe) $= 12.5 \times 10^{-8}$ m^2/s. For oxygen, with $Q_O = 50$ kJ/mol, we find D_{11200}(O) $= 2.8 \times 10^{-8}$ m^2/s. To find the average distance x that atoms travel in $t = 10$ or 6 ns, we use the root-mean-square displacement along a line:

$$x = \sqrt{2Dt} \tag{S13}$$

At 330 GPa and 11,200 K oxygen can travel 23 nm and iron 50 nm within 10 ns (and 18 nm for oxygen and 39 nm for iron within 6 ns). Given that the initial grain size is smaller than 10 nm, atoms at the centre of a grain need only to travel a maximum of 5 nm to mix. Since the calculated distances significantly exceed this value, we can confidently attribute the melt signal to a single homogeneous Fe + 4.5 FeO melt.

[1] R. L. Blake, R. E. Hessevick, T. Zoltai, and L. W. Finger. Refinement of the Hematite Structure. *American Mineralogist*, 51:123–129, 1966.

[2] C. A. McCammon and Lin gun Liu. The effects of pressure and temperature on nonstoichiometric wüstite, FexO: The iron-rich phase boundary. *Physics and Chemistry of Minerals*, 10:106–113, 1984.

[3] Svein Stolen and Fredrik Gronvold. Calculation of the phase boundaries of wüstite at high pressure. *Journal of Geophysical Research*, 101:11531–11540, 1996.

[4] C.T. Seagle, D.L. Heinz, A.J. Campbell, V.B. Prakapenka, and S.T. Wanless. Melting and thermal expansion in the Fe-FeO system at high pressure. *Earth and Planetary Science Letters*, 265:655–665, 2008.

[5] Robert M. Hazen and Raymond Jeanloz. Wüstite (fe1-x o): A review of its defect structure and physical properties. *Reviews of Geophysics*, 22:37–46, 1984.

[6] R. A. Fischer, A. J. Campbell, G. A. Shofner, O. T. Lord, P. Dera, and V. B. Prakapenka. Equation of state and phase diagram of feo. *Earth and Planetary Science Letters*, 304:496–502, 2011.

[7] Raymond Jeanloz and Thomas J. Ahrens. Equations of state of FeO and CaO. *Geophysical Journal International*, 62(3):505–528, 1980.

[8] A. Descamps, T. M. Hutchinson, R. Briggs, E. E. McBride, M. Millot, T. Michelat, J. H. Eggert, B. Albertazzi, L. Antonelli, M. R. Armstrong, C. Baehtz, O. B. Ball, S. Banerjee, A. B. Belonoshko, A. Benuzzi-Mounaix, C. A. Bolme, V. Bouffetier, K. Buakor, T. Butcher, V. Cerantola, J. Chantel, A. L. Coleman, J. Collier, G. Collins, A. J. Comley, F. Coppari, T. E. Cowan, C. Crépisson, G. Cristoforetti, H. Cynn, S. Di Dio Cafiso, F. Dorchies, M. J. Duff, A. Dwivedi, D. Errandonea, E. Galtier, H. Ginestet, L. Gizzi, A. Gleason, S. Goede, J. M. Gonzalez, M. G. Gorman, M. Harmand, N. J. Hartley, P. G. Heighway, C. Hernandez-Gomez, A. Higginbotham, H. Höppner, R. J. Husband, H. Hwang, J. Kim, P. Koester, Z. Konopkova, D. Kraus, A. Krygier, L. Labate, A. Laso Garcia, A. E. Lazicki, Y. Lee, P. Mason, M. Masruri, B. Massani, D. McGonegle, C. McGuire, J. D. McHardy, R. S. McWilliams, S. Merkel, G. Morard, B. Nagler, M. Nakatsutsumi, K. Nguyen-Cong, A.-M. Norton, I. I. Oleynik, C. Otzen, N. Ozaki, S. Pandolfi, D. J. Peake, A. Pelka, K. A. Pereira, J. P. Phillips, C. Prescher, T. R. Preston, L. Randolph, D. Ranjan, A. Ravasio, R. Redmer, J. Rips, D. Santamaria-Perez, D. J. Savage, M. Schoelmerich, J-P. Schwinkendorf, S. Singh, J. Smith, R. F. Smith, A. Sollier, J. Spear, C. Spindloe, M. Stevenson, C. Strohm, T-A. Suer, M. Tang, T. Tschentscher, M. Toncian, T. Toncian, S. J. Tracy, M. Tyldesley, C. E. Vennari, T. Vinci, T. J. Volz, J. Vorberger, J. P. S. Walsh, J. S. Wark, J. T. Willman, L. Wollenweber, U. Zastrau, E. Brambrink, K. Appel, and M. I. McMahon. Calibration and characterization of the line-visar diagnostic at the HED-HIBEF instrument at the european XFEL. *Rev. Sci. Instrum.*, 96:075206, 2025.

[9] K. Katagiri, N. Ozaki, D. Murayama, K. Nonaka, Y. Hironaka, Y. Inubushi, K. Miyanishi, H. Nakamura, T. Okuchi, T. Sano, Y. Seto, K. Shigemori, K. Sueda, T. Togashi, Y. Umeda, M. Yabashi, T. Yabuuchi, and R. Kodama. Hugoniot equation-of-state and structure of laser-shocked polyimide $C_{22}H_{10}N_2O_5$. *Physical Review B*, 105:054103, 2022.

[10] Y. B. Zeldovitch and Y. P. Raizer. Shock waves in solids, in Physics of shock waves and high-temperature hydrodynamic phenomena. *Elsevier*, pages 685–784, 1967.

[11] Surinder M. Sharma, Stefan J. Turneaure, J. M. Winey, and Y. M. Gupta. Transformation of shock-compressed copper to the body-centered-cubic structure at 180 GPa. *Physycal Review B*, 102:020103(R), 2020.

[12] M. G. Gorman, D. McGonegle, R. F. Smith, S. Singh, T. Jenkins, R. S. McWilliams, B. Albertazzi, S. J. Ali, L. Antonelli, M. R. Armstrong, C. Baehtz, O. B. Ball, S. Banerjee, A. B. Belonoshko, A. Benuzzi-Mounaix, C. A. Bolme, V. Bouffetier, R. Briggs, K. Buakor, T. Butcher, S. Di Dio Cafiso, V. Cerantola, J. Chantel, A. Di Cicco, S. Clarke, A. L. Coleman, J. Collier, G. W. Collins, A. J. Comley, F. Coppari, T. E. Cowan, G. Cristoforetti, H. Cynn, A. Descamps, F. Dorchies, M. J. Duff, A. Dwivedi, C. Edwards, J. H. Eggert, D. Errandonea, G. Fiquet, E. Galtier, A. Laso Garcia, H. Ginestet, L. Gizzi, A. Gleason, S. Goede, J. M. Gonzalez, M. Harmand, N. J. Hartley, P. G. Heighway, C. Hernandez-Gomez, A. Higginbotham, H. Höppner, R. J. Husband, T. M. Hutchinson, H. Hwang, A. E. Lazicki, D. Keen, J. Kim, P. Koester, Z. Konopkova, D. Kraus, A. Krygier, L. Labate, Y. Lee, H.-P. Liermann, P. Mason, M. Masruri, B. Massani, E. E. McBride, C. McGuire, J. D. McHardy, S. Merkel, G. Morard, B. Nagler, M. Nakatsutsumi, K. Nguyen-Cong, A.-M. Norton, I. I. Oleynik, C. Otzen, N. Ozaki, S. Pandolfi, D. J. Peake, A. Pelka, K. A. Pereira, J. P. Phillips, C. Prescher, T. R. Preston, L. Randolph, D. Ranjan, A. Ravasio, R. Redmer, J. Rips, D. Santamaria-Perez, D. J. Savage, M. Schoelmerich, J.-P. Schwinkendorf, J. Smith, A. Sollier, J. Spear, C. Spindloe, M. Stevenson, C. Strohm, T.-A. Suer, M. Tang, M. Toncian, T. Toncian, S. J. Tracy, A. Trapananti, T. Tschentscher, M. Tyldesley, C. E. Vennari, T. Vinci, S. C. Vogel, T. J. Volz, J. Vorberger, J. P. S. Walsh, J. S. Wark, J. T. Willman, L. Wollenweber, U. Zastrau, E. Brambrink, K. Appel, and M. I. McMahon. Coordination changes in liquid tin under shock compression determined using in situ femtosecond x-ray diffraction. *Journal of Applied Physics*, 135:165902, 2024.

[13] R. Ramis, J. Meyer ter Vehn, and J. Ramírez. Multi2d – a computer code for two-dimensional radiation hydrodynamics. *Computer Physics Communications*, 180:977–994, 2009.

[14] J. Barnes and S. Lyon. SESAME: the Los Alamos National Laboratory equation of state database. *Los Alamos National Laboratory*, 1987.

[15] U. Zastrau, K. Appel, C. Baehtz, O. Baehr, L. Batchelor, A. Berghauser, M. Banjafar, E. Brambrink, V. Cerantola, T.E. Cowan, H. Damker, S. Dietrich, S. Di Dio Cafiso, J. Dreyer, H-O. Engel, T. Feldmann, S. Findeisen, M. Foese, D. Fulla-Marsa, S. Gode, M. Hassan, J. Hauser, T. Herrmannsdorfer, H. Hoppner, J. Kaa, P. Kaever, K. Knofel, Z. Konopkova, A. Laso Garcia, H-P. Liermann, J. Mainberger, M. Makita, E-C. Martens, E. E. McBride, D. Moller, M. Nakatsutsumi, A. Pelka, C. Plueckthun, C. Prescher, T. R. Preston, M. Roper, A. Schmidt, W. Seidel, J-P. Schwinkendorf, M. O.

Schoelmerichch, U. Schramm, A. Schropp, C. Strohm, K. Sukharnikov, P. Talkovski, I. Thorpe, M. Toncian, T. Toncian, L. Wollenweber, Sh. Yamamoto, and T. Tschentscher. The High Energy Density Scientific Instrument at the European XFEL. *Journal of Synchrotron Radiation*, 28(5):1393–1416, 2021.

[16] N. Ozaki, K. A. Tanaka, Y. Sasatani, K. Fujita, K. Takamatsu, M. Nakano, M. Yoshida, K. Okada, E. Takahashi, Y. Owadano, H. Takenaka, and K. Kondo. Hugoniot measurements for polyimide with laser and explosives. *Phys. Plasmas*, 10:2475–2479, 2003.

[17] J. S. Wark, D. J. Peake, T. Stevens, P. G. Heighway, Y. Ping, P. Sterne, B. Albertazzi, S. J. Ali, L. Antonelli, M. R. Armstrong, C. Baehtz, O. B. Ball, S. Banerjee, A. B. Belonoshko, C. A. Bolme, V. Bouffetier, R. Briggs, K. Buakor, T. Butcher, S. Di Dio Cafiso, V. Cerantola, J. Chantel, A. Di Cicco, A. L. Coleman, J. Collier, G. Collins, A. J. Comley, F. Coppari, T. E. Cowan, G. Cristoforetti, H. Cynn, A. Descamps, F. Dorchies, M. J. Duff, A. Dwivedi, C. Edwards, J. H. Eggert, D. Errandonea, G. Fiquet, E. Galtier, A. Laso Garcia, H. Ginestet, L. Gizzi, A. Gleason, S. Goede, J. M. Gonzalez, M. G. Gorman, M. Harmand, N. Hartley, C. Hernandez-Gomez, A. Higginbotham, H. Höppner, O. S. Humphries, R. J. Husband, T. M. Hutchinson, H. Hwang, D. Keen, J. Kim, P. Koester, Z. Konopkova, D. Kraus, A. Krygier, L. Labate, A. E. Lazicki, Y. Lee, H.-P. Liermann, P. Mason, M. Masruri, B. Massani, E. E. McBride, C. McGuire, J. D. McHardy, D. McGonegle, R. S. McWilliams, S. Merkel, G. Morard, B. Nagler, M. Nakatsutsumi, K. Nguyen-Cong, A.-M. Norton, I. I. Oleynik, C. Otzen, N. Ozaki, S. Pandolfi, A. Pelka, K. A. Pereira, J. P. Phillips, C. Prescher, T. R. Preston, L. Randolph, D. Ranjan, A. Ravasio, R. Redmer, J. Rips, D. Santamaria-Perez, D. J. Savage, M. Schoelmerich, J.-P. Schwinkendorf, S. Singh, J. Smith, R. F. Smith, A. Sollier, J. Spear, C. Spindloe, M. Stevenson, C. Strohm, T.-A. Suer, M. Tang, M. Toncian, T. Toncian, S. J. Tracy, A. Trapananti, T. Tschentscher, M. Tyldesley, C. E. Vennari, T. Vinci, S. C. Voge, T. J. Volz, J. Vorberger, J. T. Willman, L. Wollenweber, U. Zastrau, E. Brambrink, K. Appel, and M. I. McMahon. Femtosecond temperature measurements of laser-shocked copper deduced from the intensity of the x-ray thermal diffuse scattering. *Journal of Applied Physics*, 137(155904), 2025.

[18] W. J. Nellis, A. C. Mitchell, and A. K. McMahan. Carbon at pressures in the range 0.1–1 TPa (10 Mbar). *J. Appl. Phys.*, 90:696–698, 2001.

[19] Zhiyu He, Melanie Rödel, Julian Lütgert, Armin Bergermann, Mandy Bethkenhagen, Deniza Chekrygina, Thomas E. Cowan, Adrien Descamps, Martin French, Eric Galtier, Arianna E. Gleason, Griffin D. Glenn, Siegfried H. Glenzer, Yuichi Inubushi, Nicholas J. Hartley, Jean-Alexis Hernandez, Benjamin Heuser, Oliver S. Humphries, Nobuki Kamimura, Kento Katagiri, Dimitri Khaghani, Hae Ja Lee, Emma E. McBride, Kohei Miyanishi, Bob Nagler, Benjamin Ofori-Okai, Norimasa Ozaki, Silvia Pandolfi, Chongbing Qu, Divyanshu Ranjan, Ronald Redmer, Christopher Schoenwaelder, Anja K. Schuster, Michael G. Stevenson, Keiichi Sueda, Tadashi Togashi, Tommaso Vinci, Katja Voigt, Jan Vorberger, Makina Yabashi, Toshinori Yabuuchi, Lisa M. V. Zinta, Alessandra Ravasio, and Dominik Kraus. Diamond formation kinetics in shock-compressed cho samples recorded by small-angle x-ray scattering and x-ray diffraction. *Science Advances*, 8, 2022.

[20] Ben Heuser, Armin Bergermann, Michael G. Stevenson, Divyanshu Ranjan, Zhiyu He, Julian Lütgert, Samuel Schumacher, Mandy Bethkenhagen, Adrien Descamps, Eric Galtier, Arianna E. Gleason, Dimitri Khaghani, Griffin D. Glenn, Eric F. Cunningham, Siegfried H. Glenzer, Nicholas J. Hartley, Jean-Alexis Hernandez, Oliver S. Humphries, Kento Katagiri, Hae Ja Lee, Emma E. McBride, Kohei Miyanishi, Bob Nagler, Benjamin Ofori-Okai, Norimasa Ozaki, Silvia Pandolfi, Chongbing Qu, Philipp Thomas May, Ronald Redmer, Christopher Schoenwaelder, Keiichi Sueda, Toshinori Yabuuchi, Makina Yabashi, Bratislav Lukic, Alexander Rack, Lisa M. V. Zinta, Tommaso Vinci, Alessandra Benuzzi-Mounaix, Alessandra Ravasio, and Dominik Kraus. Release dynamics of nanodiamonds created by laser-driven shock-compression of polyethylene terephthalate. *Scientific Reports*, 14, 2024.

[21] Yanbin Wang, Tatsuya Sakamaki, Lawrie B. Skinner, Zhicheng Jing, Tony Yu, Yoshio Kono, Changyong Park, Guoyin Shen, Mark L. Rivers, and Stephen R. Sutton. Atomistic insight into viscosity and density of silicate melts under pressure. *Nature Communications*, 5, 2014.

[22] J. Lütgert, J. Vorberger, N. Hartley, K. Voigt, M. Rödel, A. Schuster, A. Benuzzi-Mounaix, S. Brown, T. Cowan, E. Cunningham, T. Döppner, R. W. Falcone, L. B. Fletcher, E. Galtier, S. H. Glenzer, A. L. Garcia, D. O. Gericke, P. A. Heimann, H. J. Lee, E. E. McBride, A. Pelka, I. Prencipe, A. M. Saunders, M. Schölmerich, M. Schörner, P. Sun, T. Vinci, A. Ravasio, and D. Kraus. Measuring the structure and equation of state of polyethylene terephthalate at megabar pressures. *Scientific Reports*, 11, 2021.

[23] N. Nettelmann, N. Movshovitz, D. Ni, J. J. Fortney, E. Galanti, Y. Kaspi, R. Helled, C. R. Mankovich, and S. Bolton. Theory of figures to the seventh order and the interiors of jupiter and saturn. *The Planetary Science Journal*, 2, 2021.

[24] Bertrand Guillot and Nicolas Sator. A computer simulation study of natural silicate melts. part II: High pressure properties. *Geochimica et Cosmochimica Acta*, 71:4538–4556, 2007.

[25] Stefan J. Turneaure, Surinder M. Sharma, and Y. M. Gupta. Crystal structure and melting of fe shock compressed to 273 GPa: In situ x-ray diffraction. *Phys. Rev. Lett.*, 125:215702, 2020.

[26] S. Singh, R. Briggs, M. G. Gorman, L. X. Benedict, C. J. Wu, S. Hamel, A. L. Coleman, F. Coppari, A. Fernandez-Pañella, C. McGuire, M. Sims, J. K. Wicks, J. H. Eggert, D. E. Fratanduono, and R. F. Smith. Structural study of hcp and liquid iron under shock compression up to 275 GPa. *Physical Review B*, 108:184104, 2023.

[27] A. Amouretti, C. Crepisson, S. Azadi, D. Cabaret, T. Campbell, D. A. Chin, B. Colin, G. R. Collins, L. Crandall, G. Fiquet, A. Forte, T. Gawne, F. Guyot, P. Heighway, H. Lee, D. McGonegle, B. Nagler, J. Pintor, D. Polsin, G. Rousse, Y. Shi, E. Smith, J. S. Wark, S. M. Vinko, and M. Harmand. Phase transitions of Fe_2O_3 under laser shock compression. *Physycal Review Letters*, 134:176102, 2025.

[28] E. Bykova, L. Dubrovinsky, N. Dubrovinskaia, M. Bykov, C. McCammon, S. V. Ovsyannikov, H-P. Liermann, I. Kupenko, A. I. Chumakov, R. Rüffer, M. Hanfland, and V. Prakapenka. Structural complexity of simple Fe_2O_3 at high pressures and temperatures. *Nature Communications*, 7(1):10661, 2016.

[29] Andrew Higginbotham and David McGonegle. Prediction of debye-scherrer diffraction patterns in arbitrarily strained samples. *J. Appl. Phys.*, 115:174906, 2014.

[30] Benedict J. Heinen and James W. E. Drewitt. Liquiddiffract: Software for liquid total scattering analysis. *Physics and Chemistry of Minerals*, 49(9):1432–2021, 2022.

[31] F. Hadju. Revised parameters of the analytic fits for coherent and incoherent scattered x-ray intensities of the first 36 atoms. *Acta Crystallographica Section A*, A28:250–252, 1972.

[32] Roy Kaplow, S. L. Strong, and B. L. Averbach. Radial density functions for liquid mercury and lead. *Physical Review*, 138, 1965.

[33] Jon H. Eggert, Gunnar Weck, Paul Loubeyre, and Mohamed Mezouar. Quantitative structure factor and density measurements of high-pressure fluids in diamond anvil cells by x-ray diffraction: Argon and water. *Physical Review B*, 65:174105, 2002.

[34] C. Sanloup, F. Guyot, P. Gillet, G. Fiquet, R. J. Hemley, M. Mezouar, and I. Martinez. Structural changes in liquid Fe at high pressures and high temperatures from synchrotron x-ray diffraction. *Europhysics Letters*, 52(2):151–157, 2000.

[35] L. Gonzalez and D. Gonzalez. Structure and dynamics in liquid iron at high pressure and temperature. a first principles study. *Journal of Geophysical Research: Solid Earth*, 128:e2022JB025119, 2023.

[36] Benedict J. Heinen and James W. E. Drewitt. LiquidDiffract: software for liquid total scattering analysis. *Physics and Chemistry of Minerals*, 49(9), 2022.

[37] James W. E. Drewitt. Liquid structure under extreme conditions: high-pressure x-ray diffraction studies. *Journal of Physics: Condensed Matter*, 33:503004, 2005.

[38] Guillaume Morard, Jean-Alexis Hernandez, Clara Pege, Charlotte Nagy, Lélia Libon, Antoine Lacquement, Dimosthenis Sokaras, Hae Ja Lee, Eric Galtier, Philip Heimann, Eric Cunningham, Siegfried H. Glenzer, Tommaso Vinci, Clemens Prescher, Silvia Boccato, Julien Chantel, Sébastien Merkel, Yanyao Zhang, Hong Yang, Xuehui Wei, Silvia Pandolfi, Wendy L. Mao, Arianna E. Gleason, Sang Heon Shim, Roberto Alonso-Mori, and Alessandra Ravasio. Structural evolution of liquid silicates under conditions in Super-Earth interiors. *Nature Communications*, 15:8483, 2024.

[39] D. Kraus, J. Rips, M. Schörner, M. G. Stevenson, J. Vorberger, D. Ranjan, J. Lutgert, B. Heuser, J. H. Eggert, H.-P. Liermann, I. I. Oleynik, S. Pandolfi, R. Redmer, A. Sollier, C. Strohm, T. J. Volz, B. Albertazzi, S. J. Ali, L. Antonelli, C. Bähtz, O. B. Ball, S. Banerjee, A. B. Belonoshko, C. A. Bolme, V. Bouffetier, R. Briggs, K. Buakor, T. Butcher, V. Cerantola, J. Chantel, A. L. Coleman, J. Collier, G. W. Collins, A. J. Comley, T. E. Cowan, G. Cristoforetti, H. Cynn, A. Descamps, A. Di Cicco, S. Di Dio Cafiso, F. Dorchies, M. J. Duff, A. Dwivedi, C. Edwards, D. Errandonea, S. Galitskiy, E. Galtier, H. Ginestet, L. Gizzi, A. Gleason, S. Göde, J. M. Gonzalez, M. G. Gorman, M. Harmand, N. J. Hartley, P. G. Heighway, C. Hernandez-Gomez, A. Higginbotham, H. Höppner, R. J. Husband, T. M. Hutchinson, H. Hwang, D. Keen, J. Kim, P. Koester, Z. Konôpková, A. Krygier, L. Labate, A. Laso Garcia, A. E. Lazicki, Y. Lee, P. Mason, M. Masruri, B. Massani, E. E. McBride, J. D. McHardy, D. McGonegle, C. McGuire, R. S. McWilliams, S. Merkel, G. Morard, B. Nagler, M. Nakatsutsumi, K. Nguyen-Cong, A.-M. Norton, N. Ozaki, C. Otzen, D. J. Peake, A. Pelka, K. A. Pereira, J. P. Phillips, C. Prescher, T. R. Preston, L. Randolph, A. Ravasio, D. Santamaria-Perez, D. J. Savage, M. Schölmerich, J.-P. Schwinkendorf, S. Singh, J. Smith, R. F. Smith, J. Spear, C. Spindloe, T.-A. Suer, M. Tang, M. Toncian, T. Toncian, S. J. Tracy, A. Trapananti, C. E. Vennari, T. Vinci, M. Tyldesley, S. C. Vogel, J. P. S. Walsh, J. S. Wark, J. T. Willman, L. Wollenweber, U. Zastrau, E. Brambrink, K. Appel, and M. I. McMahon. The structure of liquid carbon elucidated by in situ x-ray diffraction. *Nature*, 642:351–354, 2025.

[40] Adam F. Sapnik, Philip A. Chater, Dean S. Keeble, John S. O. Evans, Federica Bertolotti, Antonietta Guagliardi, Lise J. Støckler, Elodie A. Harbourne, Anders B. Borup, Rebecca S. Silberg, Adrien Descamps, Clemens Prescher, Benjamin D. Klee, Axel Phelipeau, Imran Ullah, Karel G. Medina, Tobias A. Bird, Viktoria Kaznelson, William Lynn, Andrew L. Goodwin, Bo B. Iversen, Celine Crepisson, Emil S. Bozin, Kirsten M. Jensen, Emma E. McBride, Reinhard B. Neder, Ian Robinson, Justin Wark, Michal Andrzejewski, Ulrike Boesenberg, Erik Brambrink, Carolina Camarda, Valerio Cerantola, Sebastian Goede, Hauke Höppner, Oliver S. Humphries, Zuzana Konopkova, Naresh Kujala, Thomas Michelat, Motoaki Nakatsutsumi, Thomas R. Preston, Lisa Randolph, Andreas Schmidt, Cornelius Strohm, Minxue Tang, Ulf Zastrau, Karen Appel, and David Keen. High-quality ultra-fast total scattering and pair distribution function data using an x-ray free electron laser. *IUCrJ*, 12:531–547, 2025.

[41] J. M. Brown, J. N. Fritz, and R. S. Hixson. Hugoniot data for iron. *J. Appl. Phys.*, 88:5496–5498, 2000.

[42] L. X. Benedict, R. G. Kraus, S. Hamel, and J. L. Belof. A limited-ranged two-phase iron equation of state model. *LLNL Technical Report*, 2022.

[43] Esther S. Posner, Gerd Steinle-Neumann, Vojtěch Vlček, and David C. Rubie. Structural changes and anomalous self-diffusion of oxygen in liquid iron at high pressure. *Geophysical Research Letters*, 44:3526–3534, 2017.

[44] Monica Pozzo, Chris Davies, David Gubbins, and Dario Alfe. Transport properties for liquid silicon-oxygen-iron mixtures at earth's core conditions. *Phys. Rev. B*, 87:014110, 2013.
