# Supplementary material for: Structural evolution of iron oxides melts at Earth’s outer-core pressures
Source: Nat Commun. 2026 Jul 10;17:8527. doi: 10.1038/s41467-026-75204-4 (PMC13482581; doi:10.1038/s41467-026-75204-4)
Supplement: Supplementary file 2 [file 41467_2026_75204_MOESM2_ESM.pdf]

Description of the Additional Supplementary Files

Supplementary Data 1: Summary of all individual data points used in this work including laser conditions, probing times, pressures, phases present, cell parameters for solid, and shock fraction for liquid.

Supplementary Data 2: Summary of liquid diffraction analysis including pressure and temperature for all data points, density, coordination number and interatomic distance.

Supplementary Data 3: Electron Microprobe Analysis for Fe_2O_3 and Fe+4.5FeO sample.

Supplementary Data 4: Structure factor and pair distribution functions calculated in this study.
