# Supplementary material for: Structural evolution of iron oxides melts at Earth’s outer-core pressures
Source: Nat Commun. 2026 Jul 10;17:8527. doi: 10.1038/s41467-026-75204-4 (PMC13482581; doi:10.1038/s41467-026-75204-4)
Supplement: Supplementary file 3 [file 41467_2026_75204_MOESM7_ESM.pdf]

nature portfolio

Peer Review File

Structural evolution of iron oxides melts at Earth's outer-core pressure

Corresponding Author: Dr Céline Crépisson

This file contains all reviewer reports in order by version, followed by all author rebuttals in order by version.

Version 0:

Reviewer comments:

Reviewer #1

(Remarks to the Author)
In this study, Crepisson et al. leverage the unprecedented peak brilliance and temporal resolution of the European XFEL to investigate the liquid phase of various iron oxides under extreme conditions. By integrating ultra-fast in situ X-ray diffraction with laser-driven shock compression, the authors effectively bypass the kinetic limitations associated with traditional static compression. This approach allows for the real-time characterization of Fe-O bonding environments at megabar pressures and temperatures of several thousand Kelvin.
Such structural analysis is pivotal for mapping the pressure-temperature-induced evolution of the liquid phase diagram, specifically regarding how coordination changes in iron oxides influence melt density and viscosity. As these data are essential for refining models of Earth's outer core and clarifying the transport properties of these melts, the study potentially provides a more robust framework for understanding the geodynamo—the convective process within the liquid metal core responsible for generating and sustaining the terrestrial magnetic field.
The authors provide an extensive body of supporting material and a rigorous methodological framework, demonstrating a commendable effort to account for potential experimental artifacts. This is particularly evident in the sections regarding background subtraction and the extraction of the pair distribution function, g(r). However, while the technical execution is robust, certain aspects require further clarification and improvement to fully support the authors' claims.
A primary concern—a recurring point of contention in shock compression experiments—is the insufficient discussion of temperature. Throughout the manuscript, the authors present XRD patterns indicating melting or phase transitions, yet they consistently report only the pressure. It is essential to include temperature estimates in the main text and to provide a detailed explanation in the supplementary materials regarding how these temperatures were derived.
Furthermore, while a phase diagram including the Hugoniot is provided in the supporting information, the general reader would benefit significantly from its inclusion in the main text. This would allow for a clearer comparison between the observed structural evolution and existing literature, specifically regarding the Hugoniot path used by the authors.
Another critical omission is the lack of structural refinement for the observed phases. While I recognize the focus is on the molten state for density extraction, the authors should provide refinements for ambient conditions and for points where "new" features emerge. This is necessary to interpret the variety of peaks visible in the reported figures. For instance, in some samples claimed to be molten, several peaks remain visible—or new feature appearing in Figure 3 at 180 GPa—which require a formal explanation.
Finally, for a publication in a high-impact journal such as Nature Communications, the manuscript lacks a deep discussion on the implications of these findings. To strengthen the work, the authors should expand on how their results specifically enhance our understanding of the current geodynamo and the broader geophysical significance of their observations.
In addition to the primary concerns mentioned above, several other technical issues throughout the manuscript require clarification.
i) While the experimental setup in Figure 1 is informative, the inclusion of the raw XRD signal on the detector is largely unnecessary and could be removed to streamline the visualization.
ii) Regarding data quality, there is a noticeable discrepancy in Figure 3, where the signal appears significantly noisier than in the other figures. The authors should provide a rationale for this difference.
iii) In Figure 4, the distinction between the patterns at 84 GPa and 147 GPa remains unclear, as both exhibit a broad diffuse feature between 2 and 6 A-1. Notably, the 147 GPa pattern appears more structured than the one at 84 GPa, yet it is labeled as amorphous. The authors must clarify the criteria used to differentiate these phases and explain how they distinguished the amorphous state from the liquid phase.
iv) Concerning the extraction and interpretation of the pair distribution function, g(r), it is well established in PDF analysis

that the Q-range is a critical determinant of real-space resolution. It is therefore unclear why a larger Q-range was utilized for pure Fe—a relatively simpler system—than for the more complex iron oxides where higher resolution is typically required.
v) Regarding the data collected on the Varex detectors, two aspects are particularly concerning: first, in Figure S12, the observed peak shift in Varex 1 appears only in the shocked sample and is absent in the unshocked patterns at 33 GPa, where the dashed lines indicate peaks that are entirely unaffected. Second, the image in Figure S13 shows that the signal in Varex 1 is tilted relative to Varex 2. The authors should identify the cause of this misalignment and discuss how it impacts the data integration and subsequent interpretation.
vi) Finally, in Figure S27, the authors compare signals collected at the XFEL with those from the Diamond Light Source (notably, the beamline is I15, not ID15). Beyond the fact that the signals were clearly extracted using different protocols—as evidenced by the noise profiles surrounding the first peak—it is striking that structural information above 7 A seems entirely lost in the XFEL data. The authors should provide an explanation for this loss of signal and clarify whether the same sample was used for both measurements, as well as detailing the specific PDF collection parameters employed at DLS.

Reviewer #2

(Remarks to the Author)
The behavior of two iron oxides compositions under high pressure is explored using shock compression combined with in-situ x-ray diffraction. Conditions are relevant to Earth's interior, hence results are used to discuss implications on the planet's core structure and composition. In particular, differences in the iron the iron first shell coordination number are used to argue of a possible mechanism of chemical stratification in the outer core.

The paper is very well organized, and written. Data are presented in clear graphics. The analysis of the raw data appears to be thorough.

The aspect I think should be improved is the discussion of temperatures in your experiments and obviously, of the impact temperature differences between Earth's core and experiments entail in the implications discussed. In this regard, the last sentence of the abstract can be a bit deceiving, looking at pressure only as the property characterizing a depth. It needs to be made cleared to the reader in the paper what temperature can be associated to a certain shock pressure, provide the whole range is not sufficient. Fig. S28 should be moved to the main paper.

Rather than assumed, the FeO composition could be estimated from unit cell parameters, if you can, provide such estimate.

Overall the paper is excellent, and while in attempting to explore the unaccessible strong assumptions are unavoidable, there is a benefit in presenting them upfront. The observations made remain precious and worth publication.

Reviewer #3

(Remarks to the Author)
This manuscript by Crepisson and others reports laser-shock experiments with in-situ XFEL measurements to understand the structure of the outer core, which is thought to consist of liquid Fe–O.. Recent activities in laser shock compression and XFEL measurements are the main streams of high-pressure studies to explore the internal structure of Earth and planets. This study reveals that the coordination number of the Fe is four in the Fe-O melt at extreme pressure up to 438 GPa. I consider this a novel finding. However, as is often the case in recent shock experiments, the implications for Earth and the planets are somewhat unclear. Although I believe this manuscript is potentially suitable for publication in Nature Communications, the authors need to further strengthen its implications for Earth and the planets.

Implications:
The conclusions are not sufficiently compelling for the broad readership of Nature Communications. While the experimental data are novel, their significance for Earth science is not fully convincing. The authors determine the density and coordination number of liquid Fe–O under core conditions; however, these findings do not substantially revise the conventional view of the Earth's core. The current conclusion that "the incorporation of oxygen decreases the density of the liquid iron (Fig. 7)" is repeatedly reported by experiments and theoretical calculations.
The authors emphasise that the Fe–O coordination number is lower in liquid iron than in silicate melts. Although this appears to be a new observation, the manuscript does not clearly demonstrate how the coordination number is directly related to geophysical observations such as seismic anomalies or the geodynamo. In its current form, the broader geophysical implications remain insufficiently developed.

Valence state:
The manuscript highlights the role of iron valence state in generating heterogeneity in the outer core (p. 8, upper right column). The discussion does not sound reasonable.
"These structural differences between Fe^{3+}-rich and Fe^{2+}-rich melts thus persist under extreme pressures and temperatures. Such compositional contrasts could contribute to lateral heterogeneities near the top of the Earth's outer-core, …."
The presence of Fe^{3+} in liquid iron is unlikely, given the core's low oxygen content. Although ionic and metallic liquids can coexist in the Fe–O system at relatively low pressures (<25 GPa; Frost et al., 2010), even if an ionic Fe–O liquid were present in the core, it would most likely be dominated by Fe^{2+}. The oxygen content of the core is estimated to be below ~10 wt% (Hirose et al., 2013), significantly lower than the stoichiometric composition of $Fe^{2+}O$.

“…where regions of the lower mantle may be relatively enriched in Fe^{3+} [54, 55]”
This is the case of the solid lower mantle as shown in the references [54, 55]. The lower mantle likely contains a large amount of Fe^{3+}, but it is not melted. This is not linked with the experimental results. The authors may discuss here the chemical reaction between the lower mantle and the core, but the solid lower mantle may not chemically react with the liquid outer core to a great extent because of the low diffusion rate in the solid (e.g., Holzapfel et al., 2005).

Sample:
The Fe + FeO sample consists of intergrown Fe + FeO (B1) crystals prepared by plasma sputtering (Methods and Fig. S2). As mentioned in the manuscript, the melting is a nanosecond phenomenon; however, the chemical diffusion of the elements can be much slower. This raises the concern that the liquid may not be chemically homogeneous during the ~10 nanosecond laser pulse (Fig S5). This should be fully discussed.
Other methods appear robust, including laser-shock experiments, XFEL measurements and XRD data analysis.

Version 1:

Reviewer comments:

Reviewer #1

(Remarks to the Author)
I would like to thank the authors for their diligent efforts in addressing the previous round of reviewer comments. The revised manuscript has improved in several areas. However, certain critical technical ambiguities remain. In particular, the temperature characterization and the structural discrimination between disordered phases require more rigorous treatment to meet the publication standards of Nature Communications.
Specific Comments
1. Quantitative Uncertainty in Temperature Measurements
While I appreciate the additional details regarding the experimental challenges of temperature measurement in shock experiments, the current discussion lacks a quantitative assessment of uncertainty. The authors state that temperatures cannot be directly extracted from the Rankine-Hugoniot relations, yet, Figure 1 displays points across a vast range (4,000–15,000 K) without temperature error bars. Given that part of their discussion e.g. the distinction between the amorphous solid and liquid phases relies on the assertion that "T is too low" for melting, a robust error analysis is essential. The authors should provide at least a semi-quantitative estimate of the uncertainty associated with their temperature assumptions (e.g., using SESAME EOS or similar models) to substantiate their phase assignments.
2. Clarity on Multi-state Probing in X-ray Diffraction (XRD)
The authors note that the XRD patterns likely reflect a superposition of multiple states of matter sampled simultaneously during the shock. This is a critical point for the interpretation of the data. Given the broad, interdisciplinary readership of Nature Communications, even though this is a “standard practice”, this nuance must be explicitly stated and discussed in the main text to ensure the findings are accessible to those outside the specialized XFEL community.
3. Le Bail Analysis and Comparative Data
Regarding the Le Bail refinement of the observed features: the authors argue that similar features were observed in their previous work. However, the existence of prior observations does not preclude the need for a comprehensive structural discussion in the current manuscript. I request that the authors include a figure comparing the current data with previous results or provide a representative Le Bail fit to justify their structural assignments.
4. Data Justification in the Absence of VISAR Diagnostics
I have concerns regarding the inclusion of data points at 82 and 147 GPa (Figure 4) given the authors' admission that no VISAR data was retrieved for these runs. Without velocimetry to constrain the pressure-temperature conditions, it is unclear how these points can be reliably interpreted or compared to the rest of the dataset. The authors should clarify the diagnostic basis for including these specific points.
5. Discrimination Between Liquid and Amorphous Solid Phases
The structural discrimination between the liquid and amorphous solid states remains a point of concern. In a true liquid, the Pair Distribution Function should show a rapid decay of oscillations beyond the first coordination shell due to the loss of medium-range order. Conversely, an amorphous solid—while lacking long-range periodicity—retains a "frozen" rigid network that typically manifests as sharper, more persistent peaks at higher r-values. I invite the authors to provide a more detailed analysis of the PDF peak widths and the evolution of the second and third coordination shells to support their claim of an amorphous solid state.

Reviewer #3

(Remarks to the Author)
All my concerns have been properly addressed, and I am now able to recommend this manuscript for publication.

Version 2:

Reviewer comments:

Reviewer #1

(Remarks to the Author)

The authors have addressed all my concerns. I am happy for this work to be published in Nature Communications.

Open Access This Peer Review File is licensed under a Creative Commons Attribution 4.0 International License, which permits use, sharing, adaptation, distribution and reproduction in any medium or format, as long as you give appropriate credit to the original author(s) and the source, provide a link to the Creative Commons license, and indicate if changes were made.
In cases where reviewers are anonymous, credit should be given to 'Anonymous Referee' and the source.
The images or other third party material in this Peer Review File are included in the article's Creative Commons license, unless indicated otherwise in a credit line to the material. If material is not included in the article's Creative Commons license and your intended use is not permitted by statutory regulation or exceeds the permitted use, you will need to obtain permission directly from the copyright holder.
To view a copy of this license, visit https://creativecommons.org/licenses/by/4.0/

We thank the reviewers for their relevant and highly useful comments. We significantly improve the manuscript by discussing in more details the temperature of our experimental data points, and the implications of our findings. We also performed new Scanning Transmission Electron Microscope (STEM) imaging of the Fe+4.5 FeO sample to determine the grain size of the sample and thus clarify the nature of the melt observed on this sample under shock following the comment of reviewer #3. We added one author: Phani S. Karamched who performed the STEM and added the signed form regarding change to the author list.

A detailed answer to the reviews is provided below

REVIEWER COMMENTS

Reviewer #1 (Remarks to the Author):

In this study, Crepisson et al. leverage the unprecedented peak brilliance and temporal resolution of the European XFEL to investigate the liquid phase of various iron oxides under extreme conditions. By integrating ultra-fast in situ X-ray diffraction with laser-driven shock compression, the authors effectively bypass the kinetic limitations associated with traditional static compression. This approach allows for the real-time characterization of Fe-O bonding environments at megabar pressures and temperatures of several thousand Kelvin.
Such structural analysis is pivotal for mapping the pressure-temperature-induced evolution of the liquid phase diagram, specifically regarding how coordination changes in iron oxides influence melt density and viscosity. As these data are essential for refining models of Earth's outer core and clarifying the transport properties of these melts, the study potentially provides a more robust framework for understanding the geodynamo—the convective process within the liquid metal core responsible for generating and sustaining the terrestrial magnetic field.
The authors provide an extensive body of supporting material and a rigorous methodological framework, demonstrating a commendable effort to account for potential experimental artifacts. This is particularly evident in the sections regarding background subtraction and the extraction of the pair distribution function, g(r). However, while the technical execution is robust, certain aspects require further clarification and improvement to fully support the authors' claims.

We thank the reviewer for his careful reading of the paper, and the emphasis on the importance of our study for refining models of Earth's outer-core, as well as the emphasis on its rigorous approach.

A primary concern—a recurring point of contention in shock compression experiments—is the insufficient discussion of temperature. Throughout the manuscript, the authors present XRD patterns indicating melting or phase transitions, yet they consistently report only the pressure. It is essential to include temperature estimates in the main text and to provide a detailed explanation in the supplementary materials regarding how these temperatures were derived.
Furthermore, while a phase diagram including the Hugoniot is provided in the supporting information, the general reader would benefit significantly from its inclusion in the main text. This would allow for a clearer comparison between the observed structural evolution and existing literature, specifically regarding the Hugoniot path used by the authors.

We agree about the point underlined by the reviewer, and appreciate that to be fully comprehensive to the reader and valuable for Earth Sciences, shock data should discuss temperature evaluation. Temperature measurements during shock compression experiments remain scarce: streak optical pyrometry (SOP), now available at HED, EuXFEL, was not calibrated at the time of our experiment. Thermal diffuse scattering has been developed within our group although this approach has so far only been used on cubic crystal and monoatomic minerals such as copper (Wark et al., JAP, 2025). Moreover, this technique is not suitable for amorphous or liquid materials. Alternatively, EXAFS measurement can allow access to temperature with the help of ab-initio MD calculations (e.g. Sio et al., 2023 Nature Comm.).

Shock temperatures cannot be extracted directly from the Rankine-Hugoniot relations and approximations are needed to evaluate temperature along the Hugoniot. For these reasons there is still a large uncertainty on temperature estimates along the Hugoniot of various materials.

We added the figure below (slightly modified from previous figure S28 in SM) to the main paper to show further the temperature of our data points compared to the Earth geotherm as also recommended by the reviewer #2. We added to the figure the previous DFT+U calculated Hugoniot for Fe_2O_3 up to 115 GPa using phases retrieved under laser-driven shock compression (Crepisson et al., PRB 2025).

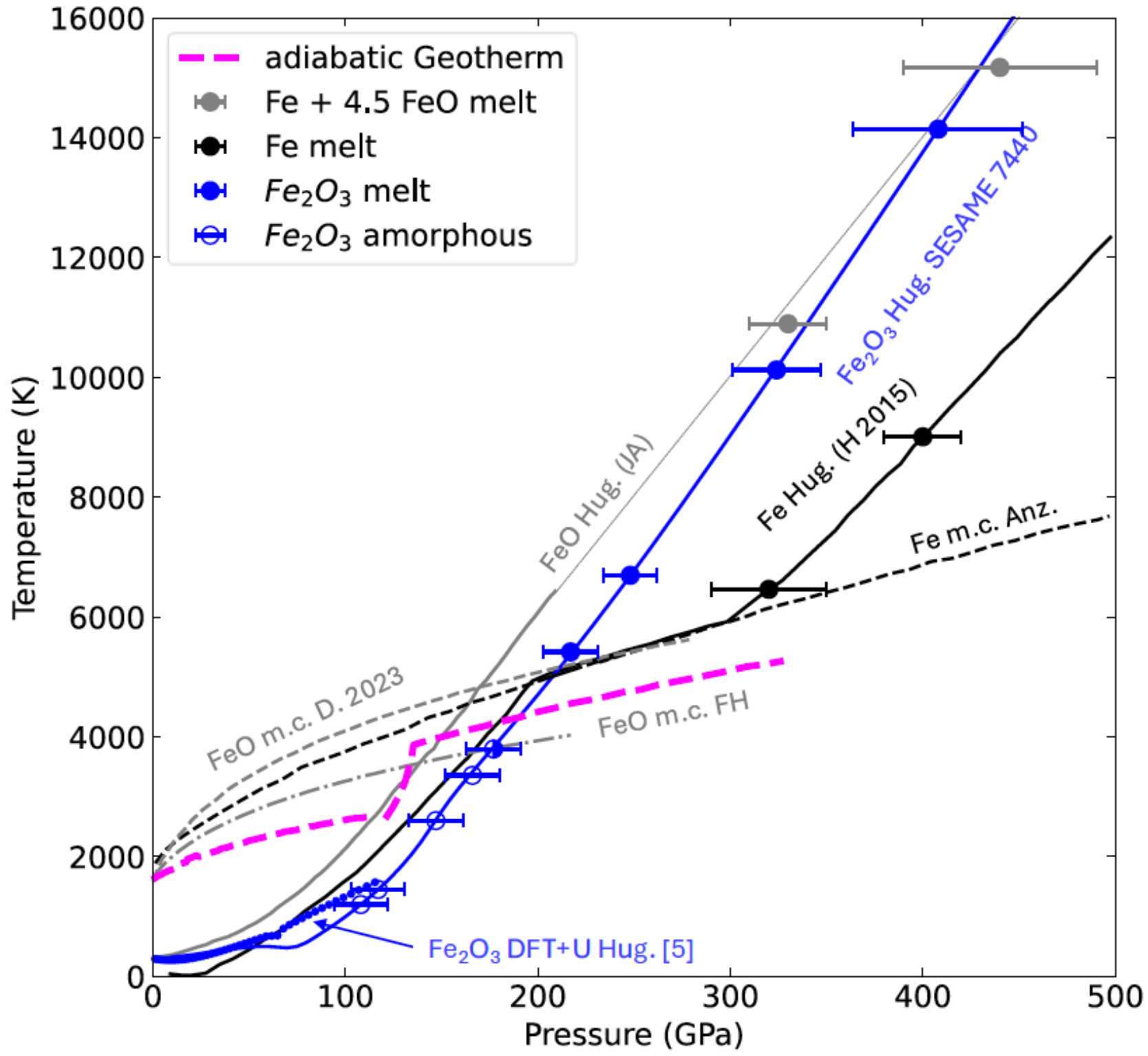

Figure 1: Pressure–temperature conditions of our data compared with the geotherm [1–3]. Temperatures are estimated from SESAME 7440 [4], consistent with previous DFT+U calculations [5]. The FeO Hugoniot (dashed grey line) is linearly extrapolated from Jeanloz and Ahrens [6] and the Fe Hugoniot from DFT calculations [7]. Melting curves are shown for Fe [8] and FeO [9, 10]. The half-filled Fe_2O_3 data point at 177(14)GPa is either liquid or amorphous.

We also added the following text to the main paper (p2, middle column right) to define our temperature range and the temperature estimate and how it locates compared to the Geotherm.

Shock temperature could not be directly measured in our case and approximations are needed to evaluate temperature along the Hugoniot. In this work we used previously calculated temperatures along the Hugoniot curve of Fe [7], FeO [6] and Fe_2O_3 (SESAME 7440 [4] and DFT+U [5]). Temperatures are shown to range between 4,000–15,000 K, as shown in Fig. 1. We see that the Fe_2O_3 data points at 166(14), 177(14), and 217(14) GPa, and the Fe data point at 320(30) GPa, have a temperature within 1,000 K of the Geotherm while other data points are significantly hotter.

We discussed the effect of temperature within the revised manuscript and changes can be found in response to reviewer#3 in points **A** and **B.**

Another critical omission is the lack of structural refinement for the observed phases. While I recognize the focus is on the molten state for density extraction, the authors should provide refinements for ambient conditions and for points where "new" features emerge. This is necessary to interpret the variety of peaks visible in the reported figures. For instance, in some samples claimed to be molten, several peaks remain visible—or new feature appearing in Figure 3 at 180 GPa—which require a formal explanation.

Our measurements were timed to probe the sample when the shock front had traversed most of the target but a small unshocked portion remained, thereby minimizing the ambient-phase contribution to the diffraction signal while ensuring the measurement was made under shock compression rather than during release. The resulting XRD signal is therefore dominated by the shocked phase, with a minor residual contribution from the unshocked material. The relative proportion of ambient signal depends on timing precision relative to shock breakout. This approach is now standard practice at XFEL facilities coupled to laser-driven shock compression and is described in detail elsewhere (e.g. Harmand et al., PRB 92 2015; Tracy et al., PRB 99 2019); we therefore do not elaborate further here.

All timings are given in Table 1 of the supplementary material, **and we added the probed time to breakout time,** so that the reader can better evaluate the relative part of remaining ambient: the longer the probed time to breakout the more ambient will be seen.

Ambient peaks therefore, persist even in fully molten XRD signals, as seen in Figures 3, 4, and 5, and are indicated by vertical dashed lines corresponding to the 1D lineout of the ambient sample. Figures 4 and 5 additionally show the position of the Al(111) peak, arising from a thin (200-400 nm) aluminium layer deposited on the Fe_2O_3 and Fe+4.5 FeO samples. The apparent increase in ambient peak intensity at 200(20) GPa relative to 190(20) GPa for Fe+4.5 FeO does not reflect any new structural feature, it is a timing artifact. The sample was probed 0.2 ns before shock breakout at 190(20) GPa and 0.4 ns before breakout at 200(20) GPa, leaving a larger unshocked fraction in the latter case, assuming comparable shock velocities at the two pressures. No new feature attributable to the high-pressure phase appears at 200(20) GPa.

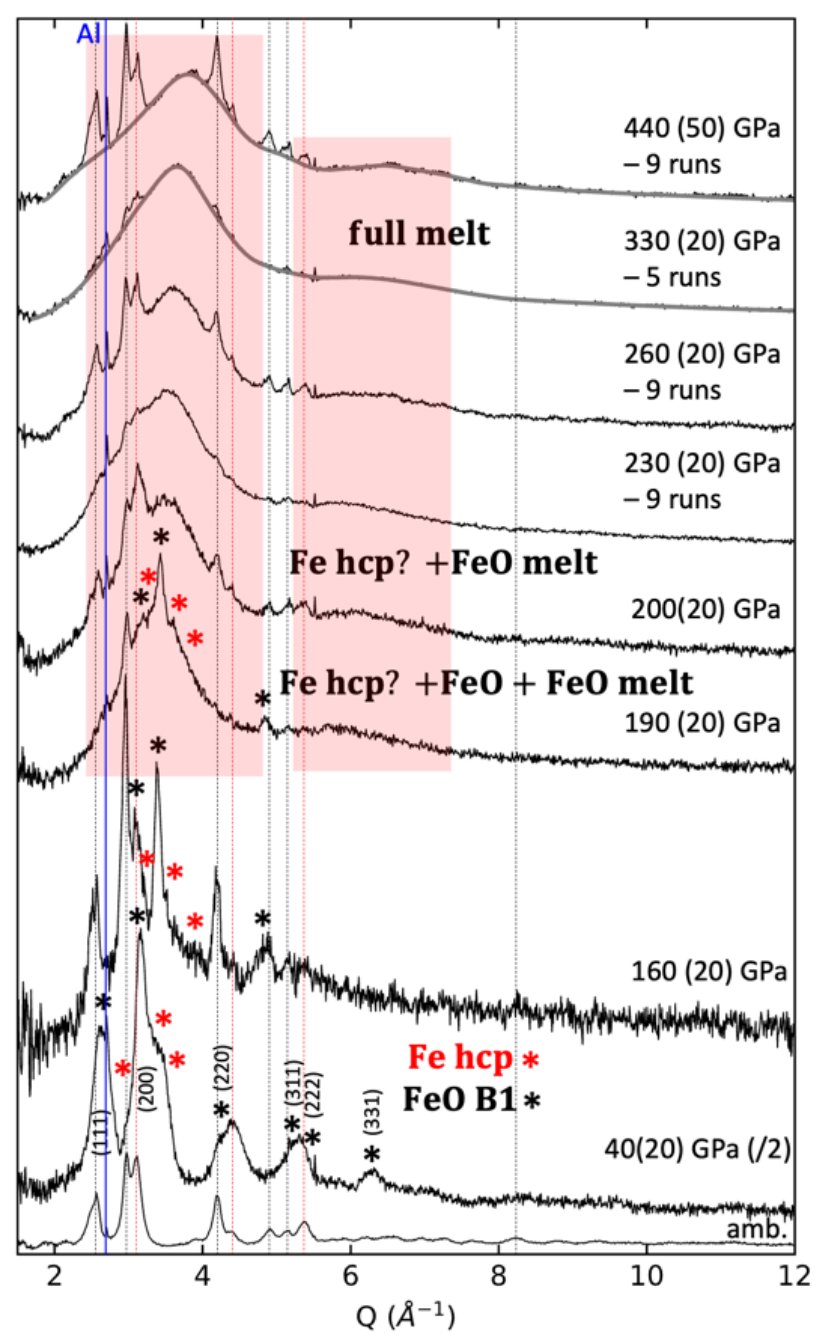

We added in fig. 4 and 5 the main Al peak visible (111) and in the caption:

while the Al (111) peak due to the Al flash coating is marked in blue.

Le Bail refinement was performed on Fe_2O_3 phases under shock in a previous work (Amouretti et al., 2025 PRL): in this work we evidenced similar alpha and alpha prime phases and indexed those phases in figure 4 and in the supplementary material in section VIII, IX and X. As no new feature was observed compared to previous work we did not dedicate a full study on this point.

No phase transition is observed for FeO and we are thus only indexing the FeO (rocksalt structure) B1 peaks along with Fe.

Finally, for a publication in a high-impact journal such as Nature Communications, the manuscript lacks a deep discussion on the implications of these findings. To strengthen the work, the authors should expand on how their results specifically enhance our understanding of the current geodynamo and the broader geophysical significance of their observations.

We agree with the reviewer, and we significantly extended our discussion on the implications of our findings, as detailed in the response to reviewer#3 in points **C and D.**

In addition to the primary concerns mentioned above, several other technical issues throughout the manuscript require clarification.
i) While the experimental setup in Figure 1 is informative, the inclusion of the raw XRD signal on the detector is largely unnecessary and could be removed to streamline the visualization.

We would like to keep the display of the detectors as it emphasizes their widths and position which might be difficult to visualize otherwise for the reader.

ii) Regarding data quality, there is a noticeable discrepancy in Figure 3, where the signal appears significantly noisier than in the other figures. The authors should provide a rationale for this difference.

In our experiment we performed single-shot measurement: each shot is taken on a different target, which is destroyed during the measurement. Each shot is probed with a given XFEL pulse of around 50 fs duration, XFEL pulse in SASE mode have an intensity varying from shot to shot (e.g. Bermudez Macias et al., Optics Express 29, 2021), which explain general variation in XRD intensity for a given type of target, and thus in signal over noise ratio (SNR). As detailed in the Methods we renormalized the XRD signal to the XFEL pulse intensity (section IV of the SM and mentioned in the Methods), however the effect remains on the SNR:

SNR of our dataset varies in function of three parameters:

-natural fluctuation of the XFEL pulse as described above

-average Z-number of the material (Fe = 26 , Fe+4.5FeO = 17.9 , Fe_2O_3 = 15.2)

-thickness of the target (Fe = 20 µm foil, Fe_2O_3 = 20 µm deposition, Fe+4.5 FeO = 10 µm deposition)

That is why Fe has a significantly better SNR as it has a higher Z and is thick. Fe_2O_3 has a lower Z but is twice thicker than the Fe+4.5FeO sample. Natural variation of XFEL pulse in SASE mode accounts for the different SNR within a given type of target.

We added an explanation in the Methods in section C:

All corrections described above were applied to each individual dataset prior to summation. Identical XRD signals from multiple runs were then combined to enhance the signal-to-noise ratio, a step crucial for reliable liquid diffraction analysis.
The signal-to-noise ratio (SNR) varies as a function of sample thickness and average atomic number. Consequently, the Fe dataset exhibits an overall higher SNR than the Fe_2O_3 and Fe + 4.5 FeO datasets. Similarly, Fe_2O_3 has a higher SNR than Fe + 4.5 FeO because it is significantly thicker. Variations in the SNR within a single sample are linked to the natural fluctuations of XFEL pulse intensity in SASE mode.

iii) In Figure 4, the distinction between the patterns at 84 GPa and 147 GPa remains unclear, as both exhibit a broad diffuse feature between 2 and 6 A-1. Notably, the 147 GPa pattern appears more structured than the one at 84 GPa, yet it is labeled as amorphous.

We assume that the reviewer wanted to mention the difference between 82(14) and 147(14) GPa.

At 82(14) GPa, alpha prime Fe_2O_3 peaks are clearly seen and indexed on figure 4, and in figure S14 and S18 of the SM. This signal differs from the signal observed at 147(14) GPa as we can see in Fig. S18 of the SM. Indeed, at 147(14) GPa only 2 peaks are seen (identified by black stars in figure 4 and S18), which could be possibly attributed to the remaining alpha prime phase as discussed in the main article and in the SM. All other peaks present are remaining ambient peaks indicated by dashed lines. The difference in SNR between measurement at 82(14) GPa and 147(14) GPa is possibly confusing as the "more structured patterns" that the reviewer refers to, seems mostly linked to the remaining ambient material, which is more present in the measurement at 147(14) GPa. Unfortunately, for those runs at 82 and 147 GPa no VISAR was retrieved (as shown in the Table 1) so we cannot give the probed time to breakout for comparison.

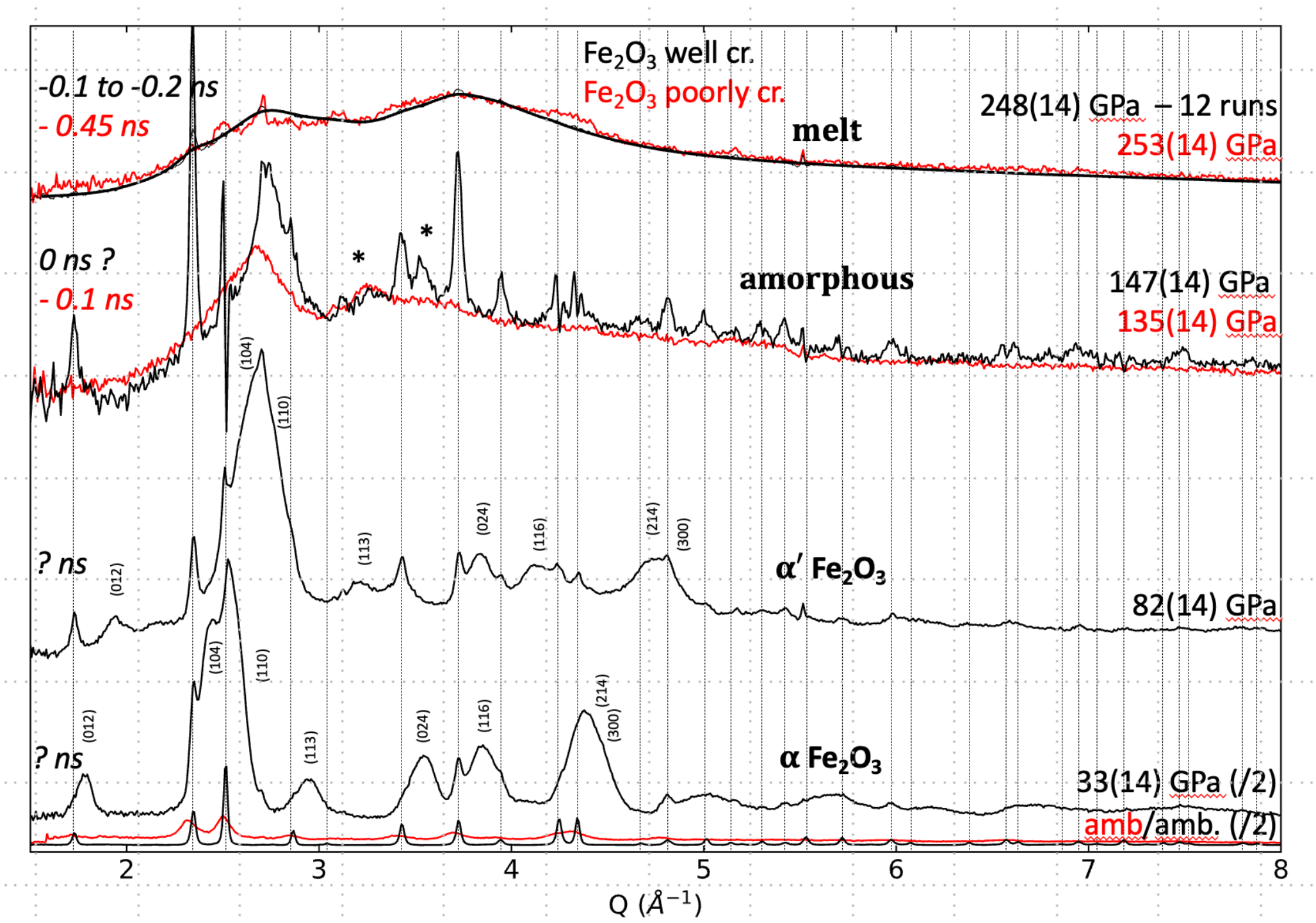

Figure S18

The authors must clarify the criteria used to differentiate these phases and explain how they distinguished the amorphous state from the liquid phase.

Determining the difference between amorphous and melt via x-ray diffraction is difficult: a simple observation of an x-ray diffraction signal cannot decide between an amorphous and a molten phase. In our work we bring further discussion on the distinction between amorphous and melt via 3 main arguments:

1/the temperature is too low at 110 GPa to be explained by a melt as we detailed in Results, section A, bottom p3:

This is consistent with an earlier study, where amorphization occurred at 122(3) GPa (Crepisson et al., PRB), at temperatures too low to induce equilibrium melting (1200 K at 108 GPa, according to SESAME 7440.

2/the position of the main amorphous peak is not moving but start to broaden and decrease upon melting:

The position of the main amorphous peak at 2.74Å-1 remains constant from 108(14) to 166(14) GPa, consistent with previous observations for amorphous Fe_2O_3 (Crepisson et al., PRB). Between 166(14) and 177(14) GPa, this peak broadens and decreases markedly in intensity, accompanied by the disappearance of all Bragg reflections. This behaviour indicates that the Fe_2O_3 Hugoniot crosses the solidus or liquidus near this pressure.

3/we use two different samples to distinguish between amorphous and melt signature as shown in Fig. 5:

Given the difficulty to distinguish between amorphous and melt via XRD we modified the text and remove the "clear distinction" (p4, bottom) which might be too positive as we are here more using a set of arguments to propose a distinction.

The comparison of two Fe_2O_3 samples of identical composition but differing initial crystallinity (Fig. 4) ***allows us to distinguish between amorphous and molten phases****. At 135–147(14) GPa, the samples display distinct diffraction signatures, consistent with an amorphous or partially amorphous phase that retains features of the original crystallinity. In contrast, at 248–253(14) GPa, both samples exhibit nearly identical signals, indicative of complete melting. We thus conclude that Fe_2O_3 is amorphous at 147(14) GPa, and likely also at 166(14) GPa, as the corresponding patterns are similar (see Fig. 4 and section X of the Supplemental Material for more details). From 217(14) GPa onwards Fe_2O_3 is fully molten [XRD signatures at 217(14) and 248(14) GPa being nearly identical].*

iv) Concerning the extraction and interpretation of the pair distribution function, g(r), it is well established in PDF analysis that the Q-range is a critical determinant of real-space resolution. It is therefore unclear why a larger Q-range was utilized for pure Fe—a relatively simpler system—than for the more complex iron oxides where higher resolution is typically required.

The data were collected in a same experiment, therefore the technical available Q-range is always the same (~ 12 $Å^{-1}$) as can be seen from the figures 3,4,5. Nevertheless one can see that the signal over noise ratio (SNR) is far better for Fe, due to higher Z and thick sample than for Fe+4.5FeO and Fe_2O_3. That is why we had to cut the structure factor to around 9 $Å^{-1}$ instead of 12 $Å^{-1}$ for liquid Fe in link with poor SNR after 10 $Å^{-1.}$

In the SM figs. S20, S21, S22 we present all smoothing and cutting of the structure factor. However, we only present the data till 10 $Å^{-1}$ as data is not used beyond the cut-off. Indeed as can be seen in the figure below for the structure factor of Fe_2O_3 at 177(14) GPa the ending of the signal after 9 $Å^{-1}$ would lead to artifact in the pair distribution function as the termination is not smooth toward the value of 1. Therefore we cut the signal after 9 $Å^{-1}$ and allow for a smooth termination to 1.

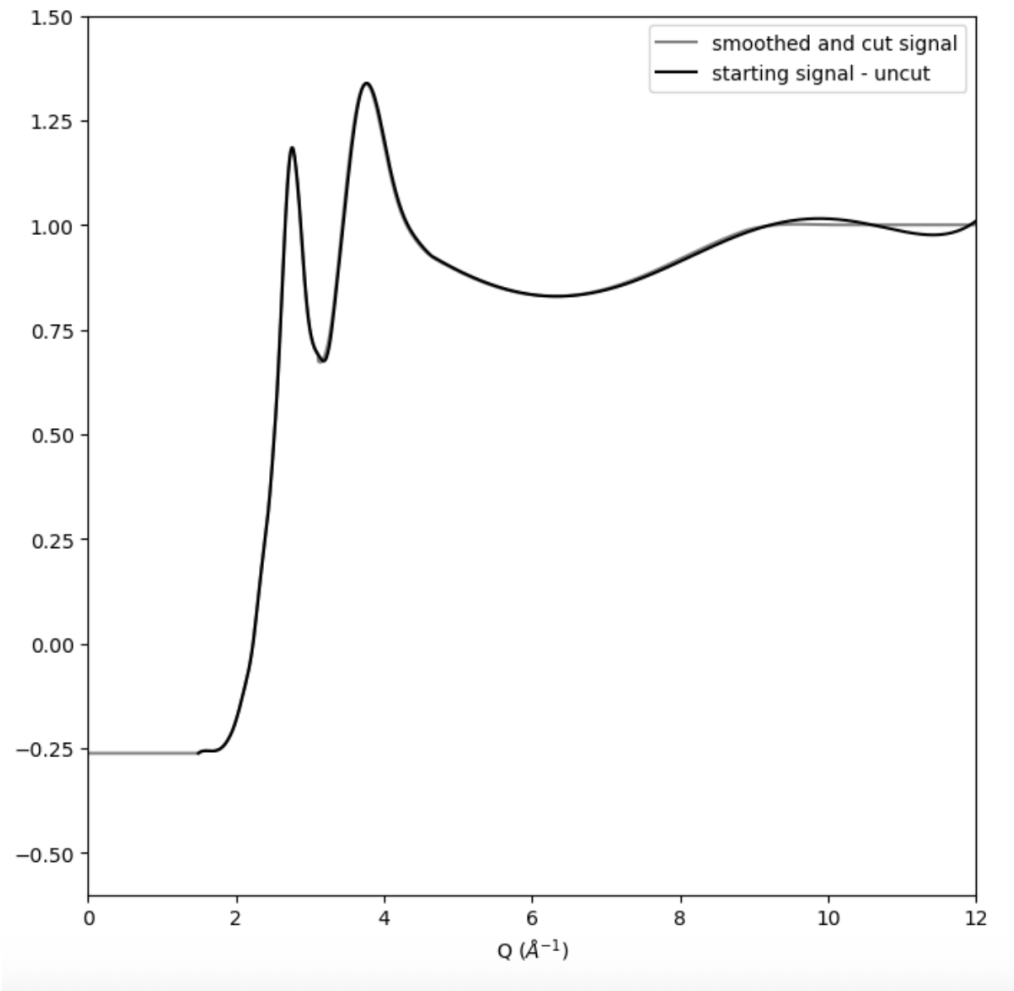

We added in the SM section XI (p20, bottom):

We determine a smooth baseline from the fully corrected XRD intensity as shown in figs. 2,3,4 of the main paper. Faber-Ziman structure factor is then calculated by using data point up to around 9 $Å^{-1}$ for iron oxide and 12 $Å^{-1}$ for Fe. ***Fe dataset has a higher signal-over-noise ratio in link with higher atomic number and thicker sample, which explain why we could use the data till larger Q range without producing an artefact in the pair distribution function.***

v) Regarding the data collected on the Varex detectors, two aspects are particularly concerning: first, in Figure S12, the observed peak shift in Varex 1 appears only in the shocked sample and is absent in the unshocked patterns at 33 GPa, where the dashed lines indicate peaks that are entirely unaffected. Second, the image in Figure S13 shows that the signal in Varex 1 is tilted relative to Varex 2. The authors should identify the cause of this misalignment and discuss how it impacts the data integration and subsequent interpretation.

In section VIII we detail data on solid Fe_2O_3: the bending observed on shocked sample and shown in section VIII of the SM is not linked to trouble in calibration:

Indeed, if we compare Bragg peak reflection position for both VAREX, positions should be similar, which is the case for ambient Fe_2O_3 sample within 0.007 $Å^{-1}$ as shown in figure S14, and as seen in the figure below that it is further the case for all ambient samples and for the CeO_2 calibrant:

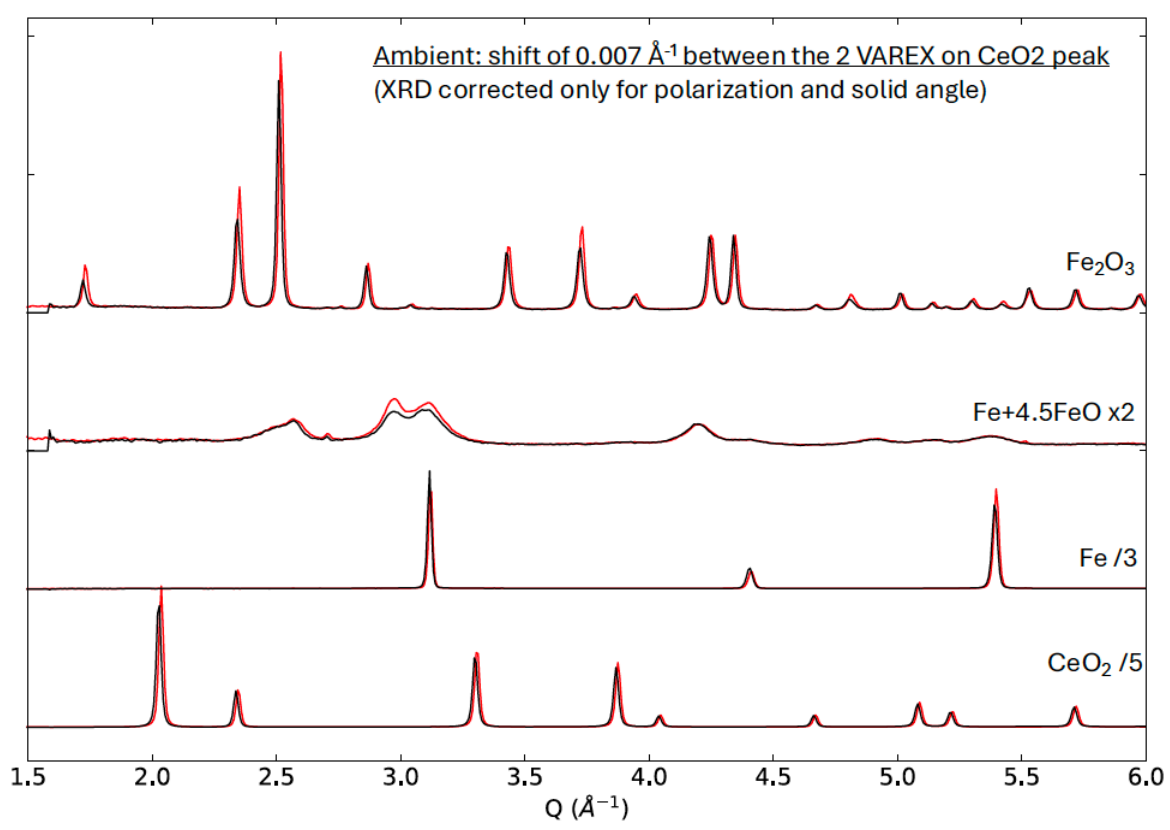

The shift in Bragg position along the azimuthal angle involves either an elastic precursor or a strength effect, which thus only occurs under uniaxial loading. This can be used to determine the strength of a material (Higginbotham and McGonegle, JAP, 115, 2014) although it is complexified in our case by

the texture of the sample, which means that the theoretical framework needs to be further adapted (cf. Foster et al.,JAP, 129, 24, 2021). We are planning to fully investigate this in a separate study although more experimental data might be required.

vi) Finally, in Figure S27, the authors compare signals collected at the XFEL with those from the Diamond Light Source (notably, the beamline is I15, not ID15). Beyond the fact that the signals were clearly extracted using different protocols—as evidenced by the noise profiles surrounding the first peak—it is striking that structural information above 7 A seems entirely lost in the XFEL data. The authors should provide an explanation for this loss of signal and clarify whether the same sample was used for both measurements, as well as detailing the specific PDF collection parameters employed at DLS.

We agree with the reviewer and clarified the collection parameters employed at DLS, similarly to the one found in Sapnik et al., 2025 paper (that we referenced in our paper): "data [were] obtained from four layers of the same glass measured on the I15-1 diffractometer at Diamond Light Source (data measured by Daniel Irving as part of the I15-1 mail-in service, proposal CY39017)."

We added in the main paper in Methods section A:

Finally a 25 µm commercial metallic glass from Goodfellow (Fe78B13Si9) ***previously measured at I15-1 diffractometer at Diamond Light Source (data measured by Daniel Irving as part of the I15-1 mail-in service, proposal CY39017) at 76 keV [59],*** *was used*

We then added in the SM section XIII (p29):

Metallic glass was measured on the exact same glass at synchrotron Diamond Light Source. Four layers of the glass were used on the I15-1 diffractometer at Diamond Light Source (data measured by Daniel Irving as part of the I15-1 mail-in service, proposal CY39017). It was run in transmission geometry with a 2D detector placed behind the sample and perpendicular to the beam in standard configuration at 76 keV.

The synchrotron and XFEL data were treated exactly the same way, with the same python routine we wrote. Nevertheless, when we mention cutting at 11 $Å^{-1}$ for the XFEL data and for the synchrotron data, we refer to the cut-off applied on the structure factor in the iterative procedure, so that the starting structure factor before the iterative procedure is longer than the XFEL data.

As this may be confusing we now directly cut the synchrotron data at around 12 $Å^{-1}$ to have a similar starting Q-range as the one available with the XFEL set-up, and we then work on a smooth termination and cut the signal with the modification function at 11 $Å^{-1}$ in the iterative procedure.

We can now see that we have close to the exact same pair distribution function for the XFEL data and the cut synchrotron data at 11 $Å^{-1}$ as shown below.

Similarly for the XFEL cut at 10 $Å^{-1}$ we cut the starting signal at 10 $Å^{-1}$ and further use the modification function to cut the structure factor at 10 $Å^{-1}$.

We thus modified the figure S29 as shown below, where we can now see that XFEL and synchrotron data give nearly the same pair distribution function:

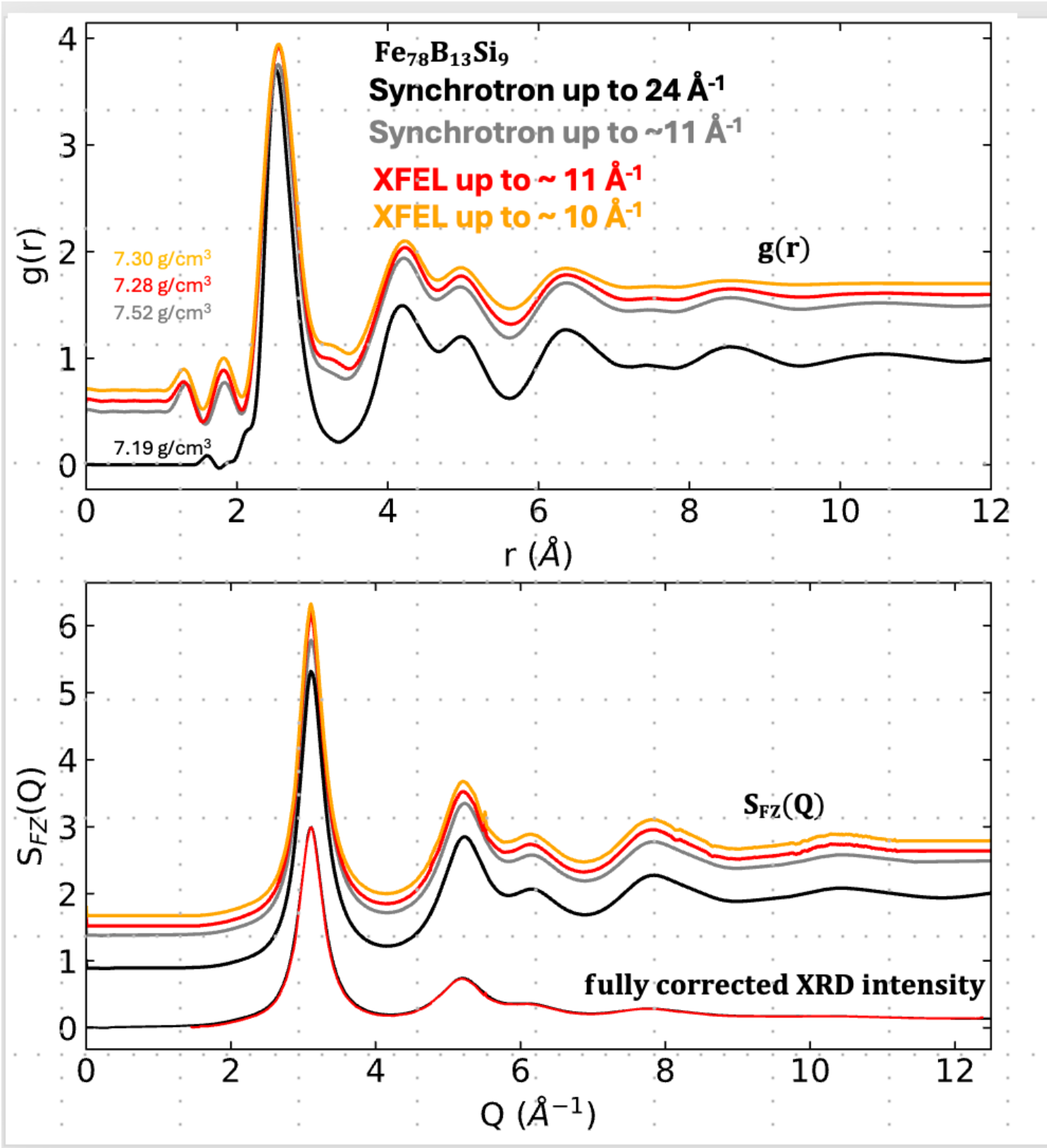

We modified the residual map for the synchrotron cut at 11 Å^{-1} (fig. S22)

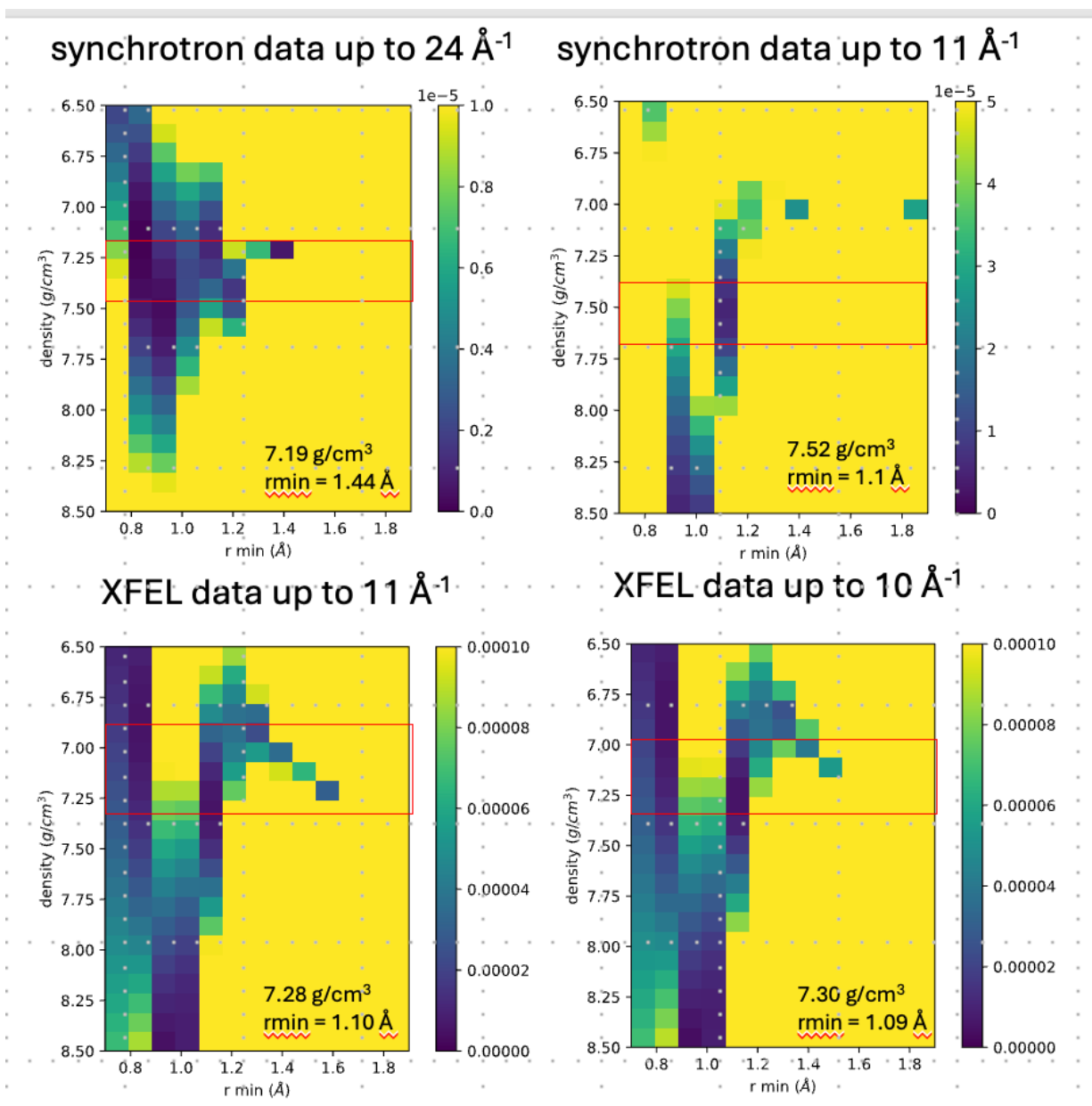

And we corrected the text below in SM section XIII (p29):

The difference between the full synchrotron measurement (up to 24 Å^{-1}) and the XFEL truncated at 10 Å^{-1} is 0.11 g/cm^3 (1.5%). *This is the same cut-off applied to the iron oxide data shown in Fig. S19.*

We note that the artificial truncation of the data can lead to difficulty in density determination as the termination is less smooth than when considering the entire signal and applying a modification function at the cut-off used for the calculation of the pair distribution function. This however only affects the present treatment of the metallic glass when we try to compare different set-up, and none of the other measurements where the objective is to keep the longer Q-range possible.

Reviewer #2 (Remarks to the Author):

The behavior of two iron oxides compositions under high pressure is explored using shock compression combined with in-situ x-ray diffraction. Conditions are relevant to Earth's interior, hence results are used to discuss implications on the planet's core structure and composition. In particular, differences in the iron the iron first shell coordination number are used to argue of a possible mechanism of chemical stratification in the outer core.

The paper is very well organized, and written. Data are presented in clear graphics. The analysis of the raw data appears to be thorough.

The aspect I think should be improved is the discussion of temperatures in your experiments and obviously, of the impact temperature differences between Earth's core and experiments entail in the implications discussed. In this regard, the last sentence of the abstract can be a bit deceiving, looking at pressure only as the property characterizing a depth. It needs to be made cleared to the reader in the paper what temperature can be associated to a certain shock pressure, provide the whole range is not sufficient. Fig. S28 should be moved to the main paper.

We added a slightly modified version of the previous figure S28 to the main paper (now figure 1 of the main paper) to show the best estimation of temperature for our data points. We were thus able to compare to the Earth geotherm as also recommended by the reviewer #1. We added to the graph the DFT+U Hugoniot calculated for Fe_2O_3.

We note that Fe_2O_3 data point at 177(14) and 217(14) GPa (as well as Fe data point at 330(20) GPa) are close to the geotherm as seen in figure 1. We added the temperature in the abstract:

At ~177 GPa (~380 km below the core-mantle boundary) ***and ~3,800 K,***

We discussed the effect of temperature within the revised manuscript and changes can be found in response to reviewer#3 in points **A and B.**

Rather than assumed, the FeO composition could be estimated from unit cell parameters, if you can, provide such estimate.

We improved our treatment of the Fe+4.5FeO sample, regarding consideration of the initial density and its effect on pressure as well as a discussion on stoichiometry. We added in Section I of the SM :

The exact $Fe_{1-x}O$ stoichiometry could not be determined because bcc-Fe and wüstite grains were intermingled at sub-micron length scales, smaller than the Electron MicroProbe Analysis (EMPA) interaction volume. The $Fe_{1-x}O$ sample is also showing an intrinsic residual stress from thin layer sputtering due to PVD. From preshot measurements and regions remaining at ambient conditions during driven shots, the initial molar volume was determined to be V_0 = 11.61(0.03) cm3.mol^{-1}. Owing to residual stress, the $Fe_{1-x}O$ lattice parameter cannot be used to infer stoichiometry via the relation of McCammon and Liu [2]. Nevertheless above 5 GPa, FeO is expected to be close to stoichiometric in presence of Fe excess [3, 4]. $Fe_{1-x}O$ is known to exist in composition ranging from $Fe_{0.88}O$ to $Fe_{0.96}O$ at ambient conditions [5]. Our sample is composed of Fe and $Fe_{1-x}O$. Volumes are determined by the position of the Bragg peak from XRD measurement. V_0 Fe is 7.148(0.05) cm3.mol^{-1} (average of 24 data points from preshot measurement or remaining ambient under shock

not all presented in this work but to be submitted separately) and V_0 $Fe_{1-x}O$ of 11.614(0.03) cm3.mol^{-1} (average of 32 data points from preshot measurement or remaining ambient under shock not all presented in this work but to be submitted separately). The initial volume found for $Fe_{1-x}O$ corresponds to a pressure of 8-11 GPa if we assume a bulk modulus of 141.5 or 185 GPa (taking the extreme value published so far [2, 6, 7]), a FeO composition, i.e. an expected volume of 12.252 cm3.mol^{-1} at ambient conditions [2] and using second order Birch-Murnaghan EOS. Therefore, it is possible that our sample is stoichiometric FeO at ambient condition due to this initial compression as it has been shown that above 5 GPa $Fe_{1-x}O$ in presence of Fe is stoichiometric FeO [3, 4]. Density of our sample can be calculated as follows

$$\rho_{\text{sample}} = \frac{(AM_{\text{Fe}} + BM_{\text{Fe}_{1-x}\text{O}})}{(AV_{\text{Fe}} + BV_{\text{Fe}_{1-x}\text{O}})}$$

with A and B the atomic proportions of Fe and $Fe_{1-x}O$ in the sample and MFe and M $Fe_{1-x}O$ the average molar mass and V_{Fe} and V $_{Fe1-xO}$ the measured volume in cm3.mol^{-1} detailed above. We know the Fe and O contents based on EMPA (55.0(0.4) at% Fe and 45.0(0.4) at% O: depending on the stoichiometry we can find A and B the proportion of Fe and $Fe_{1-x}O$. If we assume a stoichiometric FeO, we have A =1, and B = 4.5, while if we assume $Fe_{0.88}O$, we have A = 1.54 and B = 4.5. Thus density could at the maximum vary from 6.00 g.cm^{-3} for $Fe_{0.88}O$ composition to 6.38 g.cm^{-3} for stoichiometric FeO. For the highest pressure calculated (440(50) GPa) using an initial density of 6.38 g.cm^{-3}, if the density is changed to 6.00 g.cm^{-3}, this decreases the estimated pressure by around 30 GPa, while it only decreases the pressure by 10 GPa at 200(20) GPa. We thus note that the effect of the initial density on pressure estimate remains within previously determined error bars.

And in section III of the SM

As Fe + 4.5 FeO sample has a different initial density (ρsample) than EOS initial density used in the hydrodynamic simulation (see details in section I) and for impedance match (ρeos) we correct for this effect afterward following Rankine-Hugoniot equations:

$$P = \rho_0 U_s U_p$$

Thus:

$$P = P_{\text{hydro}} \frac{\rho_{\text{sample}}}{\rho_{\text{eos}}}$$

We thus modified the pressure accordingly in the entire manuscript (figs. 4, 6, 7, 8), in SM, (fit gaussian, map residual and SQ) and in the SM Table 1 and 2.

New pressures as displayed in fig. 4 of the main article are reestimated to: 40(20), 160(20), 190(20), 200(20), 230(20), 260(20), 330(20), 440(50) GPa

And we added in the sample description (p2 main, middle column right):

We report measurements of the melt structure and density of Fe, Fe + 4.5 FeO (55.0(0.4) at% Fe and 45.0(0.4) at% O, equivalent to Fe + 4.5 FeO in atomic proportion, assuming stoichiometric FeO composition, with Fe and FeO homogeneously distributed across the sample), and Fe_2O_3. ***Further details on initial samples are given in Supplemental Material, section I. We study pressures ranging (…)***

Overall the paper is excellent, and while in attempting to explore the unaccessible strong assumptions are unavoidable, there is a benefit in presenting them upfront. The observations made remain precious and worth publication.

Reviewer #3 (Remarks to the Author):

This manuscript by Crepisson and others reports laser-shock experiments with in-situ XFEL measurements to understand the structure of the outer core, which is thought to consist of liquid Fe–O.. Recent activities in laser shock compression and XFEL measurements are the main streams of high-pressure studies to explore the internal structure of Earth and planets. This study reveals that the coordination number of the Fe is four in the Fe-O melt at extreme pressure up to 438 GPa. I consider this a novel finding. However, as is often the case in recent shock experiments, the implications for Earth and the planets are somewhat unclear. Although I believe this manuscript is potentially suitable for publication in Nature Communications, the authors need to further strengthen its implications for Earth and the planets.

Implications:
The conclusions are not sufficiently compelling for the broad readership of Nature Communications. While the experimental data are novel, their significance for Earth science is not fully convincing. The authors determine the density and coordination number of liquid Fe–O under core conditions; however, these findings do not substantially revise the conventional view of the Earth's core. The current conclusion that "the incorporation of oxygen decreases the density of the liquid iron (Fig. 7)" is repeatedly reported by experiments and theoretical calculations.

While our density results are not leading to novel results, extraction of the density using liquid diffraction allows us to show that our data analysis is robust. Moreover, there are so far very few experimental density measurements along the Hugoniot at the pressure we investigate here. Our data are bringing new constraints on Fe and iron oxides Hugoniot curve that we think valuable to validate further theoretical calculations of Hugoniot and compare with the few previous existing experimental measurements at these conditions.

We added a description of the density results at the end of the Results (p7, top left) section to underline these points:

The extracted densities shown in Fig. 8 are in good overall agreement with previous theoretical and experimental data, validating our data analysis approach. For Fe, results agree with liquid diffraction measurements (Singh et al., 2023) and are slightly shifted toward higher density relative to the optical Hugoniot data of Brown et al. (Brown et al., 2022). Fe_2O_3 densities are consistent with the SESAME 7440 equation of state and previous optical Hugoniot measurements within uncertainty above 248(14) GPa. Discrepancies at lower pressures are discussed below. As expected, densities for Fe + 4.5 FeO fall between those of FeO (Jeanloz and Ahrens, 1980) and Fe with 9 wt% O (Young et al., 2021).

The authors emphasise that the Fe–O coordination number is lower in liquid iron than in silicate melts. Although this appears to be a new observation, the manuscript does not clearly demonstrate how the coordination number is directly related to geophysical observations such as seismic anomalies or the geodynamo. In its current form, the broader geophysical implications remain insufficiently developed.

Valence state:
The manuscript highlights the role of iron valence state in generating heterogeneity in the outer core (p. 8, upper right column). The discussion does not sound reasonable.

"These structural differences between Fe3+-rich and Fe2+-rich melts thus persist under extreme pressures and temperatures. Such compositional contrasts could contribute to lateral heterogeneities near the top of the Earth's outer-core,…."
The presence of Fe3+ in liquid iron is unlikely, given the core's low oxygen content. Although ionic and metallic liquids can coexist in the Fe–O system at relatively low pressures (<25 GPa; Frost et al., 2010), even if an ionic Fe–O liquid were present in the core, it would most likely be dominated by Fe^{2+}. The oxygen content of the core is estimated to be below ~10 wt% (Hirose et al., 2013), significantly lower than the stoichiometric composition of Fe2+O.

"…where regions of the lower mantle may be relatively enriched in Fe3+ [54, 55]"
This is the case of the solid lower mantle as shown in the references [54, 55]. The lower mantle likely contains a large amount of Fe3+, but it is not melted. This is not linked with the experimental results. The authors may discuss here the chemical reaction between the lower mantle and the core, but the solid lower mantle may not chemically react with the liquid outer core to a great extent because of the low diffusion rate in the solid (e.g., Holzapfel et al., 2005).

We thank the reviewer for these important points and corrections, and work toward improvement of the discussion of our results for Earth Sciences also advised by reviewer#1 and reviewer#2.

1/We agree that Fe3+ is unlikely to be significantly relevant for the Earth's outer-core for the following reasons mentioned by the reviewer:

Some regions of the lower mantle have been proposed to be relatively enriched in Fe3+ compared with Fe2+ (Xu et al., 2015; Frost et al., 2008). However, Fe and O diffusion in solid minerals is too slow (holzapfel et al., 2005) to generate efficient interaction between the solid lower mantle and the liquid outer-core. Exchanges could happen efficiently only in presence of melt or water, which increase diffusion coefficients. In case of interaction between water and the outer-core, Fe2+-bearing phases such as FeO (Mao et al., 2017; Kawano et al., 2024), and Fe_2OH (Hu et al., 2017; Boulard et al., 2019), are expected to be in contact with the outer-core and could possibly explain ULVZ (Mao et al., 2017) or mega-ULVZ (Kim et al., 2026). If partial melt is present in the D" layer (Garnero et al., 1996), relatively high Fe3+ content could be present as silicate melt can incorporate significant amount of Fe3+ at lower mantle conditions pressure (Kuwahara et al., 2023). However, ferric iron would likely be reduced into Fe2+ and Fe metal upon interaction with the outer-core or incorporated into silicate minerals upon recrystallization (Wood et al., 2006). Therefore, significant structural differences observed here between Fe2+ and Fe3+-rich melts are unlikely to be relevant for the Earth outer-core.

2/We note that in order to determine experimentally and reliably the Fe-O bonding environment under shock it is very difficult to do so if oxygen is highly diluted at the stage of current development of liquid diffraction under shock as so far mostly monoatomic liquid has been studied. We thus chose, as a first approach, to look at iron oxides to resolve with confidence the melt structure before going to more dilute Fe-O type composition. We however note that our results are in relative agreement with more dilute composition studied by ab-initio MD: $Fe_{0.67}O_{0.33}$ by Alfe et al., 1999 and $Fe_{0.96}O_{0.04}$ by Posner et al., 2017.

3/We added the comparison with a more dilute composition and discussed the effect of temperature. We corrected our discussion regarding the valence state of Fe and improved our discussion on the implication of our results.

A-We modify the abstract to include temperature:

At ~177GPa (~380km below the core-mantle boundary) ***and ~ 3800 K****, Fe_2O_3 melts exhibit higher Fe–O coordination, (...)*

B-We modified the discussion to include temperature and effect of oxygen concentration

p7 bottom left

These values are consistent with ab initio molecular dynamics simulations of $Fe_{0.67}O_{0.33}$ melt by Alfè et al. 1999, which reported an Fe-O CN of 4.5 and an Fe-Fe CN of 11 at 324 GPa and 6,000 K ***although our data above 247(14) GPa were collected at significantly higher temperatures as seen in Fig. 1. The effect of temperature on melt structure at such extreme pressures remains poorly constrained. However, we note that no significant changes have been observed in the Fe-O bonding environment of $Fe_{0.96}O_{0.04}$ at 135 GPa between 4,600 to 5,500 K (Posner et al., 2017).***

And p9, bottom left:

The change in Fe–O bonding environment observed here between 177(14) GPa ***(at 3,800 K)*** *and 217(14) GPa* ***(at 5,700 K) occurs at pressure and temperature conditions relatively close to the Geotherm as shown in Fig. 1*** *may therefore be of particular geophysical interest, as it corresponds to a depth of roughly 380 km below the core–mantle boundary [32].*

P7, bottom right

Ab initio molecular dynamics simulations of $Fe_{0.67}O_{0.33}$ at 324 GPa and 6,000 K [29], ***and of $Fe_{0.96}O_{0.04}$ at 330 GPa and 5,200 K*** *give Fe–Fe distances about 0.2 Å shorter than our results (Fig. 7). Distances between 2.3-2.5 Å are also reported for $Fe_{0.92}O$ at ~90 GPa and 4,000 K [34]. The slightly longer Fe–Fe bonds measured here may reflect the higher temperatures reached along the Hugoniot, where temperature increases significantly with pressure*

p8, middle right

The Fe–O distance obtained previously via ***ab-initio MD*** *for $Fe_{0.67}O_{0.33}$ at 324 GPa and 6,000 K [29],* ***and for $Fe_{0.96}O_{0.04}$ between 135 and 330 GPa and 4,600 and 5,500 K [28] are*** *also consistent with our measurements.*

C- we corrected our discussion on Fe3+ / Fe2+ :

We remove the following part following the reviewer comments:

Such compositional contrasts could contribute to lateral heterogeneities near the top of the Earth's outer-core, where regions of the lower mantle may be relatively enriched in Fe3+ compared with Fe2+ (Frost et al., Xu et al.,). In addition, the oxidation state of the primitive magma ocean and the lower mantle likely evolved through Earth's history (Wade et al., 2005),, potentially affecting the solubility of oxygen in deep planetary reservoirs.

And we added the following (p9, left):

They are, therefore, likely to be present at lower pressures as well, relevant to magma oceans (up to ~ 60 GPa for the Earth [60, 61]). The presence of Fe3+ in the early magma ocean is of particular importance for understanding the oxidation state of the Earth [62] and Super-Earths [63]. In peridotitic melt Fe2+ has been shown to act as a network modifier, while Fe3+ presents a more ambiguous role with a higher Fe-O CN [64], in agreement with our results. The differential solubility of ferric and ferrous iron within the magma ocean is a key parameter for retracing the evolution of the oxidation state of the Earth and Super-Earths.

D-we strengthened the discussion on the implications of our results:

In term of layering at mid Earth outer-core (bottom left p7)

Our results suggest that the previously proposed increase in Fe–O coordination number from 6 to 8 in liquid FeO near 240 GPa, by analogy with the B1–B2 solid-state transition in FeO, is unlikely to hold [27]. ***Furthermore, no significant change is observed in the Fe-O bonding environment from 217(14) to 315-333(14) GPa as shown in Fig. 7. Therefore, no layering is expected to result from changes in oxygen incorporation in liquid Fe between approximately 780 km below the Core-Mantle Boundary (CMB) and the inner-core boundary.***

Regarding the effect of the coordination number of Fe (p7 middle right)

This finding implies that the Fe–O bonding environment in iron oxide melts differs markedly from that in major lower-mantle Fe-bearing minerals such as ferropericlase and bridgmanite, where Fe is surrounded by 6 or 8 oxygen atoms.

It has been shown that oxygen solubility increases with pressure, allowing oxygen to be significantly incorporated into liquid Fe [55]. Generally, elements are more readily incorporated into a melt if their CN is higher than in the solid phase. Alfe et al. [29] reported Fe–O and Fe–Fe CN values similar to ours using ab initio MD simulations, estimating an average of 9 Fe atoms and 4 to 5 O atoms surrounding each oxygen atom, with potential charge transfer between O and Fe. This high oxygen solubility is thus explained by the large number of bonds formed by oxygen atoms in liquid Fe and by its small atomic radius, which allows oxygen to enter the dense network [29].

Regarding possible implication of oxygen incorporation in layering observed at the top of the Earth outer-core (p9 top right)

The change in Fe–O bonding environment observed here between 177(14) GPa ***(at 3,800 K)*** *and 217(14) GPa* ***(at 5,700 K) occurs at pressure and temperature conditions close to the geotherm as shown in Fig. 1,*** *and may therefore be of particular geophysical interest, as it corresponds to a depth of roughly 380 km below the core–mantle boundary (Dziewonski et al., 1981).* ***The increase in Fe-O interatomic distances observed above 177(14) GPa suggests a slight decrease in oxygen solubility, linked to the longer and thus weaker bonds at depths greater than 380 km below the CMB. It has been proposed that oxygen enrichment at the top of the Earth's outer-core originating from a partially molten, FeO-rich layer at the CMB could explain the observed low velocity and low density [24]. Nevertheless, even assuming a maximum diffusion coefficient of 3.3x10−8 m2/s for oxygen within liquid Fe [28], oxygen diffusing from the CMB is unlikely to reach a depth of more than 100 km, even over the entire history of the Earth. A thicker stable layer is, however, explained by a change in oxygen solubility at approximately 380 km depth, leading to higher oxygen content within the first 380 km of the outer core. If the observed change is accompanied by a slight drop in viscosity, similar to results seen in B2O3 [65], convection will not affect this outermost, more viscous layer. This mechanism therefore explains*** *the presence of a relatively thick, stratified layer inferred from seismological and geomagnetic observations.*

Nevertheless, it remains uncertain whether Fe_2O_3 is fully molten or partially amorphous at 177(14) GPa. Partial amorphization could account for the anomalously high density measured at this pressure, as shown in Fig. 8.

Sample:
The Fe + FeO sample consists of intergrown Fe + FeO (B1) crystals prepared by plasma sputtering (Methods and Fig. S2). As mentioned in the manuscript, the melting is a nanosecond phenomenon; however, the chemical diffusion of the elements can be much slower. This raises the concern that the liquid may not be chemically homogeneous during the ~10 nanosecond laser pulse (Fig S5). This should be fully discussed.

We agree with the reviewer and we now fully discussed this aspect in the paper along with providing new Scanning Transmission Electron Microscope images of the Fe + 4.5 FeO initial sample to define the grain size.

We added the STEM image to the sample description at the end of the section I of the Supplemental Material:

A thin section of the Fe + 4.5 FeO sample was prepared (from the substrate to the free surface of the deposition) using a Zeiss Auriga dual beam Focussed Ion Beam (FIB) - SEM. The prepared lamella was thinned to sub-100 nm thickness and observed using a JEOL GrandARM 300F available at the David Cockayne Centre for Electron Microscopy at the Department of Materials, University of Oxford. The instrument was operated in Scanning Transmission Electron Microscope (STEM) mode at 300 KV with a Cold Field Emission Gun, capable of atomic resolution. We observed grain size below 10 nm throughout the sample as shown in Fig. S4 and Fig. S5.

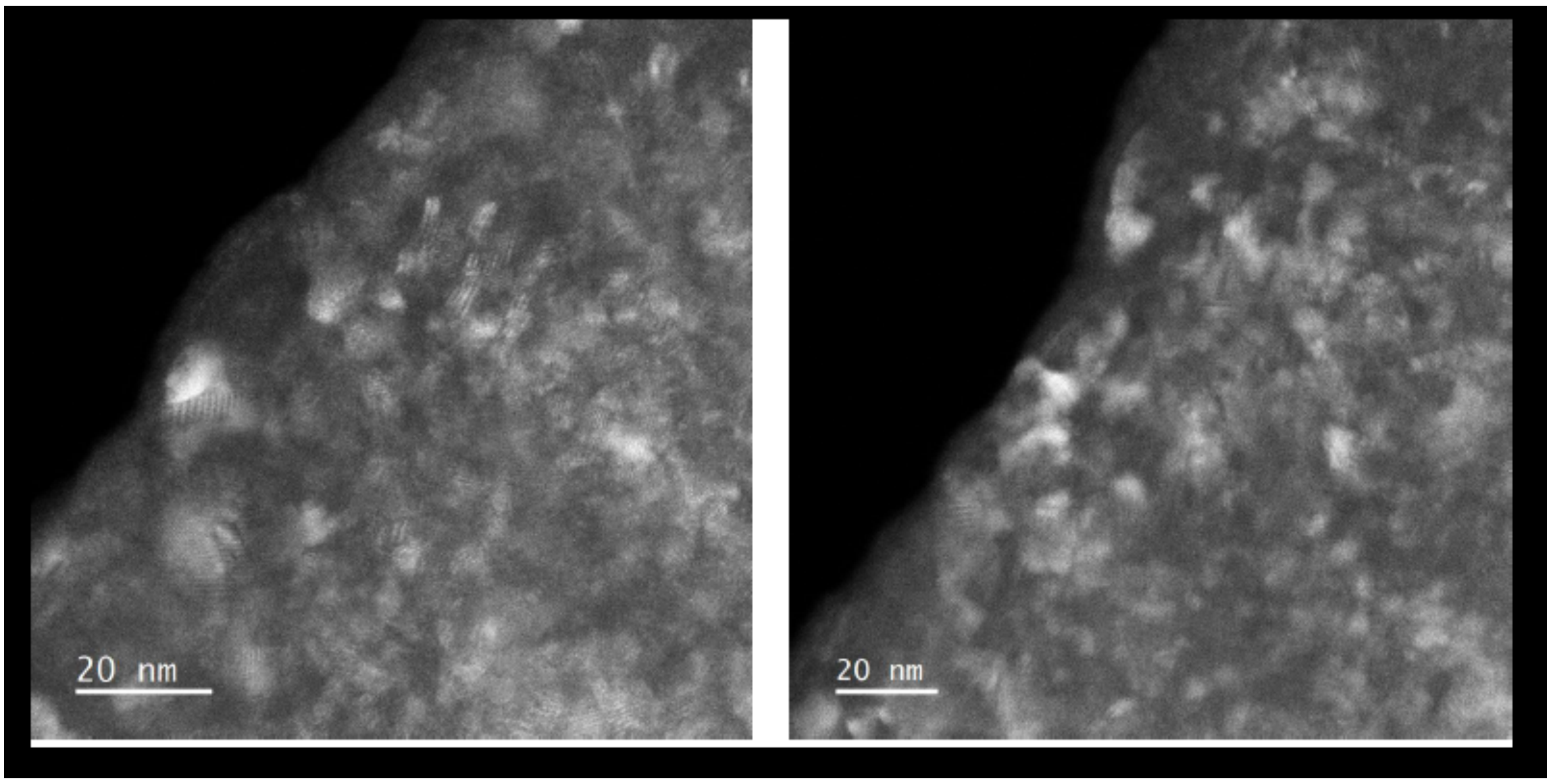

Figure S4. Annual Dark Field STEM images recorded in 2 positions on a FIB section of the initial Fe + 4.5 FeO sample. The edge shows the exposed sample surface and the substrate is towards the bottom right direction in the image.

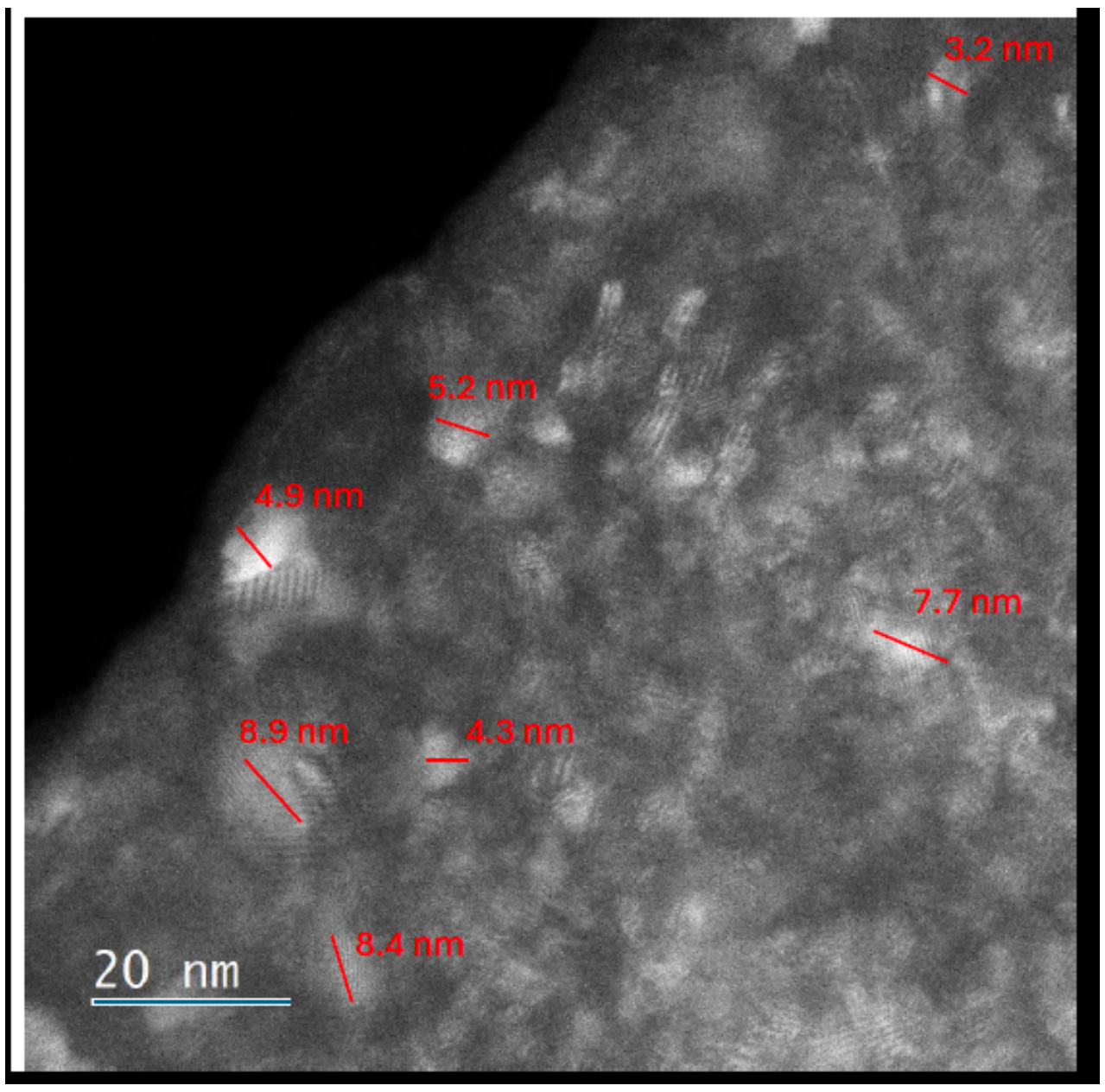

Figure S5. Few sizes of grain are shown on the annual Dark Field STEM image shown on Fig. S4 left.

We added the grain size in the main paper to confirm the presence of a single homogeneous melt (p3, right column):

Consequently, only data above 280 GPa where Fe is fully molten (see Fig. 3) are taken to represent complete Fe + 4.5 FeO melting. ***The melt signal observed above 280 GPa corresponds to a fully mixed Fe + 4.5 FeO melt, as the grain size observed on the initial sample by Scanning Transmission Electron Microscope is smaller than 10 nm (Figs. S4 and S5). This small grain size allows complete mixing of Fe and FeO melt under high pressures and temperatures, based on diffusion coefficients available from ab initio molecular dynamics simulations (Posner et al., 2017). Further discussion is provided in the Supplementary Material section XIV.***

We discussed in detail the diffusion coefficient of the atoms in a new section XIV of the Supplemental Material :

The Fe + 4.5 FeO sample was treated as a complete melt under two specific conditions:
• 330 GPa and 10,894 K – 10.4 g/cm3, for which a 10 ns laser pulse was used.
• 440 GPa and 15,172 K – 10.7 g/cm3, for which a 6 ns laser pulse was used.

These are extreme conditions, where diffusion coefficients (D) are poorly constrained. Posner et al., 2017 [42] reported, via ab initio molecular dynamics simulations, oxygen and iron diffusion coefficients in liquid Fe up to 5,200 K and 330 GPa. They found at this condition DO = 1.5 × 10−8 m2/s and DFe = 0.58 × 10−8 m2/s, in relative agreement with Pozzo et al., 2013. Currently, no diffusion coefficient is available for iron oxide melts at these conditions. To assess the effect of temperature (T) on these coefficients, we use an Arrhenius law:

$$D = A \exp\left(\frac{-Q}{RT}\right)$$

where A is the pre-exponential factor, Q is the activation enthalpy, and R is the universal gas constant. Posner et al, 2017 [42] showed that the activation energy Q for oxygen is nearly constant at 50 kJ/mol, while it increases significantly with pressure for Fe, from 50 kJ/mol up to 125 kJ/mol at 125 GPa. This implies that at 330 GPa, the activation energy for Fe reaches 248 kJ/mol. At 330 GPa, the ratio of diffusion coefficients between 10,894 K and 5,200 K is calculated as follows:

$$\ln\left(\frac{D_{10894}}{D_{5200}}\right) = \frac{Q}{R}\left(\frac{1}{5200} - \frac{1}{10894}\right)$$

Which gives:

$$D_{10894} = D_{5200} \times \exp\left[\frac{Q}{R}\left(\frac{1}{5200} - \frac{1}{10894}\right)\right]$$

Using QFe = 248 kJ/mol, we find D10894(Fe) = 11.6 × 10−8 m2/s. For oxygen, with QO = 50 kJ/mol, we find D10894(O) = 2.75 × 10−8 m2/s. To find the average distance x that atoms travel in t = 10 or 6 ns, we use the root-mean-square displacement along a line:

$$x = \sqrt{2Dt}$$

At 330 GPa and 10,894 K oxygen can travel 23 nm and iron 48 nm within 10 ns (and 18.2 nm for oxygen and 37.3 nm for iron within 6 ns). Given that the initial grain size is smaller than 10 nm, atoms at the centre of a grain need only to travel a maximum of 5 nm to mix. Since the calculated distances significantly exceed this value, we can confidently attribute the melt signal to a single homogeneous Fe + 4.5 FeO melt.

We added a coauthor who performed the FIB section and the STEM analysis.

Phani S. Karamched
Department of Materials, University of Oxford, Parks Road, Oxford OX1 3PH, UK

We added in the acknowledgements the following for the use of FIB and STEM:

The authors acknowledge use of characterisation facilities within the David Cockayne Centre for Electron Microscopy, Department of Materials, University of Oxford, alongside financial support provided by the Henry Royce Institute (Grant ref EP/R010145/1).

Other methods appear robust, including laser-shock experiments, XFEL measurements and XRD data analysis.

We added in the acknowledgements the following to better suit the convention of the European XFEL facility, which have changed since submission:

We acknowledge the European XFEL in Schenefeld, Germany, for provision of X-ray Free-Electron laser beam time at the Scientific Instrument HED (High Energy Density Science), proposal p6746 ***and would like to thank the staff of European XFEL and DESY for their assistance.***

Reviewer #1 (Remarks to the Author):

I would like to thank the authors for their diligent efforts in addressing the previous round of reviewer comments. The revised manuscript has improved in several areas. However, certain critical technical ambiguities remain. In particular, the temperature characterization and the structural discrimination between disordered phases require more rigorous treatment to meet the publication standards of Nature Communications.

We thank the reviewer for their careful and helpful comments, and we address the points they raised below.

Specific Comments

1. Quantitative Uncertainty in Temperature Measurements

While I appreciate the additional details regarding the experimental challenges of temperature measurement in shock experiments, the current discussion lacks a quantitative assessment of uncertainty. The authors state that temperatures cannot be directly extracted from the Rankine-Hugoniot relations, yet, Figure 1 displays points across a vast range (4,000–15,000 K) without temperature error bars. Given that part of their discussion e.g. the distinction between the amorphous solid and liquid phases relies on the assertion that "T is too low" for melting, a robust error analysis is essential. The authors should provide at least a semi-quantitative estimate of the uncertainty associated with their temperature assumptions (e.g., using SESAME EOS or similar models) to substantiate their phase assignments.

We agree with the reviewer's comment. Figure 1 has been updated to include error bars for temperature. These uncertainties were determined by evaluating the temperature at the lower and upper bounds of the pressure error range.

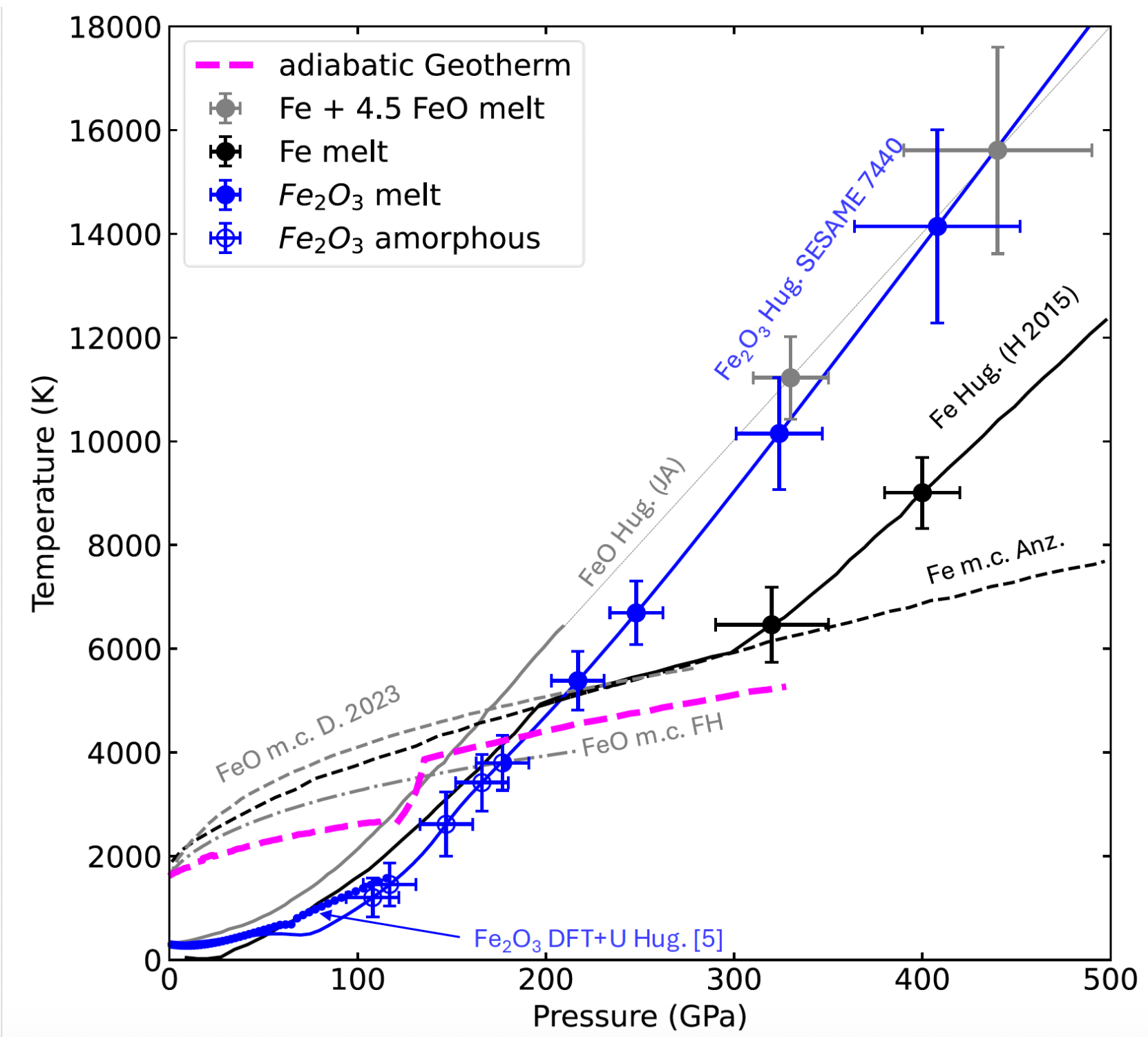

-We added in the caption of figure 1:

Temperatures are estimated from SESAME 7440 [4], consistent with previous DFT+U calculations [5]. ***Temperature error bars correspond to the temperature calculated at the lower and upper bounds of the pressure uncertainty.***

-We added the temperature uncertainties and rounded appropriately in the supplementary table 2.

-We added the error bar related to temperature throughout the text and rounded appropriately:

p2 right column

In this work we used previously calculated temperatures along the Hugoniot curve of Fe [7], FeO [6] and Fe2O3 (SESAME 7440 [4] and DFT+U [5]), resulting in temperatures between ***3,800(500)–16,000(2,000)*** *K, as shown in Fig. 1.* ***Temperature error bars given in the text corresponds to the temperature calculated at the lower and upper bounds of pressure uncertainty.***

p5 right column top

The Fe2O3 data point at 177(14) GPa and ***3,800(500) K***

p7 left column bottom

We resolve the Fe–O bonding environment between 177–438 GPa, and ***3,800(500)–14,000(2,000)*** *K based on temperature estimates from SESAME 7440 [4] for Fe2O3,*

p8 left column bottom

At 378–438(14) GPa and ***14,000(2,000) K,***

p9 right column top

The change in Fe–O bonding environment observed here between 177(14) GPa ***(3,800(500) K) and 217(14) GPa (5,400(700) K)***

-We added the temperature error bars in the Supplemental Material – section XIV. Consequently, the temperature values were rounded appropriately and all calculations updated.

The Fe + 4.5 FeO sample was treated as a complete melt under two specific conditions:

• 330 GPa and ***11,200(800) K*** *– 10.4 g/cm3, for which a 10 ns laser pulse was used.*

• 440 GPa and ***16,000(2,000) K*** *– 10.7 g/cm3, for which a 6 ns laser pulse was used.*

-We improve our discussion in link with the amorphization observed at 108(14) GPa by providing a more detailed discussion on the temperature estimate when diffuse scattering appears p4 right column:

This is consistent with an earlier study, where amorphization occurred at 122(3) GPa [5], ***at temperatures too low to induce equilibrium melting****.* ***At 108(14) GPa temperature is estimated to be 1,200(400) K according to SESAME 7440 [4] and 1,400(300) K according to DFT+U (Crepisson et al., PRB): this temperature remains below 1873 K, the melting point of Fe_2O_3 at ambient conditions (Biswas, 1981), and thus melting is unlikely.***

2. Clarity on Multi-state Probing in X-ray Diffraction (XRD)

The authors note that the XRD patterns likely reflect a superposition of multiple states of matter sampled simultaneously during the shock. This is a critical point for the interpretation of the data. Given the broad, interdisciplinary readership of Nature Communications, even though this is a “standard practice”, this nuance must be explicitly stated and discussed in the main text to ensure the findings are accessible to those outside the specialized XFEL community.

We agree with the reviewer and we added the following sentence, before presentation of the results in the main paper to emphasize the origin of the remaining ambient peaks in the data p2 bottom and 3 top:

The XRD intensities are fully corrected for solid angle, polarization, the different apparent thickness due to different angles of the Al filter placed over the detectors, self-attenuation, ablator contribution, and residual ambient material, as detailed in the Methods section. ***We note that residual ambient Bragg peaks can still be seen in the azimuthally integrated 1D XRD lineouts as we probed the sample at maximum compression but before complete release, resulting in a small unshocked fraction still appearing in the data.***

3. Le Bail Analysis and Comparative Data
Regarding the Le Bail refinement of the observed features: the authors argue that similar features were observed in their previous work. However, the existence of prior observations does not preclude the need for a comprehensive structural discussion in the current manuscript. I request that the authors include a figure comparing the current data with previous results or provide a representative Le Bail fit to justify their structural assignments.

Following the referee’s suggestion, we have performed Le Bail refinement of our ambient Fe_2O_3 sample, of the alpha phase at 33(14) GPa, and of the alpha prime phase at 82 (14) GPa to justify the assignment of the phases.

We specify in the main text p4 left column :

In Fig. 5, we see that Fe2O3 transforms from the α to the α'-Fe2O3 phase below 97(14) GPa. ***Supporting le Bail refinements are provided in Figs. S14, S15, S16 of the Supplemental Material.***

We added the following to the supplementary materials in section VIII:

We performed Le Bail refinement on the solid phases of Fe_2O_3, at ambient conditions, and under shock at 33(14) GPa and 82(14) GPa. It is not possible to perform Rietveld refinements on the data due to the complex microstructure of the samples under shock. Refinements shown in Figs. S14, S15, S16 indicate that the reflections observed are well explained by an α to α'- Fe_2O_3 phase transition similarly to previous study of Fe_2O_3 under laser-driven shock compression [27]. None of the phase transitions reported under static compression [28] are observed. The presence of a small Al contribution is due to the approximately 200 nm coating on the target. One data point presents both α and α'- Fe_2O_3 phases: *hydrodynamic simulations showing pressures of 42 and 64 GPa (Fig. S14). We report cell parameters in Table 1 of the Supplemental Material. We can see that at 97(14) GPa, several α'- Fe_2O_3 Bragg peaks disappeared in link with amorphization under shock at around 108(14) GPa (Fig. S14).*

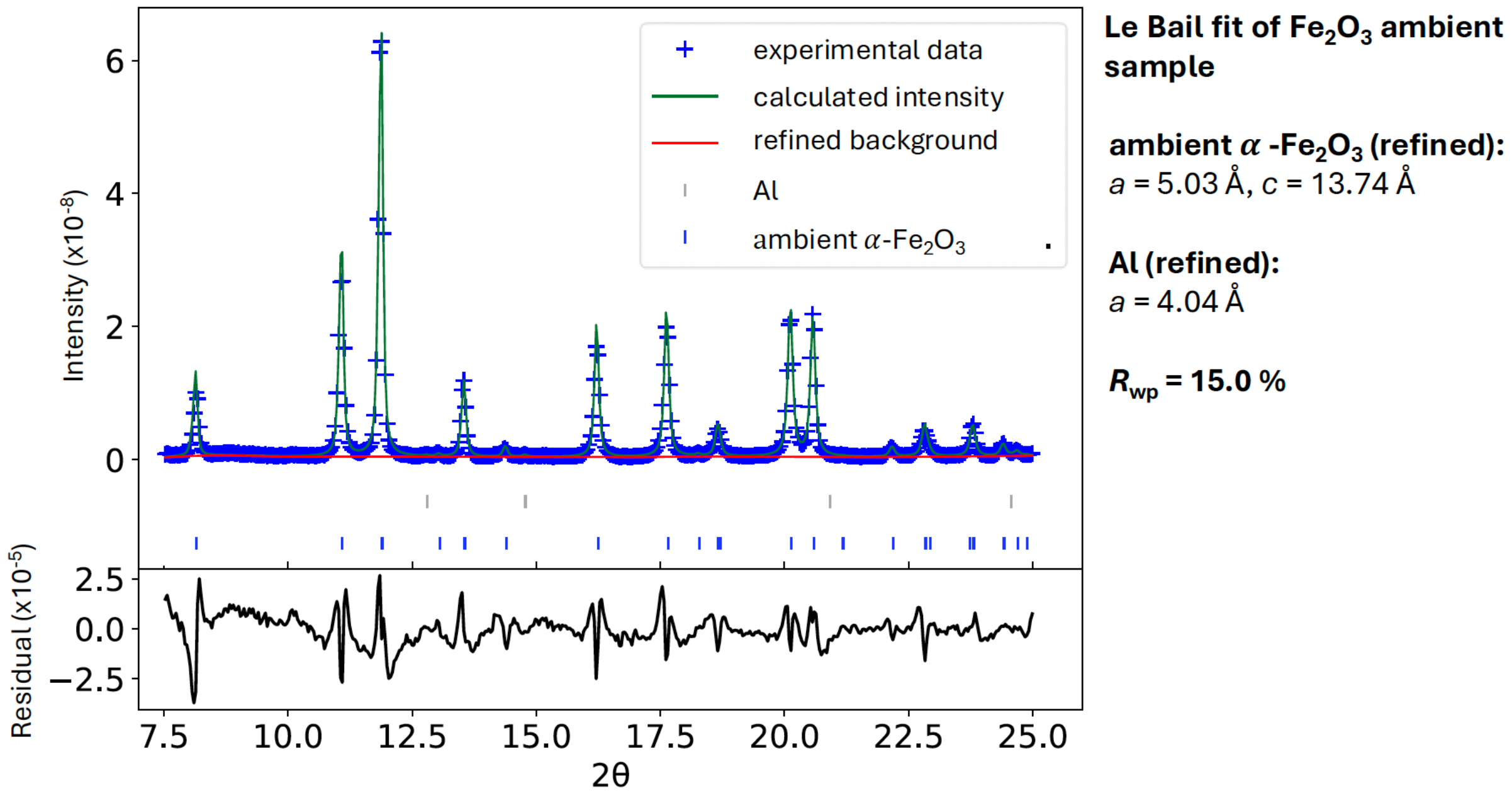

Fig S14. Le Bail refinement of ambient sample performed on azimuthally integrated 1D XRD lineout after combining the two VAREX detectors and correcting the XRD intensity, as detailed in the Methods section C of the main paper. Fitted cell parameters of α- Fe_2O_3 phase and Al are given, along with weighted profile R-factor (R_{wp}) of the fit.

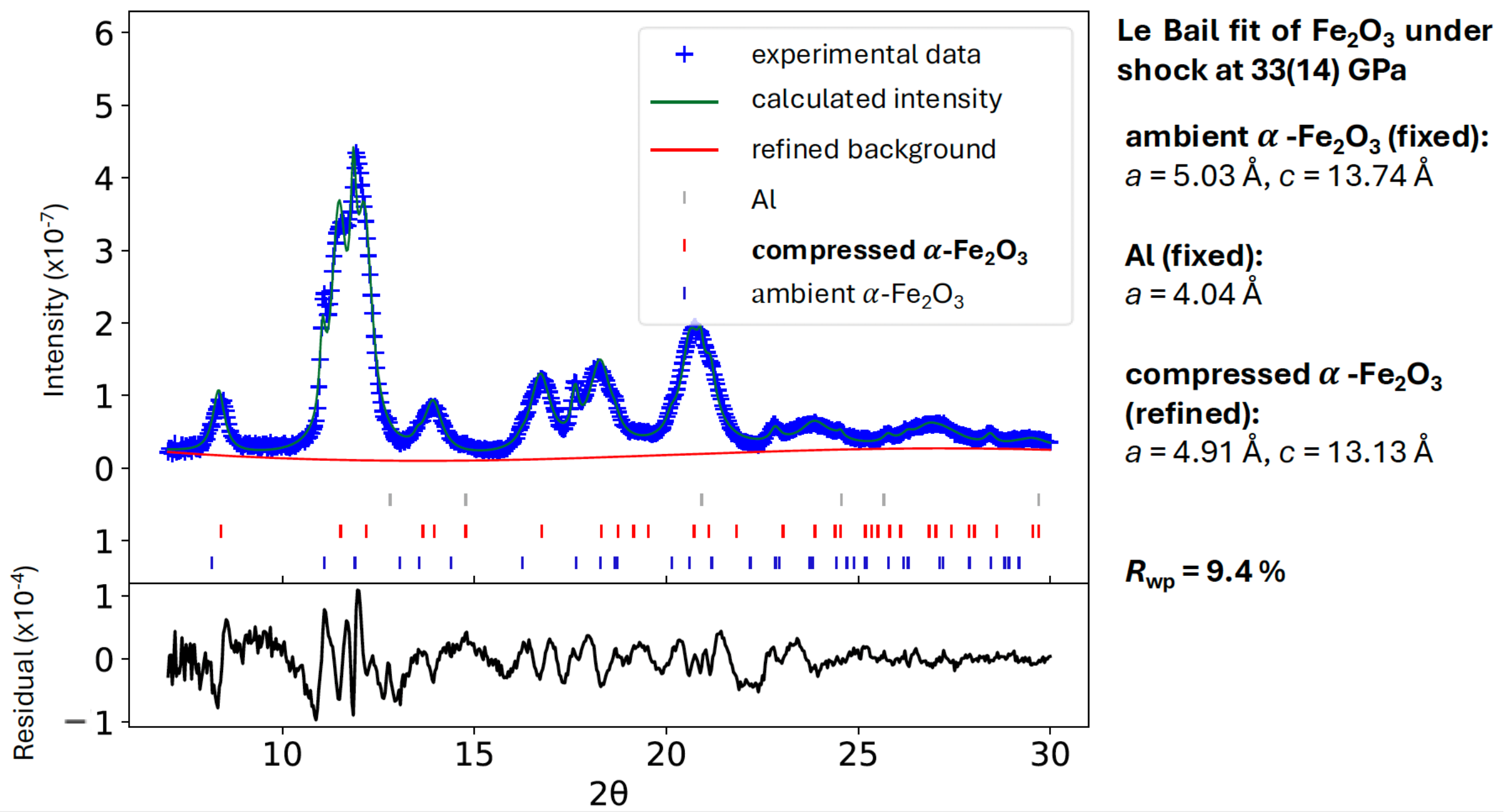

Fig S15. Le Bail refinement of shocked sample at 33(14) GPa performed on azimuthally integrated 1D XRD lineout after combining the two VAREX detectors and correcting the XRD intensity, as detailed in the Methods section C of the main paper. Cell parameters of ambient α- Fe_2O_3 phase and Al are fixed to the value found by Le Bail refinement of the ambient sample (Fig. S14). Fitted cell parameters of compressed α- Fe_2O_3 phase are given, along with weighted profile R-factor (R_{wp}) of the fit.

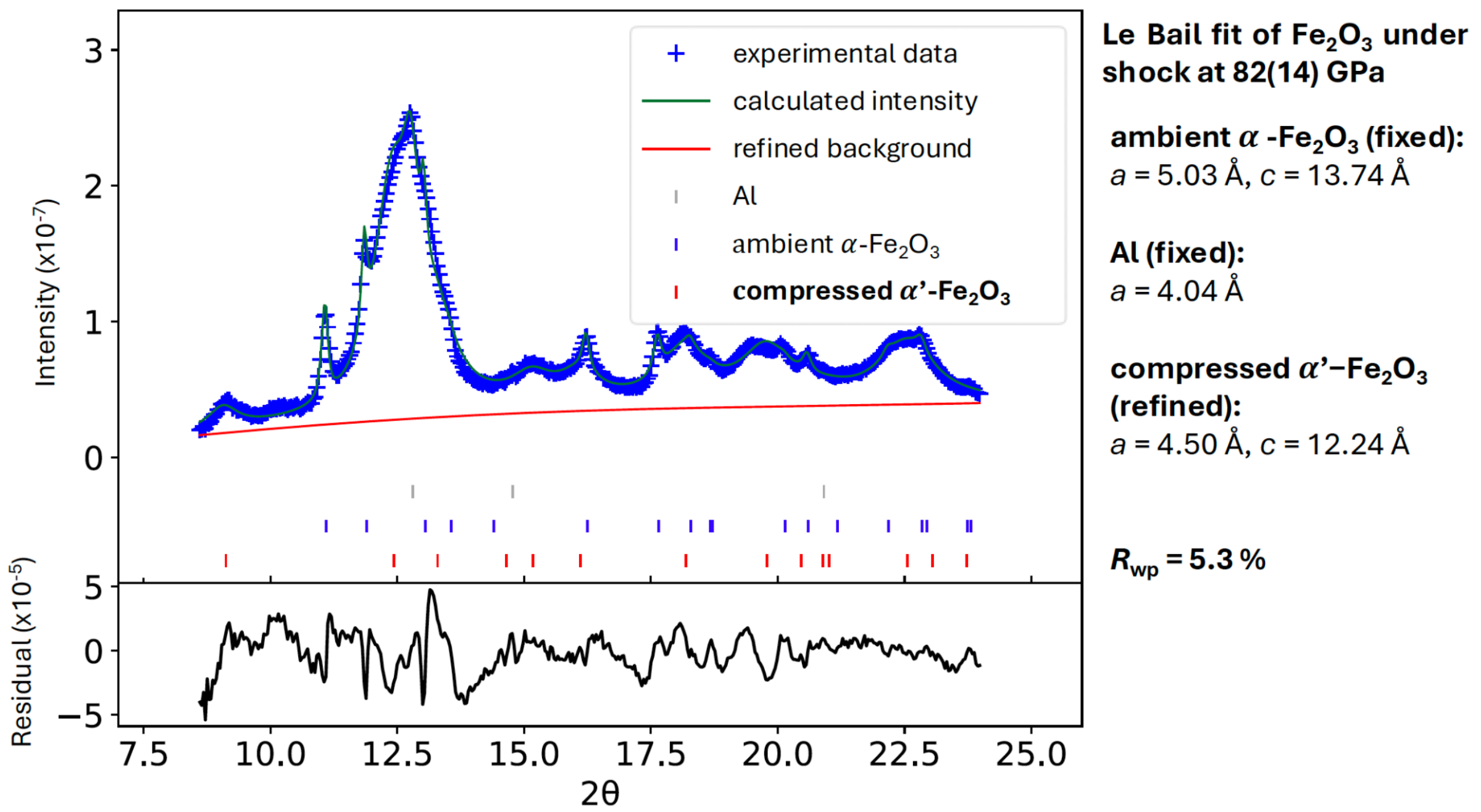

Fig S16. Le Bail refinement of shocked sample at 82(14) GPa performed on azimuthally integrated 1D XRD lineout after combining the two VAREX detectors and correcting the XRD intensity, as detailed in the Methods section C of the main paper. Cell parameters of ambient α- Fe_2O_3 phase and Al are fixed to the value found by Le Bail refinement of the ambient sample (Fig. S14). Fitted cell parameters of compressed α'- Fe_2O_3 phase are given, along with weighted profile R-factor (R_{wp}) of the fit.

We added Le Bail refinement results in Table 1 of the Supplemental material.

4. Data Justification in the Absence of VISAR Diagnostics
I have concerns regarding the inclusion of data points at 82 and 147 GPa (Figure 4) given the authors' admission that no VISAR data was retrieved for these runs. Without velocimetry to constrain the pressure-temperature conditions, it is unclear how these points can be reliably interpreted or compared to the rest of the dataset. The authors should clarify the diagnostic basis for including these specific points.

As detailed in the Method section and in the supplementary material and table we use both hydrodynamic simulations and VISAR analysis to constrain the pressure. Hydrodynamic simulations being standard to determine pressure conditions in shock experiments. In section E of the Methods we previously wrote:

Hydrodynamic simulations were performed using the code MULTI [74] to independently estimate the shock pressures in Fe_2O_3 and Fe + 4.5 FeO. […] A numerical intensity factor (~0.5–0.7) was applied to the experimental laser intensity to match the measured breakout times. The black Kapton ablator was again modelled using SESAME 7770. <u>When breakout times were unavailable, the intensity multiplier from the closest measured shot was adopted.</u>

We modified our explanation for better clarity:

When breakout times were unavailable, ***we use the intensity multiplier determined on the closest shot performed at similar laser conditions (detailed in Table 1 of the Supplemental Material).***

5. Discrimination Between Liquid and Amorphous Solid Phases
The structural discrimination between the liquid and amorphous solid states remains a

point of concern. In a true liquid, the Pair Distribution Function should show a rapid decay of oscillations beyond the first coordination shell due to the loss of medium-range order. Conversely, an amorphous solid—while lacking long-range periodicity—retains a "frozen" rigid network that typically manifests as sharper, more persistent peaks at higher r-values. I invite the authors to provide a more detailed analysis of the PDF peak widths and the evolution of the second and third coordination shells to support their claim of an amorphous solid state.

As previously said in point 1/ the diffuse scattering observed at 108(14) GPa cannot be linked to a melting as the temperature under shock at this pressure is lower (even including error bar) than the melting temperature of Fe_2O_3 at ambient pressure. In a previous paper we calculated DFT+U Hugoniot pressure / temperature curve and showed a relative agreement with SESAME 7440, which is also visible in Fig. 1 (Crepisson et al., 2025, PRB). We detailed this point p 4 right column:

This is consistent with an earlier study, where amorphization occurred at 122(3) GPa [5], ***at temperatures too low to induce equilibrium melting****.* ***At 108(14) GPa temperature is estimated to be 1,200(400) K according to SESAME 7440 [4] and 1,400(300) K according to DFT+U (Crepisson et al., PRB): this temperature remains below 1873 K, the melting point of Fe_2O_3 at ambient conditions (Biswas, 1981), and thus melting is unlikely.***

We agree that data points above 117(14) GPa have a temperature which is above 1873 K and that this argument might not be enough. However we discuss the following in the paper (p5 right column):

- no change is observed in the position of the main amorphous peak from 108(14) to 166(14) GPa in terms of height or width of the diffuse scattering. Moreover this peak remains sharp and only starts decreasing and broadening only from 177(14) GPa

The position of the main amorphous peak at ~2.74 Å^{-1} remains constant from 108(14) to 166(14) GPa, consistent with previous observations for amorphous Fe_2O_3 [5].

Between 166(14) and 177(14) GPa, this peak broadens and decreases markedly in intensity, accompanied by the disappearance of all Bragg reflections.

-when studying 2 different Fe_2O_3 samples (displayed in fig. 5) we can see that the diffuse scattering signal is different at 135 / 147 GPa while similar at 248 / 253 GPa which is a strong argument in favour of an amorphous phase at 135/147 GPa.

At 135–147(14) GPa, the samples display distinct diffraction signatures, consistent with an amorphous or partially amorphous phase that retains features of the original crystallinity.

With the present dataset we cannot provide a full liquid diffraction analysis in terms of pair distribution function for data below 177(14) GPa due to the low number of runs taken at this pressure (1 or 2 only) combined with the presence of residual solid peaks and ambient peaks which make it difficult to isolate the diffuse scattering signal reliably. For these reasons we did not perform liquid diffraction analysis on amorphous data from 108(14) to 166(14) GPa.

Reviewer #3 (Remarks to the Author):

All my concerns have been properly addressed, and I am now able to recommend this manuscript for publication.
